# A global meta-analysis of gonorrhoea and chlamydia prevalence among men who have sex with men from 2000 to 2022

**DOI:** 10.1177/09564624251333489

**Published:** 2025-04-21

**Authors:** Ella P Davies, Motoyuki Tsuboi, Jayne Evans, Jane Rowley, Eline L Korenromp, Tim Clayton, R Matthew Chico

**Affiliations:** 1 Department of Disease Control, Faculty of Infectious and Tropical Diseases, 218289 London School of Hygiene and Tropical Medicine, London, UK; 2 Bureau of International Health Cooperation, National Center for Global Health and Medicine, Tokyo, Japan; 3 Global HIV, Hepatitis and STIs Programme, WHO, Geneva, Switzerland; 4 30586 UNAIDS, Data for Impact, Strategic Information to Close Inequalities team, Geneva, Switzerland; 5 Department of Medical Statistics, Faculty of Epidemiology and Population Health, 218289 London School of Hygiene and Tropical Medicine, London, UK

**Keywords:** Chlamydia (Chlamydia trachomatis), bacterial disease, Gonorrhea (Neisseria gonorrhoeae), bacterial disease, epidemiology, other, homosexual, other, trichomoniasis (Trichomonas vaginalis), Protozoal disease

## Abstract

**Introduction:**

We conducted a global systematic review and meta-analysis of gonorrhoea and chlamydia among men who have sex with men (MSM) from 2000 to 2022.

**Methods:**

We searched four databases to identify studies conducted between 1 January 2000 and 19 April 2022 that reported prevalence from aetiological assays. We extracted data, calculated point estimates, corrected and then pooled them using random-effects models. We stratified results by United Nations regions and conducted subgroup analyses established *apriori.*

**Results:**

172 studies met our inclusion criteria, providing 387 prevalence data points from 57 countries. The overall pooled prevalence for gonorrhoea was 7.2% [95% CI: 6.0 to 8.5; 188 data points; *n* = 347,253] and for chlamydia was 9.9% (95% CI: 8.8 to 11.0; 190 data points; *n* = 342,799). For gonorrhoea, pooled prevalence between 2000 and 2010 was 5.0% (95% CI: 3.7 to 6.5; 89 data points; *n* = 78,557) compared to 9.3% (7.7–11.1; 99 data points; *n* = 268,696) between 2011 and 2022, *p* < 0.001. For chlamydia, pooled prevalence between 2000 to 2010 was 6.6% (95% CI: 5.4 to 7.9; 95 data points; *n* = 91,015) compared to 13.6% (12.0–15.2; 95 data points; *n* = 251,784) between 2011 and 2022, *p* < 0.001.

**Conclusion:**

A holistic approach is needed to reduce the curable STIs burden among MSM.

## Introduction

Gay men and other men who have sex with men (MSM) are among five key population groups that are particularly vulnerable to human immunodeficiency virus (HIV) and other sexually transmitted infections (STIs), and frequently lack adequate access to services due to biological, behavioural and structural factors.^1–3^ *Neisseria gonorrhoeae* and *Chlamydia trachomatis* are two of the most common STIs in the world.^4,5^ Untreated STIs, symptomatic or not, can lead to a range of clinical sequelae in men including epididymitis and infertility^6–8^ and may facilitate the transmission and acquisition of HIV.^9^ Because these STIs are more commonly asymptomatic, individuals who are infected are often unaware and, therefore, do not seek treatment.^8^ Gonococcal case management of pharyngeal infections is particularly difficult. A curative course of treatment for genital or rectal infection may not be absorbed sufficiently to clear gonococcal colonies present in the pharynx.

World Health Organization (WHO) global estimates of the prevalence of gonorrhoea, chlamydia, and trichomoniasis in 2020 among adult men (15–49 years of age) were 0.7% (95% confidence intervals [CI]: 0.3–1.1), 2.5% (95% CI: 1.8–3.4) and 0.5% (95% CI: 0.3–0.8), respectively.^3^ No global estimates of these infections have been generated previously for MSM, although studies in the United States,^10,11^ Latin America^12^ and Europe^13,14^ have shown the burden of gonorrhoea and chlamydia infections disproportionately fall upon MSM, underscoring the importance of targeting this population.^15^ We undertook this systematic review and meta-analysis to characterise the global and regional prevalence estimates of gonorrhoea and chlamydia among MSM between 2000 and 2022. This is a companion study to the 2021 global review and meta-analysis of syphilis among MSM in which we found the prevalence to be 14-fold higher than in the general male population.^16^ Our findings aim to provide policymakers and programme managers with information on STI prevalence for purposes of advocacy, resource planning and allocation for MSM-focused interventions.

## Methods

### Registration and searches

We followed the Preferred Reporting Items for Systematic Reviews and Meta-Analyses (PRISMA) 2020^17^ and registered our systematic review in the PROSPERO database (CRD42019127530). We then searched four databases – MEDLINE, Embase, African Index Medicus, and *Latino Americana em Ciências da Saúde* (LILACS; Latin American and Caribbean Health Sciences Literature) – for publications containing prevalence data points for gonorrhoea, chlamydia, or trichomoniasis among MSM. Supplementary file 1.1 contains a sample search strategy including medical subject headings (MeSH) terms and full-text search terms. We conducted these searches in a serial manner with the individual names of 235 countries and territories worldwide based on Sustainable Development Goals (SDG) regional groupings as shown in Supplementary file 1.2. We also reviewed reference lists of each full-text article to identify any additional studies for inclusion.

### Eligibility criteria

We restricted our search for studies published between 1 January 2000 to 19 April 2022 and applied eligibility criteria defined a priori as presented in Supplementary file 1.3. We included studies that reported prevalence data from MSM measured using at least one biological diagnostic assay without language restrictions. We excluded studies using serological testing due to its poor test performance for diagnosing active infections. We excluded studies that exclusively collected data from high-risk MSM groups. By excluding studies of MSM who were only living with HIV, only intravenous drug-users, or only symptomatic cases (defined as any symptomatology consistent with STI infection), we were able to avoid potential selection bias and produce results that are more representative of the general MSM population. The analysis did include studies that included MSM who were both living with and without HIV.

### Data extraction

The first author (EPD) screened titles and abstracts of articles against eligibility criteria and extracted information onto a standardised data extraction form. Separately, two co-authors (MT and JE) reviewed all data extraction forms alongside full-text articles, cross-referencing information for accuracy, validity, and completeness. Any discordance between the extraction form and the full-text articles were discussed and if consensus could not be achieved, a senior author (RMC) made a final determination. We extracted the following information from each report: study year(s), study design, study setting (community vs clinic), participant age, definition of MSM used by authors, sex assigned at birth, self-reported sexuality, number of individuals tested, number of individuals positive, reported prevalence, diagnostic test used, anatomical site tested, and if undertaken, results of antimicrobial resistance testing. If reports contained prevalence data for more than one anatomical site, we extracted all data points. We used the highest data point reported across anatomical sites when generating overall pooled prevalence estimates. If studies published single data points based on testing multiple anatomical sites, we used this composite value.

### Quality assessment

We used AXIS - the Appraisal tool for Cross-Sectional Studies - to assess the risk of bias within studies (high risk of bias [range 0–10] vs low [range 11-20] vs other [cohort and interventional] studies reporting prevalence data which AXIS was not applicable to) based on predetermined quality indicators as detailed in Supplementary file 1.4.^18,19^

### Meta-analyses

After data extraction, we corrected for the type of diagnostic test using the sensitivities and specificities of individual assays,^20^ following previously published methods.^16,21–23^ Supplementary file 1.5 provides more information on the sensitivities and specificities of each assay. For assays with a range of sensitivities or specificities, we applied the midpoint to generate corrected point estimates. We then applied random-effects models to our corrected data points using a statistical programme developed specifically for meta-analyses of proportions that applies the binomial distribution to model within-study variability and the Freeman-Tukey double arcsine transformation to stabilize variances^24^ to produce pooled prevalence estimates and 95% CIs by infection. We then stratified results by SDG region. We assessed study heterogeneity with the χ^2^ with Cochrane’s Q statistic quantified with the I^2^ statistic. For the analysis a random-effects model was chosen to account for the suspected global heterogeneity between studies. We completed funnel plot analyses to assess publication bias using random and fixed effect models. We used Stata/IC 16.1 for all analysis.

### Subgroup analysis

We assessed the potential effect of eight study characteristics selected a priori as part of our pooled subgroup analyses: (i) MSM population type (‘MSM studies cohorts’ which were not exclusively subgroup populations or where subgroup populations were not specified, vs ‘Other subgroup populations only’, including exclusively male sex workers [MSWs] and transgender women [TGW]) and (ii) study type (cross-sectional study vs ‘other study types’ including cohort and intervention studies that reported baseline data). We stratified by study type to see if there was a difference in prevalence between cross-sectional studies and the ‘other study type’ arm reflecting the longer duration of contact between MSM and study team within cohort studies and randomised controlled trials and therefore possibly reduced barrier to access care. We also assessed (iii) study setting (clinic only vs community only or community plus clinic combined), (iv) time period (2000–2010 vs 2011–2022), (v) anatomical site of sampling (genital, pharyngeal, and/or rectal), (vi) risk of study bias based on the Appraisal tool for Cross-Sectional Studies (AXIS, high risk [range 0–10] vs low risk [range 11-20], (vii) sample size (500 data points or fewer vs more than 500), and (viii) MSM age stratified as equal to or below the median versus above the median.

## Results

**Figure 1.**
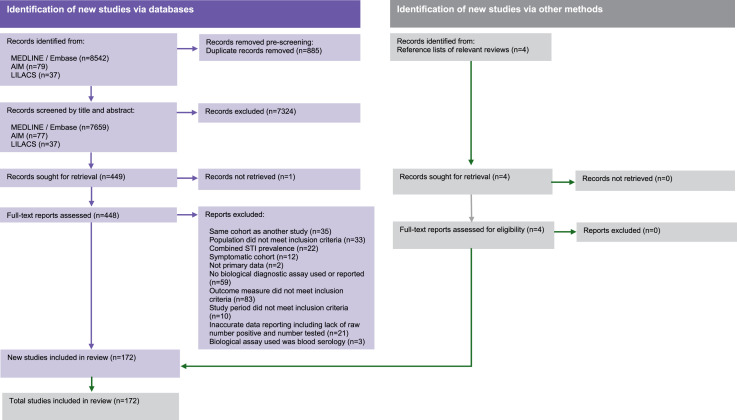
PRISMA flowchart showing the selection process for included studies in our global systematic review and meta-analysis on gonorrhoea and chlamydia among men who have sex with men from 2000 to 2022.

Our searches yielded 8658 records for review, of which 172 met the inclusion criteria shown in Figure 1. In total, 375,144 MSM between 12 and 83 years old were tested for at least one of the three STIs. Study sample sizes ranged from 9 to 139,719 MSM. For gonorrhoea, 157 studies contributed 188 data points from 347,253 MSM across 53 countries. For chlamydia, 159 studies contained 190 data points from 342,799 MSM in 53 countries. In total for gonorrhoea and chlamydia there were 184 and 194 data points included from studies conducted between 2000-2010 and 2011-2022, respectively. The distribution of included data points by study year and SDG region is described in Supplementary file 3. We identified just nine data points of trichomoniasis prevalence among MSM. We provide study-level summaries of the data points we extracted and corrected in Supplementary File 2.3 without conducting further analyses.

We calculated regional pooled estimates where there were two or more data points for gonorrhoea and chlamydia. Individual data points are detailed in Supplementary files 2.1, 2.2 and 2.3. For countries with more than two data points (*n* = 30), we calculated national pooled prevalence estimates as illustrated in Supplementary file 3 included studies described a wide range of definitions of MSM, from men who self-identify as MSM, MSW and/or TGW to all men who ever reported any sexual act with other men, which are described in Supplementary file 1.8.

### Meta-analysis

**Figure 2.**
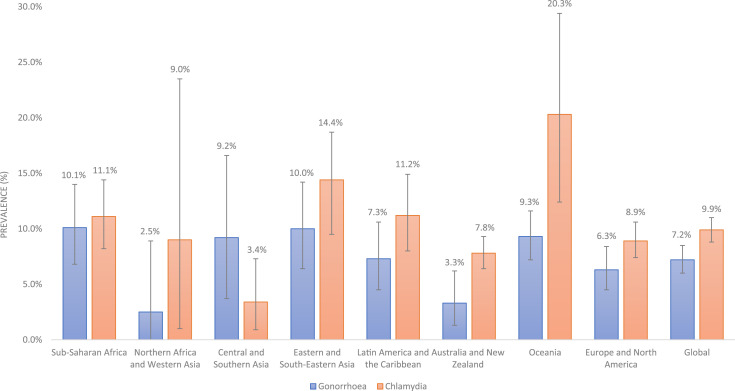
Pooled prevalence estimates of gonorrhoea and chlamydia among men who have sex with men from 2000 to 2022 by region of the Sustainable Development Goals.

Among MSM from 2000 to 2022, the global pooled prevalence of gonorrhoea was 7.2% (95% CI: 6.0 to 8.5; 188 data points; *n* = 347,253) and of chlamydia was 9.9% (95% CI: 8.8 to 11.0; 190 data points; *n* = 342,799). These are shown in Table 1 and Figure 2 along with pooled prevalence estimates by SDG region. Data points and pooled prevalence estimates with 95% CIs are presented in global and regional forest plots in Supplementary file 4. In seven of eight SDG regions, the prevalence of chlamydia was higher than gonorrhoea. Table 2 and Table 3 contain the results of the subgroup analysis for gonorrhoea and chlamydia, respectively. For gonorrhoea and chlamydia, there are differences in prevalence by study time period, study type and anatomical site of sample collection. Heterogeneity was high within regions and subgroups. All models had an I^2^ value for heterogeneity above 90%.

**Table 1.** Pooled prevalence estimates for gonorrhoea and chlamydia among men who have sex with men from 2000 to 2022 by regions of the Sustainable Development Goals.

Region	Corrected pooled prevalence (%) (95%CI)	No. of MSM		Sample size range	No. of			Heterogeneity *I*^2^ (%)
		Positive	Tested		Countries	Studies	Point estimates	
Gonorrhoea								
Sub-Saharan Africa	10.1 (6.8-14.0)	884	7,577	40-1,447	12	18	22	96.1
Northern Africa and Western Asia	2.5 (0.0-8.9)	63	1,512	210-1,064	2	3	3	93.3
Central and Southern Asia	9.2 (3.7-16.6)	314	7,116	16-2,025	2	6	13	98.7
Eastern and South-Eastern Asia	10.0 (6.4-14.2)	1,451	13,275	38-1,695	8	23	27	98.1
Latin America and the Caribbean	7.3 (4.5-10.6)	857	10,095	12-1,409	11	23	30	96.9
Australia And New Zealand	3.3 (1.3-6.2)	3,823	46,818	71-13,780	2	15	16	99.5
Oceania	9.3 (7.2-11.6)	123	1,596	100–733	1	2	4	52.5
Europe and North America	6.3 (4.5-8.4)	32,379	259,264	27-139,719	15	67	73	99.7
**Total**	**7.2 (6.0–8.5)**	**39,894**	**347,253**	**12-139,719**	**53**	**157**	**188**	**99.4**
Chlamydia								
Sub-Saharan Africa	11.1 (8.2-14.4)	831	7,577	40–1447	12	18	22	94.1
Northern Africa and Western Asia	9.0 (1.0-23.5)	74	1,512	210-1,064	2	3	3	97.7
Central and Southern Asia	3.4 (0.9-7.3)	219	6,909	16-2,025	2	4	11	97.7
Eastern and South-eastern Asia	14.4 (10.5-18.7)	1,941	12,848	38-1,695	8	21	25	97.6
Latin America and the Caribbean	11.2 (8.0-14.9)	1,138	9,965	13-1,409	12	24	27	96.5
Australia And New Zealand	7.8 (6.4-9.3)	5,078	56,770	34-13,798	2	15	20	97.1
Oceania	20.3 (12.4-29.4)	232	1,596	111–733	1	2	4	93.9
Europe and North America	8.9 (7.4-10.6)	27,324	245,622	9-139,718	14	72	78	99.2
**Total**	**9.9 (8.8–11.0)**	**36,837**	**342,799**	**9-139,718**	**53**	**159**	**190**	**98.8**

**Table 2.** Sub-group analyses of global pooled prevalence estimates of gonorrhoea and chlamydia among men who have sex with men from 2000 to 2022.

Sub-groups	No. of MSM positive	No. of MSM tested	Corrected Pooled prevalence (%) (95%CI)	Median no. of positive diagnoses	Study sample size range	No. of countries	No. of data points	Sub-group heterogeneity *p*-value**
Gonorrhoea								
MSM population								*p* = .207
MSM studies	37,847	328,405	7.0 (5.7-8.4)	29	12-139,718	50	169	
Other subgroup populations only, including exclusively MSW, TGW, and/or TGWSW only	2,047	18,848	9.1 (6.0-12.8)	12	16-13,780	12	19	
Study type								*p* < 0.001
Cross-sectional	12,887	178,000	6.1 (5.0-7.4)	24	12-16,189	49	147	
Other study type	27,007	169,253	11.2 (9.1-13.5)	52	24-139,718	20	41	
Study setting								*p* = 0.006
Clinic	35,504	280,846	9.1 (7.2-11.2)	35	12-139,718	30	86	
Community or community and clinic	4,390	66,407	5.7 (4.4-7.3)	23	16-4,822	42	102	
Time period								*p* < 0.001
2000-2010	4,184	78,557	5.0 (3.7-6.5)	17	12-12,457	30	89	
2011-2022	35,710	268,696	9.3 (7.7-11.1)	36	24-139,718	40	99	
Anatomical site								*p* < 0.001
Genital	1,221	36,823	4.2 (2.8-5.8)	7	12-5,497	25	49	
Pharyngeal	2,806	43,849	0.3 (0.0-1.4)	76	189-13,111	7	18	
Rectal	2,886	43,082	8.8 (7.4-10.4)	25	16-9,534	27	62	
Two or more sites	32,981	223,499	11.9 (10.7-13.1)	60	86-139,718	30	59	
AXIS score classification								*p* = .219
High risk of bias	1,604	18,739	9.3 (5.8-13.6)	21	24-4,925	21	26	
Low risk of bias	38,290	328,514	6.9 (5.6-8.2)	28	12-139,718	44	162	
Sample size								*p* = .006
Less than 500	2,451	24,918	8.6 (6.9-10.5)	16	12–500	46	112	
500 or more	37,443	322,335	5.4 (3.9-7.2)	79	508-139,718	26	76	
Median age^a^								*p* = .712
27 years old or younger	4941^a^	47,341^a^	8.1 (5.8-10.7)^a^	30	40-13,780	22	38	
Older than 27	3171^a^	45,167^a^	6.8 (4.9-9.1)^a^	52	65-9,534	18	33	
**Total**	**39,894**	**347,253**	**7.2 (6.0–8.5)**	**26**	**12-139,719**	**53**	**188**	

MSW = Men who have sex with men; TGW = Transgender women; TGW = Transgender women sex workers; AXIS = Appraisal tool for Cross-Sectional Studies (risk of bias assessment)- high risk of bias 0-10, low risk of bias 11-20; NC = not calculated.

^a^Data points for median age reported in 38% of gonorrhoea point estimates (71/188) and 42% of chlamydia point estimates (80/190).

**p-values were from univariate analysis. Note that all *I*^2^ measurements of heterogeneity were greater than 95%.

**Table 3.** Sub-group analyses of global pooled prevalence estimates of gonorrhoea and chlamydia among men who have sex with men from 2000 to 2022 (continued).

Sub-groups	No. of MSM positive	No. of MSM tested	Corrected pooled prevalence (%) (95%CI)	Median no. of positive diagnoses	Study sample size range	No. of countries	No. of data points	Sub-group heterogeneity *p*-value**
Chlamydia								
MSM population								*p* = .458
‘Subgroup populations only’, including exclusively MSW, TGW, and/or TGWSW only	1,960	18,467	8.1 (4.3-13.0)	12	16-13,798	13	22	
Other MSM studies	34,877	324,332	10.1 (9.0-11.2)	39	9-139,718	49	168	
Study type								*p* < 0.001
Cross-sectional	14,384	171,765	8.9 (7.7-10.1)	25	9-13,798	59	149	
Other study type	22,453	171,034	13.9 (12.2-15.7)	61	14-139,718	17	41	
Study setting								*p* = 0.325
Clinic	31,048	276,928	10.6 (9.1-12.2)	47	14-139,718	27	82	
Community or community and clinic	5,789	65,871	9.3 (7.7-11.0)	24	9-4,806	44	108	
Time period								*p* < 0.001
2000-2010	5,829	91,015	6.6 (5.4-7.9)	19	9-12,454	38	92	
2011-2022	31,008	251,784	13.6 (12.0-15.2)	47	14-139,718	39	98	
Anatomical site								*p* < 0.001
Genital	2,139	37,419	5.0 (3.6-6.5)	17	9-2,443	30	51	
Pharyngeal	326	22,394	1.9 (1.4-2.3)	22	473-12,454	3	5	
Rectal	5,893	64,243	11.8 (10.5-13.1)	30	14-10,140	29	76	
Multiple sites	28,479	218,743	13.5 (11.6-15.6)	66	14-139,718	28	58	
AXIS score classification								*p* = .387
High risk of bias	1,835	25,126	10.5 (6.9-14.6)	25	9-6,613	21	27	
Low risk of bias	35,002	317,673	9.7 (8.6-10.8)	33	13-139,718	46	163	
Sample size								*p* = .189
Less than 500	2,553	24,618	10.5 (8.7-12.3)	17	9–500	47	110	
500 or more	34,284	318,181	9.2 (7.8-10.8)	98	501-139,718	27	80	
Age^a^								*p* = .236
28 years old or younger	5,270^a^	47,411^a^	12.5 (9.6-15.7)*	26	40-13,798	25	39	
Older than 28	4,461^a^	56,266^a^	9.5 (7.8-11.4)^a^	57	25-7,094	18	41	
Total	**36,837**	**342,799**	**9.9 (8.8–11.0)**	**31**	**9-139,718**	**53**	**190**	

MSW = Men who have sex with men; TGW = Transgender women; TGW = Transgender women sex workers; AXIS = Appraisal tool for Cross-Sectional Studies (risk of bias assessment)- high risk of bias 0-10, low risk of bias 11-20; NC = not calculated.

^a^Data points for median age reported in 38% of gonorrhoea point estimates (71/188) and 41% of chlamydia point estimates (80/196).

**p-values were from univariate analysis. Note that all *I*^2^ measurements of heterogeneity were greater than 95% with one exception: pharyngeal chlamydia at 74.8%.

We created funnel plots for gonorrhoea and chlamydia using the reported point estimates to assess for publication bias. Our results suggest no evidence of publication bias.

There was strong evidence that global pooled prevalence estimates were higher between 2011 and 2022 compared to 2000 to 2010: gonorrhoea was 9.3% (95% CI: 7.7 to 11.1; 99 data points; *n* = 268,696) versus 5.0% (95% CI: 3.7 to 6.5; 89 data points; *n* = 78,557), *p* < 0.001; chlamydia was 13.6% (95% CI: 12.0 to 15.2; 95 data points; *n* = 251,784) versus 6.6% (95% CI: 5.4 to 7.9; 95 data points; *n* = 91,015), *p* < 0.001. Nearly four-fifths of all data points were from cross-sectional surveys: 78.2% for gonorrhoea and 78.4% for chlamydia. Pooled prevalence estimates from ‘other study types’, the category that contained baseline data from intervention studies and cohort studies, were significantly higher than cross-sectional studies for gonorrhoea, 11.2% (9.1–13.5); 41 data points; *n* = 160,253) versus 6.1% (95% CI: 5.0 to 7.4; 147 data points; *n* = 178,000), *p* < 0.001, and for chlamydia at 13.9% (12.2–15.7); 41 data points; *n* = 171,034) versus 8.9% (95% CI: 7.7 to 10.1; 149 data points; *n* = 171,765), *p* < 0.001. Almost half of gonorrhoea and chlamydia data points, 45.7% and 43.2%, respectively, were from clinic-based studies. We did not detect significant differences in pooled prevalence estimates between clinic-based and community or community and clinic-based studies.

Anatomical site of sample collection varied substantially. Overall, 28.2% of gonorrhoea prevalence estimates (53 of 188) were based on sampling from just one anatomical site: 69.8% (37 of 53) were from genital samples only, 22.6% (14 of 53) tested just rectal samples, and 7.5% (4 of 53) were based on pharyngeal swabs only. We found a similar pattern for the 30.5% of chlamydia point estimates (58 of 190) from one anatomical site. 63.8% (37 of 58) tested just genital samples, 29.3% (17 of 58) were based on just rectal samples, and 6.9% (4 of 58) were from the pharynx only. 38.8% (73 of 188) of gonorrhoea and 37.9% (72 of 190) of chlamydia point estimates that were from studies that sampled two anatomical sites. The most common two anatomical sites sampled were genital and rectal. Point estimates from studies undertaking the most complete testing of samples from genital, rectal, and pharyngeal sites made up 33.0% (62 of 188) and 31.6% (60 of 190) of gonorrhoea and chlamydia estimates, respectively. 67 out of these 122 point estimates from studies undertaking three-site testing were single combined data points based on testing multiple anatomical sites.

Pooled prevalence by anatomical site varied as well. Pooled prevalence estimates of gonorrhoea and chlamydia from rectal samples were higher than pooled estimates from genital samples. Rectal infection of gonorrhoea was 8.8% (95% CI: 7.4 to 10.4; 62 data points; *n* = 43,082) compared to genital and pharyngeal gonorrhoea infection 4.2% (95% CI: 2.8 to 5.8; 49 data points; *n* = 36,823) and 0.3% (95% CI: 0.0 to 1.4; 18 data points; *n* = 43,849), respectively, *p* < 0.001. Similarly, rectal infection of chlamydia, 11.8% (95% CI: 10.5 to 13.1; 76 data points; *n* = 64,243), was significantly higher than genital infection at 5.0% (95% CI: 3.6 to 6.6; 50 data points; *n* = 37,419) and pharyngeal infection with 1.9% (95% CI: 1.4 to 2.3; five data points; *n* = 22,394), *p* < 0.001.

In total, 86.0% (325 of 378) gonorrhoea and chlamydia data points were from studies that had a low risk of bias (AXIS score 11-20). The risk of bias of included point estimates by region is described in Supplementary file 3.2. Risk of study bias did not affect the pooled prevalence estimates for either infection. We found no difference in chlamydia pooled prevalence when we stratified by study sample size. Gonorrhoea pooled prevalence was lower, however, 5.4% (95% CI: 3.9–7.2) in studies with fewer than 500 MSM versus 8.6% (95% CI: 6.9–10.5) where 500 or more MSM had been tested, *p =* 0.006. We found no difference in the pooled prevalence of MSM older versus younger than the median age reported, 27 and 28, respectively, for gonorrhoea and chlamydia, although only 39.9% (151 of 378) reported the median age of study participants.

We found differences across the six SDG subgroups, the results of which are summarised in Supplementary file 5. Pooled prevalence of non-cross-sectional studies versus cross-sectional studies was significantly higher for gonorrhoea and chlamydia in sub-Saharan Africa and gonorrhoea in Europe and North America. Pooled prevalence from 2011 to 2022 versus 2000 to 2010 was higher for gonorrhoea in Central and Southern Asia, Latin America and the Caribbean and Europe and North America. Rectal prevalence was higher than other anatomical sites for gonorrhoea and chlamydia in Europe and North America and Latin America and the Caribbean, and for gonorrhoea only in Central and Southern Asia and Australia and New Zealand. The subgroup of exclusively MSM had a higher prevalence than other MSM cohorts for gonorrhoea and chlamydia in Eastern and South-eastern Asia. Clinic-based studies had a higher prevalence than the community subgroup in Central & Southern Asia, Europe and North America for gonorrhoea and in Australia and New Zealand for chlamydia.

## Discussion

### Principal findings

To the best of our knowledge, this is the first global systematic review of gonorrhoea chlamydia, and trichomoniasis prevalence among MSM. Our global pooled prevalence estimate for 2000 to 2022 for gonorrhoea was 7.2% (95% CI: 6.0–8.5) based on 188 data points and for chlamydia 9.9% (95% CI: 8.8–11.0) based on 190 data points.

Although not directly comparable, our global estimates for 2000 to 2022 for MSM for gonorrhoea are ten-times higher than the WHO global estimate for 2020 among all adult men 15–49 years of age, and four-times higher for chlamydia.^3^ Studies looking at other STIs have also documented higher prevalences in MSM than in men in general. A global systematic review of syphilis in MSM covering period 2000 to 2020 found a 14-fold increase relative to the general male population.^16^ Other studies have shown higher prevalence of *Mycoplasma genitalium* in MSM compared to the general population, particularly in MSM with symptoms of proctitis.^25,26^ Together these studies highlight the importance of increasing awareness of STIs and access to STI prevention and treatment services for MSM. Chlamydia was more prevalent than gonorrhoea among MSM at the global level and in seven of eight SDG regions. Studies of STIs in other populations of men have also reported higher prevalence of chlamydia than gonorrhoea.^4^ This may reflect a longer duration of chlamydia infection compared to gonorrhoea, and that chlamydia is more likely to be asymptomatic and so, more often untreated.^4,8,27–30^

The pooled prevalence of gonorrhoea in MSM from 2011 to 2022, 9.3%, was nearly two-times higher, than 2000 to 2010, 5.0%, a significant difference, *p* < 0.001. We observed a similarly significant difference in chlamydia with the pooled prevalence for 2011 to 2022, 13.6%, more than double than 2000 to 2010, 6.6%, *p* < 0.001. Potential explanations for these differences in prevalence over time may be increased testing attributable to increased awareness of and access to STI testing.^13,14^ 71.8%, of the 347,253 MSM tested for gonorrhoea between 2000 to 2022 were tested in 2011 or later. For chlamydia, 73.4%, of the 342,799 MSM were tested in 2011 or later. Of note, more than one-half of all MSM tested since 2011 come from one study of 139,718 asymptomatic MSM at 30 urban STI clinics across the United States between 2015 and 2019. The corrected gonorrhoea prevalence was 16.5% (95% CI: 16.3% to 16.7%) and chlamydia was 14.4% (95% CI: 14.2% to 14.6%).^31^ Differences in prevalence over time may also be an artifact of multi-site testing. In our study we found an increase in the proportion of studies reporting multi-site testing over time. We also found an increase in the pooled prevalence for both gonorrhoea and chlamydia between 2011 and 2022 when restricting the analysis to those studies that reported testing for all three anatomical sites in an overall multisite estimate. The pooled prevalence of gonorrhoea increased from 5.9% in 2000–2010 (95% CI: 3.3% to 9.2); eight data points) to 13.7% (95% CI: 12.1% to 15.4); 28 points) and for chlamydia prevalence increased from 5.7% (95% CI: 1.9% to 11.4); seven data points) to 16.0% (95% CI: 13.2% to 19.1); 27 data points. The increase in prevalence over time may also reflect changes in condom use. In some populations condom use may have fallen with increased use of pre-exposure prophylaxis for HIV.

As for other subgroups, pooled prevalence estimates of gonorrhoea and chlamydia were significantly higher in ‘other study types' that included mainly cohort and intervention studies, relative to cross-sectional studies. It is difficult to know if this difference is an artifact of participant profiles; MSM who enrol in cohort or intervention studies may be different from the general MSM population.

Rectal samples were two-times higher for both gonorrhoea and chlamydia compared to genital samples, and studies where multiple sites were sampled were three-times higher compared to genital. This is consistent with observations elsewhere.^32,33^ One-third of our studies did not specifically report collecting rectal site samples. Thus, our estimates are likely to underestimate the prevalence of gonorrhoea and chlamydia among MSM.

Our study highlights the importance of multi-site testing with aetiological assays. However, because many low-resource settings use syndromic management to diagnose and treat curable STIs as recommended by WHO,^34^ we have fewer studies from low and middle-income countries. However, estimates based only on symptomatic cases will underestimate the true prevalence.

Sub-Saharan Africa, Eastern and South-Eastern Asia and Oceania had the highest prevalence of both gonorrhoea and chlamydia globally. This high curable STI prevalence may reflect^35^ the synergistic relationship between curable STIs and the acquisition of HIV, lack of access to STI management services and barriers to access including social stigma.^36–38^

The difference in prevalence between regions may also reflect differences in behavioural and cultural practices and structural barriers to STI management. Further studies are needed to examine these relationships.

Among the nine trichomoniasis data points, the corrected prevalence estimates were all 0.0%. This is not surprising given that trichomoniasis is a relatively rare cause of urethral and rectal infection among MSM.^39^

### Strengths and limitations of the study

Our study has several strengths including a highly comprehensive search strategy that drew on four databases in an iterative fashion and included individual country names to yield data from 172 studies and 375,144 MSM. We involved two independent reviewers for rigorous study selection and data extraction processes. We limited data to studies that used aetiological assays and we corrected data points for diagnostic accuracy prior to applying random effects models to enhance the quality of our data.

Our study has several limitations. Our aim was to reduce potential selection bias by excluding studies that only exclusively tested individuals who were living with HIV or intravenous drug users, or cohorts of cases for symptomatic STIs, although we cannot be sure this fully averted a selection bias. There is the potential for underlying selection bias that stem from financial barriers, social stigma, and a lack of perceived risk that may reduce care-seeking behaviour among MSM.^40–42^ We limited our systematic review to published studies and did not search grey literature as it was beyond the scope of the project. Many studies used participant-driven sampling methods, including seed-referral which may overstate STI prevalence.^43,44^ However, nearly 80% of our point estimates were from cross-sectional surveys which had a lower pooled prevalence estimate compared to baseline data from intervention and cohort studies. Additionally, a third of studies did not report rectal testing in their prevalence estimates. Our study found that rectal prevalence was significantly higher than other anatomical sites, which is mirrored by a recent study showing increasing rectal infection over the last 5 years.^45^ This lack of complete anatomical testing could have led to underestimated infection prevalence in MSM. The definitions of the MSM population ranged between studies and over time, a reflection of growing awareness of MSM diversity and the terminology used across cultures. We are unable to assess how the evolving lexicon may have influenced our results. Despite this being a global systematic review one of the challenges we found was the limited number of data points from certain regions. Particularly, our study highlighted the lack of data from Northern Africa and Western Asia (three studies) for chlamydia and gonorrhoea, and from Oceania (four studies).

### Relation to other studies and recommendations

Our study underscores the disproportionate prevalence of curable STIs in MSM, which mirrors recent findings of a high prevalence of syphilis among MSM, as well as outbreaks of emerging and re-emerging infections such as mpox.^16,46,47^ This informs the need to provide population-specific STI prevention, which has been shown to be successful in recent settings to reduce financial barriers and stigma.^48^ Doxy-PEP and Doxy-PrEP (doxycycline post- and pre-exposure prophylaxis) for MSM may have a role, although these interventions may hasten the emergence of antimicrobial resistance. Novel interventions such as the use of meningococcal serogroup B vaccines may provide some protection against *Neisseria gonorrhoeae*. Strengthening STI surveillance among MSM, including STI prevalence and incidence tracking of STI related syndromes remains important. In parallel, antimicrobial resistance surveillance also needs to be strengthened as the emergence of untreatable gonorrhoea is a global concern.^49–51^

## Conclusions

The pooled prevalence estimate for gonorrhoea among MSM based on data from 2000 to 2022 was 7.2% (6.0–8.5; 188 data points) and for chlamydia was 9.9% (8.8–11.0; 190 data points). Action is needed to work with community groups and other stakeholders to design and implement evidence-based strategies to reduce the prevalence of gonorrohoea and chlamydia and their sequelae in MSM.

## Supplemental Material

Supplemental Material - A global meta-analysis of gonorrhoea and chlamydia prevalence among men who have sex with men from 2000 to 2022 Supplemental Material for A global meta-analysis of gonorrhoea and chlamydia prevalence among men who have sex with men from 2000 to 2022 by Ella P Davies, Motoyuki Tsuboi, Jayne Evans, Jane Rowley, Eline L Korenromp, Tim Clayton and R Matthew Chico in International Journal of STD & AIDS

## Data Availability

Additional data are available in the Supplementary files*.
