# Supplementary material for: A global meta-analysis of gonorrhoea and chlamydia prevalence among men who have sex with men from 2000 to 2022
Source: Int J STD AIDS. 2025 Apr 21;36(8):611–21. doi: 10.1177/09564624251333489 (PMC12198469; doi:10.1177/09564624251333489)
Supplement: Supplemental Material - A global meta-analysis of gonorrhoea and chlamydia prevalence among men who have sex with men from 2000 to 2022 [file sj-pdf-1-std-10.1177_09564624251333489.pdf]

Supplement to: Davies EP, Tsuboi M, Evans J, Rowley J, Korenromp EL, Clayton T, Chico RM; A global systematic review and meta-analysis of gonorrhoea, chlamydia and trichomoniasis prevalence among men who have sex with men from 2000 to 2022

Supplemental File 1 ..... **2**

1. Sample database search for study identification 2
2. Country keywords used in MEDLINE and Embase for searches strategy above. 3
3. Eligibility criteria for included studies in our meta-analsysis 3
4. Appraisal tool for cross-sectional studies (AXIS) used to assess quality of studies in our meta-analysis 4
5. Diagnostic sensitivities and specificities of assays used in included studies 4
6. Diagnostic test type and name used in included studies 5
7. Definitions of men who have sex with men amongst included studies 6

Supplemental File 2 ..... **7**

1. Gonorrhoea point prevalence data among men who have sex with men by Sustainable Development Goal region 7
2. Chlamydia point prevalence data among men who have sex with men by Sustainable Development Goal region 19
3. Trichomoniasis point prevalence data among men who have sex with men by Sustainable Development Goal region 31

Supplemental File 3 ..... **32**

1. Number of data points included in the meta-analysis by study year and SDG region 32
2. Summary of the appraisal tool for cross-sectional studies (AXIS) score of the data points included in the meta-analysis by Sustainable Development Goal region. 32
3. Funnel plots 34
   3. a) Funnel plots to assess publication bias within included gonorrheoa studies 34
   3. b) Funnel plots to assess publication bias within included chlamydia studies 36

Supplemental File 4 ..... **38**

1. Gonorrhoea pooled prevalence estimates: global and Sustainable Development Goal regions 38
   1.a. Gonorrhoea global pooled prevalence estimates stratified by Sustainable Development Goal region 38
   1.b. Sub-Saharan Africa gonorrhoea pooled prevalence estimates 39
   1.c. Northern Africa and Western Asia gonorrhoea pooled prevalence estimates 40
   1.d. Central and Southern Asia gonorrhoea pooled prevalence estimates 41
   1.e. Eastern and South-Eastern Asia gonorrhoea pooled prevalence estimates 42
   1.f. Latin America and the Caribbean gonorrhoea pooled prevalence estimates 43
   1.g. Australia and New Zealand gonorrhoea pooled prevalence estimates 44
   1.h. Oceania gonorrhoea pooled prevalence estimates 45
   1.i. Europe and North America gonorrhoea pooled prevalence estimates 46
2. Chlamydia global and SDG regions 47
   2.a. Chlamydia pooled prevalence estimates: global and Sustainable Development Goal regions 47
   2.b. Sub-Saharan Africa chlamydia pooled prevalence estimates 48
   2.c. Northern Africa and Western Asia chlamydia pooled prevalence estimates 49
   2.d. Central and Southern Asia chlamydia pooled prevalence estimates 50
   2.e. Eastern and South-Eastern Asia chlamydia pooled prevalence estimates 51
   2.f. Latin America and the Caribbean chlamydia pooled prevalence estimates 52

2.g. Australia and New Zealand chlamydia pooled prevalence estimates 53
2.h. Oceania chlamydia pooled prevalence estimates 54
2.i. Europe and North America chlamydia pooled prevalence estimates 55

Supplemental File 5 56

1. Gonorrhoea and chlamydia: Subgroup analysis of prevalence among men who have sex with men point estimates included in the meta-analysis 56

Supplemental File 6 57

1. PRISMA 2020 checklist 57

References 62

Supplemental File 1

1. Sample database search for study identification

Note: Panama is used below for illustration purposes

Database	Domain	MeSH Terms	Full-text searching terms
MEDLINE	1: STIs	1a	1b
		Chlamydia OR Chlamydia trachomatis OR Gonorrhea OR *Neisseria gonorrhoeae OR Trichomonas OR Trichomonas vaginitis* OR *Trichomonas vaginalis*	*Chlamydia trachomatis* OR Chlamydia* OR CT OR *Neisseria gonorrhoeae* OR Gonorrhoea OR Gonorrhea OR Gonococc* OR NG OR *Trichomonas vaginalis* OR Trichomoniasis OR TV
	2: MSM	2a	2b
		Sexual and gender minorities OR Homosexuality, male OR Bisexuality	MSM OR Men who have sex with men OR Gay* OR Bisexual* OR Homosexual* OR Transgender* OR High-risk population OR Same sex
	3: Country name	3a	3b
		Panama	Panama$^{\Delta}$
Summation: (1a OR 1b) AND (2a OR 2b) AND (3a OR 3b)			
Embase	1: STIs	1a	1b
		Chlamydia OR *Chlamydia trachomatis* OR Gonorrhea OR Trichomoniasis OR *Trichomonas vaginalis*	*Chlamydia trachomatis* OR Chlamydia* OR CT OR *Neisseria gonorrhoeae* OR Gonorrhoea OR Gonorrhea OR Gonococc* OR NG OR *Trichomonas vaginalis* OR Trichomoniasis OR TV
	2: MSM	2a	2b
		Men who have sex with men OR Homosexual, male OR Bisexual, male OR High-risk population OR “men who have sex with men and women” OR Transgender	MSM OR Men who have sex with men OR Gay* OR Bisexual* OR Homosexual* OR Transgender* OR High-risk population OR Same sex
	3: Country name	3a	3b
		Panama	Panama
Summation: (1a OR 1b) AND (2a OR 2b) AND (3a OR 3b)			
AIM	1: STIs	Chlamydia OR Gonorrhoea OR Gonorrhea OR Trichomonas	
LILACS	1: STIs	1a	
		Chlamydia trachomatis OR Chlamydia* OR CT OR *Neisseria gonorrhoeae* OR Gonorrhoea OR Gonorrhea OR Gonococc* OR NG OR *Trichomonas vaginalis* OR Trichomoniasis OR TV	
	2: MSM	2a	
		MSM OR Men who have sex with men OR Gay* OR Bisexual* OR Homosexual* OR Transgender* OR High-risk population OR Same sex	
Summation: (1a) AND (2a)			

2. Country keywords used in MEDLINE and Embase for searches strategy above.

SDG region	Country-keyword search terms
**Sub-Saharan Africa**	Angola, Benin, Botswana, Burkina Faso, Burundi, Cabo Verde (+Cape Verde), Cameroon, Central African Republic, Chad, Comoros, Congo, Cote d'Ivoire (+Ivory Coast), Democratic Republic of the Congo (+DRC), Djibouti, Equatorial Guinea, Eritrea, Ethiopia, Gabon, Gambia, Ghana, Guinea, Guinea-Bissau, Kenya, Lesotho, Liberia, Madagascar, Malawi, Mali, Mauritania, Mauritius, Mayotte, Mozambique, Namibia, Niger, Nigeria, Reunion, Rwanda, "Sao Tome and Principe," Senegal, Seychelles, Sierra Leone, Somalia, South Africa, South Sudan, Eswatini (+Swaziland), Togo, Uganda, United Republic of Tanzania, Zambia, Zimbabwe
**Central & Southern Asia**	Kazakhstan, Kyrgyzstan, Tajikistan, Uzbekistan, Afghanistan, Bangladesh, Bhutan, India, Iran (+Islamic Republic of Iran), Maldives, Nepal, Pakistan, Sri Lanka
**Eastern & South-Eastern Asia**	China, China Hong Kong SAR, China Macao SAR, Macao, Democratic People's Republic of Korea (+ North Korea, South Korea), Japan, Mongolia, Republic of Korea, Brunei Darussalam (+Brunei), Cambodia, Indonesia, Lao People's Democratic Republic (+Laos), Malaysia, Myanmar, Philippines, Singapore, Thailand, Timor-Leste, Vietnam (+Vietnam)
**Oceania, Australia & NZ**	Fiji, New Caledonia, Papua New Guinea, Solomon Islands, Vanuatu, Kiribati, Marshall Islands, Federated States of Micronesia, Palau, Guam, French Polynesia, Wallis and Futuna Island, Pitcairn, Cook Islands, Niue, Tokelau, Tonga Tuvalu, American Samoa, Samoa; Australia, Christmas Island, Cocos Island, Heard Island, McDonald Islands, Norfolk Island, New Zealand
**Europe & North America**	Bermuda, Canada, Greenland, USA (searched separately as 'United States' & 'America'), Bulgaria, Belarus, Czech Republic, Hungary, Moldova, Poland, Romania, Russia (+'Russian Federation'), Slovakia, Ukraine, Aland islands, Channel islands, Denmark, Estonia, Faroe Islands, Finland, Isle of man, UK (searched also as 'United Kingdom', 'Northern Ireland', 'Britain'), Iceland, Latvia, Lithuania, Norway, Sweden, Albania, Andorra, "Bosnia and Herzegovina," Croatia, Greece, Italy, Malta, Montenegro<br>Portugal, San Marino, Serbia, Slovenia, Spain, Macedonia, Austria, Belgium, Switzerland, Germany, France, Liechtenstein, Luxembourg, Monaco, Netherlands (+ Holland)
**Northern Africa & Western Asia**	Algeria, Egypt, Libya (+Libyan Arab Jamahiriya), Morocco, Sudan, Tunisia, Western Sahara, Azerbaijan, Armenia, Bahrain, Cyprus, Georgia (+Georgia (Republic)), Iraq, Israel, Jordan, Kuwait, Lebanon, State of Palestine (Palestine), Oman, Qatar, Saudi Arabia, Syrian Arab Republic (+Syria), Turkey, United Arab Emirates, Yemen
**Latin America & Caribbean**	Anguilla, "Antigua and Barbuda", Aruba, Bahamas, Barbados, Bonaire, Sint Eustatius, Saba, (+Caribbean Netherlands), British Virgin Islands, Cayman islands, Cuba, Curacao, Dominica, Dominican Republic, Grenada, Guadeloupe, Haiti, Jamaica, Martinique, Montserrat, Puerto Rico, "Saint Kitts and Nevis", Saint Lucia, "Saint Vincent and the Grenadines", Sint Maarten (+Saint Martin), Suriname, "Trinidad and Tobago", "Turks and Caicos Islands", United States Virgin Islands (+Virgin Islands), Honduras, Costa Rica, El Salvador, Guatemala, Mexico, Nicaragua, Panama, Argentina, Belize, Bolivia (+Plurinational State of Bolivia), Brazil, Chile, Colombia, Ecuador, French Guiana, Falkland Islands (+Malvinas), "South Georgia and the South Sandwich Islands", Guyana, Paraguay, Peru, Uruguay, Venezuela (+Bolivian Republic of Venezuela)

3. Eligibility criteria for included studies in our meta-analsysis

Category	**Inclusion criteria**	**Exclusion criteria**
Geography	All	None
Outcome	Prevalence statistics on *Chlamydia Trachomatis, Neisseria Gonorrhoea* or *Trichomonas Vaginalis*	-
Population	Men who have sex with men (MSM) defined as any person identifying as a man reporting any form of sexual intercourse with another person identifying as a man, including those identifying as homosexual, bisexual, heterosexual, transgender and men selling sex populations	All other populations, exclusively HIV positive MSM cohorts, exclusively injecting drug users and exclusively symptomatic MSM cohorts*
Intervention	At least one biological diagnostic assay	Clinical, self-reported or syndromic management in absence of biological test. Serology only tests.
Study design	Cross-sectional/cohort/case-control study, routine data and any other quantitative study with primary data	Qualitative studies, review articles, editorials, policy papers and modelling studies using prediction models
Study period	1 January 2000 to 19 April 2022	Prior to January 2000

*dysuria, urethral discharge and sores in the genital area are all commonly reported and sometimes rectal discharge is present in rectal infection.

4. Appraisal tool for cross-sectional studies (AXIS) used to assess quality of studies in our meta-analysis

1	Were the aims/objectives of the study clear?			
Methods				
2	Was the study design appropriate for the stated aim(s)?			
3	Was the sample size justified?			
4	Was the target/reference population clearly defined? (Is it clear who the research was about?)			
5	Was the sample frame taken from an appropriate population base so that it closely represented the target/reference population under investigation?			
6	Was the selection process likely to select subjects/participants that were representative of the target/reference population under investigation?			
7	Were measures undertaken to address and categorise non-responders?			
8	Were the risk factor and outcome variables measured appropriate to the aims of the study?			
9	Were the risk factor and outcome variables measured correctly using instruments/measurements that had been trialled, piloted or published previously?			
10	Is it clear what was used to determined statistical significance and/or precision estimates? (e.g. p-values, confidence intervals)			
11	Were the methods (including statistical methods) sufficiently described to enable them to be repeated?			
Results				
12	Were the basic data adequately described?			
13	Does the response rate raise concerns about non-response bias?			
14	If appropriate, was information about non-responders described?			
15	Were the results internally consistent?			
16	Were the results presented for all the analyses described in the methods?			
Discussion				
17	Were the authors' discussions and conclusions justified by the results?			
18	Were the limitations of the study discussed?			
Other				
19	Were there any funding sources or conflicts of interest that may affect the authors' interpretation of the results?			
20	Was ethical approval or consent of participants attained?			

5. Diagnostic sensitivities and specificities of assays used in included studies

Infection	Diagnostic assay	Sensitivity (%)	Specificity (%)
Neisseria gonorrhoeae	Culture and microscopy[1]	81, 100*	100
	PCR with urine sample[2]	90.4	99.7
	PCR with urethral swab[2]	96.1	99.0
	PCR with rectal swab (physician or self-collected)[3]	78.4, 84.3*	99.8, 100*
	PCR with oropharyngeal swab[3]	80.3	73.0
	LCR[1]	95, 100*	98, 100*
	SDA with urine sample[4]	97.4	99.5
	SDA with urethral swab[4]	99.1	99.7
	SDA with rectal swab[5]	77.8	100
	SDA with oropharyngeal swab[5]	75	99.5
Chlamydia trachomatis	Culture and microscopy[1]	74, 90*	98, 99*
	ELISA, EIA[1]	71, 97*	97, 99*
	PCR with urine sample[2]	84.0	99.3
	PCR with urethral swab[2]	87.5	99.2
	PCR with rectal swab (physician or self-collected)[3]	71.2, 81.8*	99.6, 100*
	PCR with oropharyngeal swab[6]	86, 100*	100
	LCR[1]	90, 97*	99, 100*
	SDA with urine sample[2]	93.1	93.8
	SDA with urethral swab[2]	92.4	96.3
	SDA with rectal swab[5]	76.5	100
	SDA with oropharyngeal swab[5]	33.3	100
Trichomonas vaginalis	Wet mount microscopy[1]	38, 82*	100
	In-Pouch culture[7]	96	100
	PCR with urine[8]	100	88
PCR = Polymerase chain reaction; LCR = Ligase chain reaction; SDA = Strand displacement amplification; ELISA, EIA = Enzyme immunoassay<br>*where a range was given, the midpoint was used			

The equation we used for correction for diagnostic error is:
Corrected prevalence = (Observed prevalence + Specificity -1)/(Sensitivity +specificity –1).

6. Diagnostic test type and name used in included studies

Diagnostic test type	Diagnostic test name
PCR	NAAT-AC2, NAAT-Cobas Amplicor, Hologic Aptima Assay, AC2 NAAT, abbot RealTime PCR, GeneXpert CT/NG, Cobas Amplicor CT/NG, Allplex STI essential Assay, Taqman probe PCR, PCR Digene Hybrid Capture, Gene-Probe Aptima TMA, FTD Urethritis Basic Kit (FastTrack), Versant CT/GC DNA Assay, Amplipram NCMT (PCR-RT)
SDA	BD ProbeTec SDA
PCR = Polymerase chain reaction; LCR = Ligase chain reaction; SDA = Strand displacement amplification, NAAT = Nucleic Acid Amplification Test, CT/NG = *Chlamydia trachomatis* and *Neisseria gonorrhoeae,* RT = reverse transcriptase	

7. Definitions of men who have sex with men amongst included studies

Definition	Infection			
	Gonorrhoea		Chlamydia	
	No. of studies	No. of MSM tested	No. of studies	No. of MSM tested
MSN – Self-identifying as gay, bisexual or MSM or no clear definition other than MSM	66	225,982	74	222,760
MSM – Sexual intercourse ever	10	14,112	12	12,175
MSM – Sexual intercourse < 5 years	1	1,419	2	2,625
MSM – Sexual intercourse < 12 months	35	75,259	25	74,929
MSM – Sexual intercourse < 6 months	18	18,815	19	19,057
MSM – Unprotected sexual intercourse < 6 months	4	2,177	3	1,613
MSM – Unprotected sexual intercourse < 3 months	5	2,476	5	2,113
MSM – Sexual intercourse ‘regularly’	2	705	2	1,110
MSM – Multiple partners < 6 months or sex with a partner with HIV	1	200	1	200
MSM – Anal sex with > 4 different partners or any history of transactional sex or sex with a partner with HIV or with unknown HIV status	1	608	1	608
MSM – Men who have only ever had sex with men	2	808	2	808
MSM – Active members of the gay community	1	106	1	106
MSM – Men who have sex with men for money or equivalent transaction	4	587	4	618
MSM – Transgender women (TGW) who have sex with men or TGW	2	1,079	2	1,079
MSM – TGW who have sex with men or TGW for money or equivalent transaction	1	186	2	266
MSM – Oral sex < 2 weeks	1	473	1	471
MSM – Black or afro-Caribbean men self-identifying as gay or bisexual or MSM	2	2,121	3	2,121
MSM – Sexual intercourse < 12 months and oral sex < 2 months	1	140	1	140
**Total**	**157**	**347,253**	**159**	**342,799**

*Most studies did not specify the inclusion or exclusion of TGW unless otherwise stated. Sexual intercourse was defined as oral or anal sex unless otherwise stated.

Supplemental File 2

1. Gonorrhoea point prevalence data among men who have sex with men by Sustainable Development Goal region

Reference	Country	Year(s)	Diagnostic method	No. of MSM		Anatomical sample site	Uncorrected prevalence (%)	95% CI	Corrected prevalence (%)*	95% CI	Axis score
				Positive	Tested						
**Sub-Saharan Africa**											
**Sanders 2010[9]**	**Kenya**	**NR**	**NAAT-AC2**	**4**	**43**	**Rectal**	**9.0**	**3.7, 21.6**	**10.9**	**4.7, 23.7**	**9**
Sanders 2010[9]	Kenya	NR	NAAT-AC2	1	43	Genital	2.0	0.4, 12.1	1.9	0.3, 11.0	9
**Wade 2010[10]**	**Senegal**	**2004**	**NAAT-AC2**	**23**	**419**	**Genital**	**5.5**	**3.7, 8.1**	**5.8**	**3.9, 8.4**	**12**
**Wade 2010[10]**	**Senegal**	**2007**	**NAAT-AC2**	**13**	**500**	**Genital**	**2.6**	**1.5, 4.4**	**2.6**	**1.5, 4.3**	**12**
**Vuylsteke 2012[11]**	**Cote d'Ivoire**	**2007-08**	**NAAT-AC2**	**12**	**94**	**Multisite**	**12.8**	**7.5, 21.0**	**13.9**	**8.3, 22.3**	**12**
Vuylsteke 2012[11]	Cote d'Ivoire	2007-08	NAAT-AC2	4	94	Genital	4.3	1.7, 10.4	5.5	2.4, 12.2	12
Vuylsteke 2012[11]	Cote d'Ivoire	2007-08	NAAT-AC2	7	94	Rectal	7.4	3.7, 14.6	10.3	5.7, 18.1	12
**Kim 2015[12]**	**Uganda**	**2008**	**NAAT-AC2**	**5**	**286**	**Rectal**	**1.8**	**0.8, 4.0**	**2.1**	**1.0, 4.5**	**14**
Kim 2015[12]	Uganda	2008	NAAT-AC2	4	288	Genital	1.4	0.5, 3.5	1.2	0.5, 3.3	12
**Muraguri 2015[13]**	**Kenya**	**2010**	**NAAT-Roche Amplicor**	**20**	**290**	**Multisite**	**6.9**	**4.5, 10.4**	**7.3**	**4.9, 10.9**	**13**
Muraguri 2015[13]	Kenya	2010	NAAT-Roche Amplicor	13	290	Rectal	4.5	2.6, 7.5	5.4	3.4, 8.7	13
Muraguri 2015[13]	Kenya	2010	NAAT-Roche Amplicor	10	290	Genital	3.5	1.9, 6.2	3.6	1.96, 6.36	13
**Sanders 2014[14]**	**Kenya**	**2011**	**NAAT-AC2**	**14**	**244**	**Rectal**	**5.7**	**3.4, 9.4**	**6.9**	**4.4, 10.8**	**11**
Sanders 2014[14]	Kenya	2011	NAAT-AC2	4	244	Genital	1.6	0.6, 4.1	0.6	0.1, 2.6	11
**Rebe 2015[15]**	**South Africa**	**2012**	**NAAT-AC2**	**21**	**200**	**Multisite**	**10.5**	**5.4, 13.2**	**10.3**	**6.8, 15.3**	**10**
Rebe K, 2015[15]	South Africa	2012	NAAT-AC2	15	200	Pharyngeal	7.5	4.6, 12.0	0.0	0.0, 1.9	10
Rebe K, 2015[15]	South Africa	2012	NAAT-AC2	17	200	Rectal	8.5	5.4, 13.2	10.3	6.8, 15.3	10
Rebe K, 2015[15]	South Africa	2012	NAAT-AC2	7	200	Genital	3.5	1.7, 7.0	3.6	1.7, 7.1	10
**Ross 2014[16]**	**UR Tanzania[∇]**	**2012**	**NAAT-AC2**	**1**	**40**	**Rectal**	**2.5**	**0.4, 12.9**	**3.0**	**0.6, 13.6**	**11**
**Ross 2014[16]**	**UR Tanzania[∇]**	**2012**	**NAAT-AC2**	**26**	**180**	**Rectal**	**14.4**	**10.1, 20.3**	**17.6**	**12.7, 23.8**	**11**
Ross 2014[16]	UR Tanzania	2012	NAAT-AC2	1	40	Genital	2.5	0.4, 12.9	2.4	0.4, 12.8	11
Ross 2014[16]	UR Tanzania	2012	NAAT-AC2	1	179	Genital	0.6	0.1, 3.1	0.3	0.0, 2.7	11
**Tafuma 2014[17]**	**Botswana[∇]**	**2012**	**PCR**	**3**	**145**	**Rectal**	**2.1**	**0.7, 5.9**	**2.5**	**0.9, 6.5**	**11**
**Tafuma 2014[17]**	**Botswana[∇]**	**2012**	**PCR**	**5**	**275**	**Rectal**	**1.9**	**0.8, 4.2**	**2.2**	**1.0, 4.7**	**11**
Tafuma 2014[17]	Botswana	2012	PCR	3	145	Genital	1.9	0.7, 5.9	1.8	0.6, 5.5	11
Tafuma 2014[17]	Botswana	2012	PCR	4	275	Genital	1.6	0.6, 3.7	1.4	0.6, 3.7	11
**Venter, 2019[18]**	**South Africa**	**2011-2012**	**PCR**	**17**	**199**	**Rectal**	**8.5**	**5.4, 13.3**	**10.4**	**6.9, 15.4**	**12**
Venter, 2019[18]	South Africa	2011-2012	PCR	7	200	Genital	3.5	1.7, 7.0	3.6	1.7, 7.1	12
Venter, 2019[18]	South Africa	2011-2012	PCR	12	194	Pharyngeal	6.2	3.6, 10.5	0.0	0.0, 1.9	12
**Kayode, 2020[19]**	**Nigeria**	**2013**	**PCR**	**304**	**1,447**	**Multisite**	**21.0**	**19.0, 23.2**	**23.2**	**21.1, 25.4**	**12**
**Keshinro 2016[20]**	**Nigeria[∇]**	**2013-16**	**NAAT-AC2**	**114**	**546**	**Multisite**	**20.9**	**17.7, 24.5**	**22.9**	**19.5, 26.6**	**14**
**Keshinro 2016[20]**	**Nigeria [∇]**	**2013-16**	**NAAT-AC2**	**95**	**316**	**Multisite**	**30.1**	**25.3, 35.3**	**33.1**	**28.1, 38.4**	**14**

1. Gonorrhoea point prevalence data (continued)

Reference	Country	Year(s)	Diagnostic method	No. of MSM		Anatomical sample site	Uncorrected prevalence (%)	95% CI	Corrected prevalence (%)*	95% CI	Axis score
				Positive	Tested						
Keshinro 2016[20]	Nigeria	2013-16	NAAT-AC2	14	316	Genital	4.4	2.7, 7.3	4.6	2.8, 7.4	14
Keshinro 2016[20]	Nigeria	2013-16	NAAT-AC2	93	316	Rectal	29.4	24.7, 34.7	36.1	31.0, 41.5	14
Keshinro 2016[20]	Nigeria	2013-16	NAAT-AC2	19	546	Genital	3.5	2.2, 5.4	3.6	2.3, 5.5	14
Keshinro 2016[20]	Nigeria	2013-16	NAAT-AC2	108	546	Rectal	19.8	16.6, 23.3	24.3	20.8, 28.0	14
**De Baetelier, 2020[21]**	**B. Faso, Togo, Mali, CDI****	**2016**	**Abbott RealTime PCR**	**57**	**497**	**Multisite**	**11.5**	**9.0, 14.6**	**12.4**	**9.8, 15.6**	**9**
**Jones 2020[22]**	**South Africa**	**2015-2016**	**GeneXpert CT/NG**	**30**	**189**	**Rectal**	**15.9**	**11.4, 21.8**	**19.6**	**14.6, 25.8**	**11**
Jones 2020[22]	South Africa	2015-2016	GeneXpert CT/NG	8	288	Genital	2.8	1.4, 5.4	3.0	1.6, 5.7	11
**Ngetsa 2020[23]**	**Kenya**	**2016-2017**	**GeneXpert CT/NG**	**10**	**104**	**Rectal**	**9.6**	**5.3, 16.8**	**11.7**	**6.8, 19.3**	**16**
**Otieno 2020[24]**	**Kenya**	**2015-2016**	**Cobas Amplicor CT/NG**	**25**	**619**	**Rectal**	**4.0**	**2.8, 5.9**	**4.8**	**3.4, 6.8**	**19**
Otieno 2020[24]	Kenya	2015-2016	Cobas Amplicor CT/NG	23	619	Rectal	3.7	2.5, 5.5	3.4	2.3, 5.2	19
**Ferre 2019[25]**	**Togo**	**2017**	**Allplex STI Essential Assay**	**24**	**207**	**Rectal**	**11.6**	**7.9, 16.7**	**14.2**	**10.1, 19.6**	**12**
**Twahirawa 2021[26]**	**Rwanda**	**2018**	**GeneXpert CT/NG**	**65**	**737**	**Multisite**	**8.8**	**7.0, 11.1**	**9.5**	**77.5, 11.8**	**11**
**Northern Africa and Western Asia**											
**Mor 2012[27]**	**Israel**	**2002-08**	**PCR**	**20**	**1,064**	**Genital**	**1.85**	**1.2, 2.9**	**1.7**	**1.1, 2.7**	**10**
**Hancali 2019[28]**	**Morocco**	**2017**	**GeneXpert CT/NG**	**20**	**238**	**Rectal**	**8.4**	**5.5, 12.6**	**10.2**	**7.0, 14.7**	**8**
**Zucker 2022[29]**	**Israel**	**2019-2020**	**GeneXpert CT/NG**	**29**	**210**	**Rectal**	**11.0**	**7.4, 15.9**	**0.0**	**0.0, 1.8**	**13**
Zucker 2022[29]	Israel	2019-2020	GeneXpert CT/NG	0	210	Genital	0	0.0, 1.8	0.0	0.0, 1.8	13
Zucker 2022[29]	Israel	2019-2020	GeneXpert CT/NG	22	210	Pharyngeal	10.5	7.0, 15.4	12.8	8.9, 18.0	13
**Central and Southern Asia**											
**Rehan 2009[λ30]**	**India[∇]**	**2004**	**PCR**	**13**	**395**	**Genital**	**3.3**	**1.9, 5.6**	**2.4**	**1.3 4.5**	**13**
**Rehan 2009[λ30]**	**India[∇]**	**2004**	**PCR**	**69**	**395**	**Rectal**	**17.5**	**14.0, 21.5**	**21.4**	**17.7, 25.7**	**13**
Rehan 2009[λ30]	India	2004	PCR	23	396	Genital	5.8	3.9, 8.6	5.05	3.3, 7.7	13
Rehan 2009[λ30]	India	2004	PCR	0	400	Rectal	0.0	0.0, 1.0	0.0	0.0, 1.0	13
**Brahmam 2008[31]**	**India[∇]**	**2006-07**	**NAAT**	**2**	**298**	**Genital**	**0.6**	**0.2, 2.4**	**0.3**	**0.1, 1.9**	**14**
**Brahmam 2008[31]**	**India[∇]**	**2006-07**	**NAAT**	**10**	**1,621**	**Genital**	**0.6**	**0.3, 1.1**	**0.3**	**0.1, 0.8**	**14**
**Brahmam 2008[31]**	**India[∇]**	**2006-07**	**NAAT**	**2**	**653**	**Genital**	**0.3**	**0.1, 1.1**	**0.0**	**0.0, 0.6**	**14**
**Brahmam 2008[31]**	**India[∇]**	**2006-07**	**NAAT**	**2**	**2025**	**Genital**	**0.1**	**0.0, 0.4**	**0.0**	**0.0, 0.2**	**14**
**Hawkes 2009[λ32]**	**Pakistan[∇]**	**2007**	**NAAT-Roche Amplicor PCR**	**42**	**559**	**Rectal**	**7.5**	**5.6, 10.0**	**9.1**	**7.0, 11.8**	**12**
**Hawkes 2009[δ32]**	**Pakistan[∇]**	**2007**	**NAAT-Roche Amplicor PCR**	**1**	**16**	**Rectal**	**6.3**	**1.1, 28.3**	**7.6**	**1.6, 30.1**	**12**
**Hawkes 2009[λ32]**	**Pakistan[∇]**	**2007**	**NAAT-Roche Amplicor PCR**	**9**	**87**	**Rectal**	**10.3**	**5.5, 18.5**	**12.6**	**7.1, 21.1**	**12**
**Hawkes 2009[δ32]**	**Pakistan[∇]**	**2007**	**NAAT-Roche Amplicor PCR**	**51**	**253**	**Rectal**	**20.2**	**15.7, 25.5**	**24.8**	**19.8, 30.4**	**12**
Hawkes 2009[δ32]	Pakistan	2007	NAAT-Roche Amplicor PCR	0	16	Genital	0.0	0.0, 19.4	0.0	0.0, 19.4	12
Hawkes 2009[λ32]	Pakistan	2007	NAAT-Roche Amplicor PCR	0	559	Genital	0.0	0.0, 0.7	0.0	0.0, 0.7	12
Hawkes 2009[λ32]	Pakistan	2007	NAAT-Roche Amplicor PCR	0	87	Genital	0.0	0.0, 4.2	0.0	0.0, 4.2	12
Hawkes 2009[δ32]	Pakistan	2007	NAAT-Roche Amplicor PCR	0	253	Genital	0.0	0.0, 1.5	0.0	0.0, 1.5	12

1. Gonorrhoea point prevalence data (continued)

Reference	Country	Year(s)	Diagnostic method	No. of MSM		Anatomical sample site	Uncorrected prevalence (%)	95% CI	Corrected prevalence (%)*	95% CI	Axis score
				Positive	Tested						
**Vandana 2013[33]**	**India**	**2008-09**	**GenProbe APTIMA-NAAT**	**20**	**183**	**Rectal**	**10.9**	**7.2, 16.3**	**13.3**	**9.1, 19.0**	**11**
Vandana 2013[33]	India	2008-09	GenProbe APTIMA-NAAT	2	183	Pharyngeal	1.1	0.3, 3.9	0.0	0.0, 2.1	11
**Chunchanur, 2019[34]**	**India**	**2015**	**RealTime PCR**	**20**	**23**	**Urethral**	**87.0**	**67.9, 95.5**	**90.4**	**72.1, 97.2**	**4**
Chunchanur, 2019[34]	India	2015	RealTime PCR	10	14	Pharyngeal	71.4	45.4, 88.3	83.4	57.5, 94.9	4
**Safren 2020[35]**	**India**	**2016**	**Abbott RealTime PCR**	**73**	**608**	**Multisite**	**12.0**	**9.7, 14.8**	**13.1**	**10.6, 16.0**	**17**
**Eastern and South-Eastern Asia**											
**Chau 2016[36]**	**Hong Kong SAR**	**NR**	**AC2 NAAT and culture**	**10**	**158**	**Rectal**	**6.3**	**3.5, 11.3**	**7.6**	**4.4, 12.9**	**11**
Chau 2016[36]	Hong Kong SAR	NR	AC2 NAAT and culture	5	158	Pharyngeal	3.2	1.4, 7.2	3.54	1.6, 7.7	11
Chau 2016[36]	Hong Kong SAR	NR	AC2 NAAT and culture	0	158	Genital	0.0	0.0, 2.4	0.0	0.0, 2.4	11
**Wei 2009[37]**	**China**	**1999-2000**	**LCR**	**0**	**38**	**Genital**	**0.0**	**0.0, 9.2**	**0.0**	**0.0, 9.2**	**12**
**Girault 2004[38]**	**Cambodia**	**2000**	**NAAT-cobas amplicor**	**1**	**206**	**Rectal**	**0.3**	**0.1, 2.7**	**0.2**	**0.0, 2.3**	**14**
**Jiang 2006[39]**	**China**	**2003**	**NAAT-Roche Amplicor**	**3**	**122**	**Genital**	**2.7**	**0.8, 7.0**	**1.8**	**0.5, 6.0**	**11**
**Hoang 2006[40]**	**Vietnam[∇]**	**2005-06**	**PCR**	**46**	**397**	**Rectal**	**11.5**	**8.8, 15.1**	**14.0**	**11.0, 17.8**	**15**
**Hoang 2006[40]**	**Vietnam[∇]**	**2005-06**	**PCR**	**26**	**393**	**Genital**	**6.7**	**4.6, 9.5**	**8.1**	**5.8, 11.3**	**15**
Hoang 2006[40]	Vietnam	2005-06	PCR	12	397	Genital	3.1	1.7, 5.2	3.11	1.8, 5.3	15
Hoang 2006[40]	Vietnam	2005-06	PCR	26	393	Genital	6.7	4.6, 9.5	8.13	5.8, 11.3	15
**Pattanasin 2018[41]**	**Thailand**	**2006-10**	**NAAT-Roche Amplicor**	**109**	**1,695**	**Multisite**	**6.4**	**5.4, 7.7**	**6.8**	**5.7, 8.1**	**14**
Pattanasin 2018[41]	Thailand	2006-10	NAAT-Roche Amplicor	12	1,695	Genital	0.7	0.4, 1.2	0.44	0.2, 0.9	14
Pattanasin 2018[41]	Thailand	2006-10	NAAT-Roche Amplicor	7	1,689	Pharyngeal	0.4	0.2, 0.9	0.44	0.2, 0.9	14
Pattanasin 2018[41]	Thailand	2006-10	NAAT-Roche Amplicor	90	1,552	Rectal	5.8	4.7, 7.1	7.02	5.9, 8.4	14
**Tongtoyai 2015[42]**	**Thailand**	**2006-10**	**NAAT-Roche Amplicor**	**98**	**1,596**	**Rectal**	**6.1**	**5.1, 7.4**	**7.4**	**6.2, 8.8**	**14**
Tongtoyai 2015[42]	Thailand	2006-10	NAAT-Roche Amplicor	32	1,743	Genital	1.8	1.3, 2.6	1.66	1.2, 2.4	14
Tongtoyai 2015[42]	Thailand	2006-10	NAAT-Roche Amplicor	8	1,743	Pharyngeal	0.5	0.2, 0.9	0.55	0.3, 1.0	14
**Guo 2013[43]**	**China**	**2007**	**Chocolate agar culture**	**22**	**139**	**Genital**	**15.8**	**10.7, 22.8**	**17.5**	**12.0, 24.6**	**10**
**Morineau 2011[44]**	**Indonesia[∇]**	**2007**	**NAAT-Cobas amplicor**	**55**	**247**	**Rectal**	**22.3**	**17.5, 27.9**	**27.3**	**22.2, 33.2**	**12**
**Morineau 2011[44]**	**Indonesia[∇]**	**2007**	**NAAT-Cobas amplicor**	**45**	**242**	**Rectal**	**18.6**	**14.2, 24.0**	**22.8**	**17.9, 28.5**	**12**
**Morineau 2011[44]**	**Indonesia[∇]**	**2007**	**NAAT-Cobas amplicor**	**37**	**249**	**Rectal**	**14.9**	**11.0, 19.8**	**18.2**	**13.9, 23.5**	**12**
Morineau 2011[44]	Indonesia	2007	NAAT-Cobas amplicor	5	250	Genital	2.0	0.9, 4.6	2.3	1.1, 5.1	12
Morineau 2011[44]	Indonesia	2007	NAAT-Cobas amplicor	6	249	Genital	2.4	1.1, 5.2	1.0	0.3, 3.2	12
Morineau 2011[44]	Indonesia	2007	NAAT-Cobas amplicor	3	250	Genital	1.2	0.4, 3.5	1.9	0.8, 4.4	12
**Prabawanti 2011[λ,45]**	**Indonesia**	**2007**	**NAAT-Roche Amplicor**	**213**	**734**	**Rectal**	**29.0**	**25.9, 32.4**	**35.6**	**32.2, 39.1**	**10**
**Jung 2012[46]**	**Rep of Korea**	**2008**	**PCR**	**0**	**106**	**Pharyngeal**	**0.0**	**0.0, 3.5**	**0.0**	**0.0, 3.5**	**10**
Jung 2012[46]	Rep of Korea	2008	PCR	0	106	Genital	0.0	0.0, 3.5	0.0	0.0, 3.5	10
**Fu 2015[47]**	**China**	**2009**	**NAAT-Roche Amplicor**	**15**	**413**	**Genital**	**3.6**	**2.2, 5.9**	**3.7**	**2.3, 6.0**	**10**
**Pham 2012[48]**	**Vietnam**	**2009**	**NAAT-Roche Amplicor**	**7**	**381**	**Genital**	**1.8**	**0.9, 3.7**	**1.7**	**0.8, 3.5**	**12**

1. Gonorrhoea point prevalence data (continued)

Reference	Country	Year(s)	Diagnostic method	No. of MSM		Anatomical sample site	Uncorrected prevalence (%)	95% CI	Corrected prevalence (%)*	95% CI	Axis score
				Positive	Tested						
**Wickersham 2017[δ 49]**	**Malaysia**	**2014**	**NAAT-AC2**	**2**	**186**	**Genital**	**1.1**	**0.3, 3.8**	**0.9**	**0.2, 3.5**	**15**
**Budkaew 2019[50]**	**Thailand**	**2015-16**	**Taqman probe PCR**	**124**	**358**	**Genital**	**34.7**	**29.9, 39.7**	**35.5**	**30.7, 40.6**	**14**
Budkaew 2019[50]	Thailand	2015-16	Taqman probe PCR	100	358	Pharyngeal	27.9	23.5, 32.8	1.74	0.8, 3.7	14
Budkaew 2019[50]	Thailand	2015-16	Taqman probe PCR	104	358	Rectal	29.0	24.6, 34.0	35.6	30.8, 40.7	14
**Chen, 2021[51]**	**Vietnam**	**2016**	**Abbott RealTime PCR**	**69**	**205**	**Multisite**	**33.7**	**27.5, 40.4**	**37.4**	**31.1, 44.2**	**15**
Chen 2021[51]	Vietnam	2016	Abbott RealTime PCR	10	205	Genital	5.0	2.7, 8.7	5.2	2.9, 9.2	15
Chen 2021[51]	Vietnam	2016	Abbott RealTime PCR	18	205	Rectal	9.0	5.6, 13.5	11.0	7.4, 16.0	15
Chen 2021[51]	Vietnam	2016	Abbott RealTime PCR	57	205	Pharyngeal	27.8	22.1, 34.3	1.9	0.7, 4.8	15
**Hiransuthikul 2019[52]**	**Thailand**	**2015-2016**	**Abbott RealTime PCR**	**249**	**1,610**	**Multisite**	**15.5**	**13.8, 17.3**	**16.9**	**15.1, 18.8**	**13**
Hiransuthikul 2019[52]	Thailand	2015-2016	Abbott RealTime PCR	56	1,610	Genital	3.5	2.7, 4.5	3.6	2.8, 4.6	13
Hiransuthikul 2019[52]	Thailand	2015-2016	Abbott RealTime PCR	150	1,610	Rectal	9.3	8.0, 10.8	11.3	9.9, 13.0	13
Hiransuthikul 2019[52]	Thailand	2015-2016	Abbott RealTime PCR	110	1,610	Pharyngeal	6.8	5.7, 8.2	0.0	0.0, 0.2	13
**Yang 2018[φ53]**	**China**	**2015-17**	**NAAT-Roche Amplicor**	**34**	**153**	**Multisite**	**22.2**	**16.4, 29.4**	**24.3**	**18.2, 31.7**	**14**
**Yang 2018[θ53]**	**China**	**2015-17**	**NAAT-Roche Amplicor**	**24**	**310**	**Multisite**	**7.7**	**5.3, 11.3**	**8.2**	**5.6, 11.8**	**14**
Yang 2018[θ53]	China	2015-17	NAAT-Roche Amplicor	22	153	Genital	14.4	9.7, 20.8	15.7	10.7, 22.2	14
Yang 2018[θ53]	China	2015-17	NAAT-Roche Amplicor	11	153	Rectal	7.2	4.1, 12.4	8.7	5.2, 14.3	14
Yang 2018[θ53]	China	2015-17	NAAT-Roche Amplicor	6	153	Pharyngeal	3.90	1.8, 8.3	0.0	0.0, 2.5	14
Yang 2018[θ53]	China	2015-17	NAAT-Roche Amplicor	2	310	Genital	0.70	0.2, 2.3	0.44	0.1, 2.0	14
Yang 2018[θ53]	China	2015-17	NAAT-Roche Amplicor	17	310	Rectal	5.50	3.5, 8.6	6.65	4.4, 10.0	14
Yang 2018[θ53]	China	2015-17	NAAT-Roche Amplicor	12	310	Pharyngeal	3.90	2.2, 6.6	0.0	0.0, 1.2	14
**Adamson 2022[54]**	**Vietnam**	**2017-2019**	**Cobas Amplicor PCR**	**173**	**1,489**	**Multisite**	**11.6**	**10.1, 13.3**	**12.5**	**11.0, 14.3**	**14**
Adamson 2022[54]	Vietnam	2017-2019	Cobas Amplicor PCR	5	1,480	Genital	0.3	0.1, 8.8	0.0	0.0, 0.3	14
Adamson 2022[54]	Vietnam	2017-2019	Cobas Amplicor PCR	42	1,419	Rectal	3.0	2.2, 4.0	3.6	2.7, 4.7	14
Adamson 2022[54]	Vietnam	2017-2019	Cobas Amplicor PCR	80	1,486	Pharyngeal	5.4	4.3, 6.7	0.0	0.0, 0.3	14
**Songtaweesin 2022[55]**	**Thailand**	**2018-2019**	**Abbott RealTime PCR**	**9**	**200**	**Multisite**	**4.5**	**2.4, 8.3**	**4.7**	**2.5, 8.5**	**11**
Songtaweesin 2022[55]	Thailand	2018-2019	Abbott RealTime PCR	2	200	Genital	1.0	0.3, 3.6	0.8	0.2, 0.9	11
Songtaweesin 2022[55]	Thailand	2018-2019	Abbott RealTime PCR	7	200	Rectal	3.5	1.7, 7.0	4.2	2.2, 7.9	11
**Zhou 2019[56]**	**China**	**2017-2018**	**Cobas Amplicor PCR**	**29**	**379**	**Multisite**	**7.7**	**5.4, 10.8**	**8.2**	**5.9, 11.4**	**17**
Zhou 2019[56]	China	2017-2018	Cobas Amplicor PCR	3	379	Genital	0.8	0.3, 2.3	0.6	0.2, 1.9	17
Zhou 2019[56]	China	2017-2018	Cobas Amplicor PCR	19	379	Rectal	5.0	3.2, 7.7	6.0	4.0, 8.9	17
Zhou 2019[56]	China	2017-2018	Cobas Amplicor PCR	8	379	Pharyngeal	2.1	1.1, 4.1	0.0	0.0, 1.0	17
**Latin America and the Caribbean**											
**Clark 2007[57]**	**Peru**	**2000-01**	**NAAT-Roche Amplicor**	**0**	**85**	**Genital**	**0.0**	**0.0, 4.3**	**0.0**	**0.0, 4.3**	**13**
**Wong 2013[58]**	**Hong Kong SAR**	**2011**	**NAAT-AC2**	**2**	**994**	**Genital**	**0.2**	**0.1, 0.7**	**0.0**	**0.0, 0.4**	**14**
**Hananta 2016[59]**	**Indonesia**	**2014**	**PCR**	**49**	**272**	**Genital**	**17.8**	**13.9, 23.0**	**17.7**	**13.6, 22.6**	**12**

1. Gonorrhoea point prevalence data (continued)

Reference	Country	Year(s)	Diagnostic method	No. of MSM		Anatomical sample site	Uncorrected prevalence (%)	95% CI	Corrected prevalence (%)*	95% CI	Axis score
				Positive	Tested						
**Soto 2007[60]**	**Multiple[Δ]**	**2001-02**	**Cobas Amplicor PCR**	**34**	**1,409**	**Pharyngeal**	**2.4**	**1.7, 3.5**	**2.3**	**1.7, 3.3**	**12**
**Clark 2009[61]**	**Peru**	**2003**	**NAAT-Roche Amplicor**	**2**	**541**	**Genital**	**0.4**	**0.1, 1.3**	**0.1**	**0.0, 0.9**	**13**
**Konda 2013[χ,62]**	**Peru**	**2003-07**	**NAAT-Roche Amplicor**	**3**	**193**	**Genital**	**1.6**	**0.5, 4.5**	**1.4**	**0.5, 4.3**	**12**
**Konda 2013[56]**	**Peru**	**2003-07**	**NAAT-Roche Amplicor**	**0**	**75**	**Genital**	**0.0**	**0.0, 4.9**	**0.0**	**0.0, 4.9**	**12**
**Garcia 2018[63]**	**Guatemala**	**2007**	**Culture**	**1**	**12**	**Genital**	**8.3**	**1.5, 35.4**	**9.2**	**1.8, 36.4**	**12**
**Garcia 2018[63]**	**Guatemala**	**2008**	**Culture**	**22**	**50**	**Genital**	**44.0**	**31.2, 57.7**	**48.6**	**35.4, 62.1**	**12**
**Garcia 2018[63]**	**Guatemala**	**2009**	**Culture**	**24**	**90**	**Genital**	**26.7**	**18.6, 36.6**	**29.5**	**21.0, 39.6**	**12**
**Garcia 2018[63]**	**Guatemala**	**2010**	**Culture**	**10**	**79**	**Genital**	**12.7**	**7.0, 21.8**	**14.0**	**8.0, 23.3**	**12**
**Garcia 2018[63]**	**Guatemala**	**2011**	**Culture**	**6**	**78**	**Genital**	**7.7**	**3.6, 15.8**	**8.5**	**4.1, 16.8**	**12**
**Garcia 2018[63]**	**Guatemala**	**2012**	**Culture**	**21**	**60**	**Genital**	**35.0**	**24.2, 47.6**	**38.7**	**27.4, 51.3**	**12**
**Perez-Brumer 2013[64]**	**Peru**	**2007**	**NAAT-Roche Amplicor**	**12**	**560**	**Genital**	**2.1**	**1.2, 3.7**	**2.0**	**1.1, 3.5**	**13**
**Figueroa 2013[65]**	**Jamaica**	**2007-08**	**NAAT-AC2**	**7**	**201**	**Genital**	**3.5**	**1.7, 7.0**	**3.6**	**1.7, 7.1**	**10**
**Creswell 2012[66]**	**El Salvador[∇]**	**2008**	**PCR**	**1**	**116**	**Rectal**	**0.9**	**0.2, 4.7**	**1.0**	**0.2, 4.9**	**15**
**Creswell 2012[66]**	**El Salvador[∇]**	**2008**	**PCR**	**17**	**390**	**Rectal**	**4.4**	**2.7, 6.9**	**5.3**	**3.5, 8.0**	**15**
Creswell 2012[66]	El Salvador	2008	PCR	8	460	Genital	1.7	0.9, 3.4	1.6	0.8, 3.2	15
Creswell 2012[66]	El Salvador	2008	PCR	0	188	Genital	0.0	0.0, 2.0	0	0.0, 2.0	15
**Leon 2013[67]**	**Peru**	**2008**	**NAAT-AC2**	**69**	**718**	**Rectal**	**9.6**	**7.7, 12.0**	**11.7**	**9.5, 14.3**	**7**
Leon 2013[67]	Peru	2008	NAAT-AC2	47	718	Pharyngeal	6.5	5.0, 8.6	0.0	0.0, 0.5	7
**Castillo 2015[68]**	**Peru**	**2009**	**NAAT-AC2**	**68**	**713**	**Rectal**	**9.6**	**7.6, 11.9**	**11.7**	**9.5, 14.3**	**12**
Castillo 2015[68]	Peru	2009	NAAT-AC2	46	713	Pharyngeal	6.5	4.9, 8.5	0.0	0.0, 0.5	12
**Cunha 2015[69]**	**Brazil**	**2010-12**	**NAAT-AC2**	**7**	**279**	**Rectal**	**2.5**	**1.2, 5.1**	**3.0**	**1.5, 5.7**	**14**
Cunha 2015[69]	Brazil	2010-12	NAAT-AC2	0	273	Genital	0.0	0.0, 1.4	0.0	0.0, 1.4	14
**Allan Blitz 2017[70]**	**Peru**	**2010-14**	**NAAT-AC2**	**34**	**387**	**Genital**	**8.8**	**6.4, 12.0**	**10.7**	**8.0, 14.2**	**12**
Allan Blitz 2017[70]	Peru	2010-14	NAAT-AC2	25	387	Genital	6.5	4.4, 9.4	0.0	0.0, 1.0	12
**Galarraga 2014[λ,71]**	**Mexico**	**2012**	**NAAT-AC2**	**6**	**267**	**Genital**	**2.3**	**1.0, 4.8**	**2.2**	**1.0, 4.7**	**14**
**Morales-Miranda 2013[72]**	**Belize**	**2012**	**PCR**	**4**	**130**	**Rectal**	**2.9**	**1.2, 7.6**	**3.4**	**1.4, 8.2**	**8**
**Kojima 2017[73]**	**Peru**	**2012-13**	**NAAT-AC2**	**44**	**312**	**Multisite**	**14.2**	**10.7, 18.4**	**17.4**	**13.6, 22.0**	**14**
Kojima 2017[73]	Peru	2012-13	NAAT-AC2	26	312	Rectal	5.8	3.7, 8.9	0.0	0.0, 1.2	14
Kojima 2017[73]	Peru	2012-13	NAAT-AC2	18	312	Pharyngeal	8.4	5.7, 11.9	10.22	7.3, 14.1	14
**Cabeza 2015[74]**	**Peru**	**2012-14**	**NAAT-AC2**	**78**	**834**	**Pharyngeal**	**9.4**	**7.6, 11.5**	**0.0**	**0.0, 0.5**	**9**
Cabeza 2015[74]	Peru	2012-14	NAAT-AC2	69	834	Rectal	8.3	6.6, 10.3	10.1	8.2, 12.3	9
Cabeza 2015[74]	Peru	2012-14	NAAT-AC2	23	834	Genital	2.7	1.8, 4.1	2.66	1.8, 4.0	9
**Passaro 2018[75]**	**Peru**	**2012-14**	**NAAT-AC2**	**125**	**787**	**Rectal**	**15.3**	**12.9, 17.9**	**16.6**	**14.2, 19.4**	**11**
Passaro 2018[75]	Peru	2012-14	NAAT-AC2	78	787	Pharyngeal	9.9	8.0, 12.2	0.0	0.0, 0.5	11
Passaro 2018[75]	Peru	2012-14	NAAT-AC2	19	787	Genital	2.4	1.6, 3.7	2.33	1.5, 3.6	11

1. Gonorrhoea point prevalence data (continued)

Reference	Country	Year(s)	Diagnostic method	No. of MSM		Anatomical sample site	Uncorrected prevalence (%)	95% CI	Corrected prevalence (%)*	95% CI	Axis score
				Positive	Tested						
Chow 2017[76]	Peru	2013-14	NAAT-AC2	26	312	Rectal	8.3	5.6, 11.9	10.1	7.2 13.9	11
Mendizabal-Burastero 2015[77]	Guatemala	2014	PCR-Digene Hybrid Capture	133	524	Pharyngeal	25.4	21.8, 29.3	0.0	0.0, 0.7	7
Mendizabal-Burastero 2015[77]	Guatemala	2014	PCR-Digene Hybrid Capture	21	524	Genital	4.1	2.6, 6.0	3.26	2.1, 5.2	7
Mendizabal-Burastero 2015[77]	Guatemala	2014	PCR-Digene Hybrid Capture	23	524	Rectal	4.4	2.9, 6.5	5.3	3.7, 7.6	7
Grinsztejn 2017ϕ[78]	Brazil	2015	PCR	25	345	Rectal	7.6	5.0, 10.5	9.2	6.6, 12.8	17
Bristow 2021[79]	Mexico	2017-2018	GeneXpert CT/NG	35	212	Multisite	16.5	12.1, 22.1	18.0	13.4, 23.7	14
Bristow 2021[79]	Mexico	2017-2018	GeneXpert CT/NG	9	212	Genital	4.2	2.2, 7.9	4.3	2.3, 8.0	14
Bristow 2021[79]	Mexico	2017-2018	GeneXpert CT/NG	26	212	Rectal	12.3	8.5, 17.4	15.0	10.8, 20.5	14
Bristow 2021[79]	Mexico	2017-2018	GeneXpert CT/NG	16	212	Pharyngeal	7.6	4.7, 11.9	0.0	0.0, 1.8	14
Moriarty 2019[80]	Peru	2017	Gen-Probe Aptima	13	120	Rectal	10.8	6.4, 17.7	13.2	8.3, 20.4	15
Jean Louis 2020[81]	Haiti	2018-2019	GeneXpert CT/NG	24	216	Multisite	16.2	11.9, 21.7	17.6	13.1, 23.3	16
Jean Louis 2020[81]	Haiti	2018-2019	GeneXpert CT/NG	12	216	Genital	5.6	3.2, 9.5	5.8	3.4, 9.8	16
Jean Louis 2020[81]	Haiti	2018-2019	GeneXpert CT/NG	25	216	Rectal	11.6	8.0, 16.5	14.1	10.1, 19.4	16
**Australia and New Zealand**											
Lister 2004[82]	Australia	2001-02	Culture	5	71	Genital	7.0	3.1, 15.5	7.7	3.5, 16.3	11
Lister 2004[82]	Australia	2001-02	Culture	2	50	Rectal	4.0	1.1, 13.5	4.4	1.3, 14.1	11
Lister 2004[82]	Australia	2001-02	Culture	3	69	Pharyngeal	4.3	1.5, 12.0	4.8	1.7, 12.6	11
Lister 2003[83]	Australia	2001-02	NAAT-Cobas Amplicor	13	521	Pharyngeal	2.5	1.5, 4.2	0.0	0.0, 0.7	14
Lister 2003[83]	Australia	2001-02	NAAT-Cobas Amplicor	1	511	Genital	0.2	0.0, 1.1	0.0	0.0, 0.7	14
Lister 2003[83]	Australia	2001-02	NAAT-Cobas Amplicor	11	507	Rectal	2.2	1.2, 3.8	0.0	0.0, 0.7	14
Hamlyn 2006[84]	Australia	2001-04	SDA (BD ProbeTec)	6	253	Rectal	2.5	1.1, 5.1	3.2	1.6, 6.2	13
Hamlyn 2006[84]	Australia	2001-04	SDA (BD ProbeTec)	4	253	Genital	1.5	0.6, 4.0	1.0	0.3, 3.2	13
Hamlyn 2006[84]	Australia	2001-04	SDA (BD ProbeTec)	6	253	Pharyngeal	2	1.1, 5.1	2.0	0.9, 4.6	13
Jin 2007[85]	Australia	2001-06	SDA	18	1,419	Rectal	1.3	0.8, 2.0	1.7	1.1, 2.5	14
Jin 2007[85]	Australia	2001-06	SDA	4	1,419	Genital	0.3	0.1, 0.7	0.0	0.0, 0.3	14
Lister 2005[86]	Australia	2002	Culture	3	207	Rectal	1.4	0.5, 4.2	1.5	0.5, 4.3	14
Lister 2005[86]	Australia	2002	Culture	3	276	Genital	1.1	0.4, 3.2	1.2	0.4, 3.3	14
Lister 2005[86]	Australia	2002	Culture	0	280	Pharyngeal	1.4	0.5, 4.2	1.6	0.5, 4.3	14
Ryder 2010[87]	Australia	2006-08	NAAT-Roche Amplicor	2	4,454	Genital	0.0	0.0, 0.2	0.0	0.0, 0.1	11
Goddard 2019[88]	Australia	2010-15	AC2 NAAT	3	617	Rectal	0.5	0.2, 1.4	0.5	0.2, 1.4	14
Cornelisse 2018[89]	Australia	2013	Culture TM	48	990	Rectal	4.8	3.7, 6.4	5.3	4.1, 6.9	15
Cornelisse 2018	Australia	2013	Culture TM	26	990	Pharyngeal	2.6	1.8, 3.8	2.9	2.0, 4.1	15
Martin-Sanchez 2020χ[90]	Australia	2011-2018	SDA (BD ProbeTec)	1,461	13,780	Multisite	10.6	10.1, 11.1	11.7	11.2, 12.3	14
Martin-Sanchez 2020[90]	Australia	2011-2018	SDA (BD ProbeTec)	1,321	11,951	Multisite	11.1	10.5, 11.6	12.3	12.7, 12.9	14
Martin-Sanchez 2020[90]	Australia	2011-2018	SDA (BD ProbeTec)	390	11,699	Genital	3.3	3.0, 3.7	3.6	3.3, 4.0	14

1. Gonorrhoea point prevalence data (continued)

Reference	Country	Year(s)	Diagnostic method	No. of MSM		Anatomical sample site	Uncorrected prevalence (%)	95% CI	Corrected prevalence (%)*	95% CI	Axis score
				Positive	Tested						
Martin-Sanchez 2020[90]	Australia	2011-2018	SDA (BD ProbeTec)	745	11,080	Rectal	6.7	6.3, 7.2	7.4	6.9, 7.9	14
Martin-Sanchez 2020[90]	Australia	2011-2018	SDA (BD ProbeTec)	682	11,810	Pharyngeal	5.8	5.4, 6.2	6.4	6.0, 6.9	14
Martin-Sanchez 2020[χ90]	Australia	2011-2018	SDA (BD ProbeTec)	442	13,584	Genital	3.3	3.0, 3.6	3.6	3.3, 3.9	14
Martin-Sanchez 2020[χ90]	Australia	2011-2018	SDA (BD ProbeTec)	803	12,484	Rectal	6.4	6.0, 6.9	7.1	6.6, 7.5	14
Martin-Sanchez 2020[χ90]	Australia	2011-2018	SDA (BD ProbeTec)	750	13,521	Pharyngeal	5.6	5.2, 5.9	6.1	5.7, 6.5	14
**Chow 2017[91]**	**Australia**	**2015**	**NAAT**	**83**	**823**	**Pharyngeal**	**10.1**	**8.2, 12.3**	**0.0**	**0.0, 0.5**	**14**
**Cornelisse 2018[92]**	**Australia**	**2015**	**NAAT**	**94**	**375**	**Pharyngeal**	**25.1**	**21.0, 29.7**	**0.0**	**0.0, 1.0**	**14**
Cornelisse 2018[92]	Australia	2015	NAAT	147	393	Rectal	12.0	32.8, 42.3	14.6	11.5, 18.4	14
**Ong 2017[93]**	**Australia**	**2015**	**AC2 NAAT**	**228**	**5,497**	**Genital**	**4.2**	**3.7, 4.7**	**4.3**	**3.8, 4.8**	**13**
**Cornelisse 2018[94]**	**Australia**	**2016-18**	**AC2 NAAT**	**70**	**1,772**	**Rectal**	**4.0**	**3.1, 5.0**	**4.1**	**3.3, 5.1**	**16**
**Saxton 2022[95]**	**New Zealand**	**2017-2019**	**SDA (BD ProbeTec)**	**21**	**150**	**Multisite**	**14.0**	**9.3, 20.5**	**13.9**	**9.3, 20.4**	**15**
**Tabesh 2022[96]**	**Australia**	**2018-2019**	**AC2 NAAT**	**447**	**3,938**	**Multisite**	**11.4**	**10.4, 12.4**	**12.3**	**11.3, 13.4**	**13**
Tabesh 2022[96]	Australia	2018-2019	AC2 NAAT	100	3,938	Genital	2.5	2.1, 3.1	2.4	2.0, 3.0	13
Tabesh 2022[96]	Australia	2018-2019	AC2 NAAT	309	3,938	Rectal	7.8	7.0, 8.7	9.5	8.6, 10.4	13
Tabesh 2022[96]	Australia	2018-2019	AC2 NAAT	262	3,938	Pharyngeal	6.6	5.9, 7.5	0.0	0.0, 0.1	13
**Oceania (excluding Australia and New Zealand)**											
**Badman 2018[97]**	**Papua New Guinea**	**2016**	**Xpert CT/NG**	**43**	**733**	**Rectal**	**5.9**	**4.4, 7.8**	**7.1**	**5.5, 9.2**	**15**
**Hakim 2021[98]**	**Papua New Guinea[∇]**	**2017**	**GeneXpert Ct/NG**	**40**	**400**	**Multisite**	**10.0**	**7.4, 13.3**	**11.1**	**8.4, 14.6**	**15**
**Hakim 2021[98]**	**Papua New Guinea[∇]**	**2017**	**GeneXpert Ct/NG**	**33**	**352**	**Multisite**	**9.4**	**6.8, 12.9**	**10.1**	**7.4, 13.7**	**15**
**Hakim 2021[98]**	**Papua New Guinea[∇]**	**2017**	**GeneXpert Ct/NG**	**7**	**111**	**Multisite**	**6.3**	**3.1, 12.4**	**10.3**	**5.9, 17.4**	**15**
Hakim 2021[98]	Papua New Guinea	2017	GeneXpert Ct/NG	13	400	Genital	3.2	1.9, 5.5	3.2	1.9, 5.4	15
Hakim 2021[98]	Papua New Guinea	2017	GeneXpert Ct/NG	25	400	Rectal	6.3	4.3, 9.1	8.0	5.7, 11.1	15
Hakim 2021[98]	Papua New Guinea	2017	GeneXpert Ct/NG	17	352	Genital	4.9	3.0, 7.6	5.1	3.3, 7.9	15
Hakim 2021[98]	Papua New Guinea	2017	GeneXpert Ct/NG	6	352	Rectal	1.7	0.8, 3.7	2.2	1.1, 4.3	15
Hakim 2021[98]	Papua New Guinea	2017	GeneXpert Ct/NG	0	111	Genital	0.0	0.0, 3.3	0.0	0.0, 3.3	15
Hakim 2021[98]	Papua New Guinea	2017	GeneXpert Ct/NG	4	111	Rectal	6.2	4.3, 9.1	7.5	3.9, 14.0	15
**Europe and North America**											
**Moncada 2009[3]**	**USA**	**NR**	**NAAT-AC2**	**108**	**882**	**Genital**	**12.2**	**10.2, 14.6**	**13.2**	**11.1, 15.6**	**12**
Moncada 2009[3]	USA	NR	NAAT-AC2	83	882	Rectal	9.4	7.7, 11.5	11.5	9.5, 13.7	12
**Moncada 2015[99]**	**USA**	**NR**	**NAAT-AC2**	**32**	**260**	**Multisite**	**12.3**	**8.9, 16.9**	**13.3**	**9.7, 18.0**	**12**
**Sexton 2011[100]**	**USA**	**NR**	**NAAT-AC2**	**25**	**286**	**Pharyngeal**	**8.9**	**6.0, 12.6**	**0.0**	**0.0, 1.3**	**12**
Sexton 2011[100]	USA	NR	NAAT-AC2	24	286	Rectal	8.5	5.7, 12.2	10.3	7.3, 14.4	12
**Benn 2007[101]**	**England**	**1999-2001**	**LCR**	**96**	**599**	**Multisite**	**16.0**	**13.3, 19.2**	**15.5**	**12.9, 18.7**	**11**

1. Gonorrhoea point prevalence data (continued)

Reference	Country	Year(s)	Diagnostic method	No. of MSM		Anatomical sample site	Uncorrected prevalence (%)	95% CI	Corrected prevalence (%)*	95% CI	Axis score
				Positive	Tested						
Benn 2007[101]	England	1999-2001	LCR	43	599	Genital	7.2	5.4, 9.5	6.4	4.7, 8.7	11
Benn 2007[101]	England	1999-2001	LCR	44	599	Pharyngeal	7.3	5.5, 9.7	9.1	7.1, 11.7	11
Benn 2007[101]	England	1999-2001	LCR	44	599	Rectal	7.3	5.5, 9.7	9.4	7.3, 12.0	11
**Leuridan 2005[λ102]**	**Belgium**	**1999-2004**	**NAAT-Cobas Amplicor**	**2**	**115**	**Genital**	**2.6**	**0.5, 6.1**	**2.6**	**0.9, 7.3**	**11**
**Bloomfield 2002[α103]**	**USA**	**2000**	**SDA (BD ProbeTec)**	**3**	**76**	**Genital**	**3.9**	**1.4, 11.0**	**3.5**	**1.1, 10.4**	**12**
Bloomfield 2002[α]	USA	2000	GenProbe APTIMA	16	76	Genital	4.1	2.5, 6.5	4.2	2.6, 6.6	12
**Kim 2003[104]**	**USA**	**2000**	**Culture**	**40**	**564**	**Rectal**	**7.1**	**5.3, 9.5**	**7.8**	**5.9, 10.4**	**13**
**Sethi 2006[105]**	**England**	**2000-03**	**SDA**	**36**	**279**	**Multisite**	**12.9**	**9.5, 17.4**	**12.8**	**9.3, 17.2**	**14**
**Javanbakht 2009[106]**	**USA**	**2000-05**	**NAAT**	**69**	**4,106**	**Genital**	**1.7**	**1.3, 2.1**	**1.6**	**1.2, 2.0**	**10**
**Dilley 2003[107]**	**USA**	**2001**	**NAAT**	**124**	**2,615**	**Genital**	**4.7**	**4.0, 5.6**	**4.9**	**4.1, 5.8**	**11**
**Russell 2007[108]**	**USA**	**2002**	**LCR**	**0**	**206**	**Genital**	**0.0**	**0.0, 1.8**	**0.0**	**0.0, 1.8**	**13**
**Vall-Mayans 2007[109]**	**Spain**	**2002-03**	**PCR**	**9**	**132**	**Genital**	**6.8**	**3.6, 12.5**	**7.2**	**3.9, 12.9**	**12**
**Kent 2005[110θ]**	**USA**	**2003**	**SDA (BD ProbeTec)**	**59**	**761**	**Pharyngeal**	**7.8**	**6.1, 9.9**	**9.8**	**7.9, 12.1**	**12**
**Kent 2005[φ]**	**USA**	**2003**	**SDA (BD ProbeTec)**	**439**	**4,665**	**Pharyngeal**	**9.4**	**8.6, 10.3**	**12.0**	**11.1, 12.9**	**12**
Kent 2005	USA	2003	SDA (BD ProbeTec)	15	783	Genital	1.9	1.2, 3.1	1.4	0.8, 2.6	12
Kent 2005	USA	2003	SDA (BD ProbeTec)	349	5,283	Genital	6.6	6.0, 7.3	6.3	5.7, 7.0	12
Kent 2005	USA	2003	SDA (BD ProbeTec)	17	525	Rectal	3.2	2.0, 5.1	4.1	2.7, 6.2	12
Kent 2005	USA	2003	SDA (BD ProbeTec)	248	3,300	Rectal	7.5	6.7, 8.5	9.6	8.7, 10.7	12
**Mayer 2012[111]**	**USA**	**2004-10**	**NAAT-AC2**	**1**	**889**	**Genital**	**0.1**	**0.0, 0.6**	**0.0**	**0.0, 0.4**	**13**
**Annan 2009[112]**	**England**	**2005-06**	**Culture**	**142**	**3,076**	**Genital**	**4.6**	**3.9, 5.4**	**5.1**	**4.4, 5.9**	**11**
Annan 2009[112]	England	2005-06	Culture	125	3,017	Rectal	4.1	3.5, 4.9	4.5	3.8, 5.3	11
**Bozicevic 2008[113]**	**Croatia**	**2006**	**NAAT-AC2**	**42**	**359**	**Pharyngeal**	**11.7**	**8.8, 15.4**	**12.7**	**9.6, 16.5**	**11**
**Baker 2009[114]**	**USA**	**2006-07**	**SDA (BD ProbeTec)**	**6**	**147**	**Multisite**	**4.1**	**1.9, 8.6**	**3.7**	**1.7, 8.1**	**9**
Baker 2009[114]	USA	2006-07	SDA (BD ProbeTec)	2	147	Rectal	1.4	0.4, 4.8	1.8	0.6, 5.5	9
Baker 2009[114]	USA	2006-07	SDA (BD ProbeTec)	0	147	Genital	0.0	0.0, 2.6	0.0	0.0, 2.6	9
Baker 2009[114]	USA	2006-07	SDA (BD ProbeTec)	4	147	Pharyngeal	2.8	1.1, 6.8	3.1	1.3, 7.3	9
**Ota 2009[115]**	**Canada**	**2006-08**	**NAAT-AC2**	**29**	**248**	**Rectal**	**11.7**	**8.3, 16.3**	**14.3**	**10.5, 19.2**	**13**
Ota 2009[115]	Canada	2006-08	NAAT-AC2	20	248	Pharyngeal	8.1	5.3, 12.1	0.0	0.0, 1.5	13
Ota 2009[115]	Canada	2006-08	NAAT-AC2	17	248	Genital	6.8	4.3, 10.7	7.2	4.6, 11.1	13
**Bernstein 2009[116]**	**USA**	**2007**	**GenProbe APTIMA**	**16**	**395**	**Genital**	**4.0**	**2.5, 6.5**	**4.2**	**2.6, 6.6**	**12**
**Klausner 2009[117]**	**USA**	**2007**	**Culture**	**34**	**2,168**	**Pharyngeal**	**1.6**	**1.1, 2.2**	**1.8**	**1.3, 2.4**	**12**
**Klausner 2009[117]**	**USA**	**2007**	**NAAT**	**343**	**4,822**	**Rectal**	**7.1**	**6.4, 7.9**	**8.6**	**7.9, 9.4**	**12**
Klausner 2009[117]	USA	2007	Culture	725	12,021	Pharyngeal	6.0	5.6, 6.5	0.0	0.0, 0.0	12
Klausner 2009[117]	USA	2007	NAAT	10	1,677	Rectal	0.6	0.3, 1.1	0.7	0.4, 1.2	12
**Mimiagi 2008[118]**	**USA**	**2007**	**SDA (BD ProbeTec)**	**2**	**111**	**Rectal**	**1.7**	**0.5, 6.3**	**2.2**	**0.7, 6.9**	**13**

1. Gonorrhoea point prevalence data (continued)

Reference	Country	Year(s)	Diagnostic method	No. of MSM		Anatomical sample site	Uncorrected prevalence (%)	95% CI	Corrected prevalence (%)*	95% CI	Axis score
				Positive	Tested						
Mimiagi 2008[118]	USA	2007	SDA (BD ProbeTec)	1	113	Genital	1.0	0.2, 4.8	0.5	0.1, 4.2	13
**Fisher 2015[119]**	**England**	**2008**	**NAAT-AC2**	**5**	**202**	**Multisite**	**2.5**	**1.1, 5.7**	**2.4**	**1.0, 5.6**	**16**
Fisher 2015[119]	England	2008	NAAT-AC2	3	202	Pharyngeal	1.5	0.5, 4.3	0.0	0.0, 1.9	16
Fisher 2015[119]	England	2008	NAAT-AC2	1	202	Genital	0.5	0.1, 2.8	0.2	0.0, 2.3	16
Fisher 2015[119]	England	2008	NAAT-AC2	1	202	Rectal	0.5	0.1, 2.8	0.5	0.1, 2.8	16
**Soni 2009[120]**	**England**	**2008**	**SDA (BD ProbeTec)**	**23**	**850**	**Multisite**	**5.0**	**1.8, 4.0**	**4.6**	**3.4, 6.3**	**13**
Soni 2009[120]	England	2008	SDA (BD ProbeTec)	13	412	Rectal	3.2	1.9, 5.3	4.1	2.6, 6.5	13
Soni 2009[120]	England	2008	SDA (BD ProbeTec)	10	438	Genital	2.3	1.2, 4.2	1.9	1.0, 3.6	13
**Freeman 2011[121]**	**USA**	**2009**	**NAAT-AC2**	**32**	**473**	**Pharyngeal**	**6.8**	**4.8, 9.4**	**0.0**	**0.0, 0.8**	**11**
**Cuypers 2011[λ122]**	**Netherlands**	**2009-10**	**NAAT**	**12**	**99**	**NR**	**12.1**	**7.1, 20.0**	**11.7**	**6.7, 19.5**	**9**
**Dudareva-Vizule 2014[123]**	**Germany**	**2009-10**	**NAAT-AC2**	**121**	**2,197**	**Pharyngeal**	**5.5**	**4.6, 6.5**	**0.0**	**0.0, 0.2**	**15**
Dudareva-Vizule 2014[123]	Germany	2009-10	NAAT-AC2	13	685	Genital	1.9	1.1, 3.2	1.0	0.5, 2.0	15
Dudareva-Vizule 2014[123]	Germany	2009-10	NAAT-AC2	95	2,050	Rectal	4.6	3.8, 5.6	5.5	4.6, 6.6	15
**Mayer 2014[124]**	**USA**	**2010**	**NAAT-AC2**	**47**	**1,553**	**Rectal**	**3.0**	**2.3, 4.0**	**3.6**	**2.8, 4.6**	**13**
Mayer 2014[124]	USA	2010	NAAT-AC2	16	1,553	Genital	1.0	0.6, 1.77	0.8	0.5, 1.4	13
**Reinton 2013[125]**	**Norway**	**2009-11**	**NAAT-Roche Amplicor**	**136**	**2,262**	**Multisite**	**6.0**	**5.0, 7.0**	**6.3**	**5.4, 7.4**	**13**
Reinton 2013[125]	Norway	2010	NAAT-Roche Amplicor	77	2,262	Pharyngeal	3.4	2.7, 4.2	0.0	0.0, 0.2	13
Reinton 2013[125]	Norway	2010	NAAT-Roche Amplicor	79	2,262	Rectal	3.5	2.8, 4.3	4.3	3.6, 5.2	13
Reinton 2013[125]	Norway	2010	NAAT-Roche Amplicor	30	2,262	Genital	1.3	0.9, 1.9	1.3	0.9, 1.9	13
**Park 2012[126]**	**USA**	**2010**	**NAAT-AC2**	**718**	**12,457**	**Pharyngeal**	**5.8**	**5.4, 6.2**	**0.0**	**0.0, 0.0**	**13**
**Perkins 2011[θ127]**	**USA**	**2010**	**NAAT**	**6**	**189**	**Pharyngeal**	**3.2**	**1.5, 6.8**	**0.0**	**0.0, 2.0**	**9**
Perkins 2011[φ127]	USA	2010	NAAT	89	1,138	Rectal	7.8	6.4, 9.5	9.5	7.9, 11.4	9
Perkins 2011[φ127]	USA	2010	NAAT	115	1,753	Genital	6.7	5.5, 7.8	7.0	5.9, 8.2	9
Perkins 2011[φ127]	USA	2010	NAAT	2	183	Rectal	1.1	0.3, 3.9	1.2	0.4, 4.1	9
Perkins 2011[φ127]	USA	2010	NAAT	2	184	Genital	1.1	0.3, 3.9	0.9	0.2, 3.6	9
**Bozicevic 2012[128]**	**Croatia**	**2010-11**	**Abbott Realtime PCR**	**8**	**387**	**Pharyngeal**	**2.1**	**1.1, 4.0**	**2.0**	**1.0, 3.9**	**11**
**Van Liere 2013[129]**	**Netherlands**	**2010-11**	**SDA (BD ProbeTec)**	**41**	**674**	**Multisite**	**6.1**	**4.5, 8.2**	**5.8**	**4.3, 7.8**	**14**
**Van Liere 2013[χ129]**	**Netherlands**	**2010-11**	**SDA (BD ProbeTec)**	**9**	**252**	**Multisite**	**3.6**	**1.9, 6.7**	**3.2**	**1.6, 6.2**	**14**
**Remis 2016[130]**	**Canada**	**2010-12**	**SDA (BD ProbeTec)**	**0**	**147**	**Genital**	**0.0**	**0.0, 2.6**	**0.0**	**0.0, 2.5**	**11**
**Van Liere 2015[131]**	**Netherlands**	**2011-12**	**NAAT-Cobas Amplicor**	**397**	**9,534**	**Rectal**	**4.2**	**3.8, 4.6**	**5.0**	**4.6, 5.5**	**13**
**Van Rooijen 2015[132]**	**Netherlands**	**2011-12**	**NAAT-AC2**	**724**	**13,111**	**Pharyngeal**	**5.5**	**5.2, 5.9**	**0.0**	**0.0, 0.0**	**13**
Van Rooijen 2015[132]	Netherlands	2011-12	NAAT-AC2	362	13,074	Genital	2.8	2.5, 3.1	2.8	2.5, 3.1	13
Van Rooijen 2015[132]	Netherlands	2011-12	NAAT-AC2	587	13,039	Rectal	4.5	4.2, 4.9	5.4	5.0, 5.8	13
**Gratrix 2014[133]**	**Canada**	**2012**	**NAAT-AC2**	**63**	**972**	**Multisite**	**6.5**	**5.1, 8.2**	**6.9**	**5.5, 8.6**	**11**
Gratrix 2014[133]	Canada	2012	NAAT-AC2	23	972	Genital	2.4	1.6, 3.5	2.3	1.6, 3.5	11

1. Gonorrhoea point prevalence data (continued)

Reference	Country	Year(s)	Diagnostic method	No. of MSM		Anatomical sample site	Uncorrected prevalence (%)	95% CI	Corrected prevalence (%)*	95% CI	Axis score
				Positive	Tested						
Gratrix 2014[133]	Canada	2012	NAAT-AC2	57	972	Rectal	5.9	4.6, 7.5	7.1	5.7, 8.9	11
**Jeverica 2013[134]**	**Slovenia**	**2012**	**Culture**	**13**	**306**	**Multisite**	**4.3**	**2.5, 7.1**	**4.8**	**2.9, 7.7**	**9**
Jeverica 2013[134]	Slovenia	2012	Culture	3	167	Pharyngeal	1.7	0.6, 5.2	1.9	0.7, 5.3	9
Jeverica 2013[134]	Slovenia	2012	Culture	0	167	Genital	0.0	0.0, 2.3	0.0	0.0, 2.3	9
Jeverica 2013[134]	Slovenia	2012	Culture	15	167	Rectal	9.2	5.5, 14.3	10.2	6.4, 15.7	9
Jeverica 2013[134]	Slovenia	2012	Culture	6	239	Pharyngeal	2.5	1.2, 5.4	2.8	1.3, 5.7	9
**Bamberger 2019[135]**	**USA**	**2012-2014**	**AC2 NAAT**	**163**	**1,002**	**Multisite**	**16.3**	**14.1, 18.7**	**17.8**	**15.5, 20.2**	**9**
**Bamberger 2019[χ135]**	**USA**	**2012-2014**	**AC2 NAAT**	**32**	**215**	**Multisite**	**14.9**	**10.7, 20.3**	**16.2**	**11.9, 21.7**	**9**
Bamberger 2019[135]	USA	2012-2014	AC2 NAAT	52	969	Genital	5.4	4.1, 7.0	5.6	4.3, 7.3	9
Bamberger 2019[135]	USA	2012-2014	AC2 NAAT	104	632	Rectal	16.5	13.8, 19.5	20.1	17.2, 23.4	9
Bamberger 2019[135]	USA	2012-2014	AC2 NAAT	82	967	Pharyngeal	8.5	6.9, 10.4	0.0	0.0, 1.8	9
Bamberger 2019[χ135]	USA	2012-2014	AC2 NAAT	16	209	Genital	7.7	4.8, 12.1	8.2	5.2, 12.7	9
Bamberger 2019[χ135]	USA	2012-2014	AC2 NAAT	14	89	Rectal	15.7	9.6, 24.7	19.2	12.4, 28.6	9
Bamberger 2019[χ135]	USA	2012-2014	AC2 NAAT	14	211	Pharyngeal	6.6	4.0, 10.8	0.0	0.0, 0.4	9
**Nelson 2019[136]**	**Canada**	**2011-2013**	**SDA (BD ProbeTec)**	**0**	**86**	**Multisite**	**0.0**	**0.0, 4.3**	**0.0**	**0.0, 4.3**	**13**
**Dolling 2016[137]**	**England**	**2012-13**	**NAAT**	**12**	**251**	**Rectal**	**5.0**	**2.8, 8.2**	**6.0**	**3.7, 9.7**	**12**
Dolling 2016[137]	England	2012-13	NAAT	13	255	Pharyngeal	5.0	3.0, 8.5	0.0	0.0, 1.5	12
Dolling 2016[137]	England	2012-13	NAAT	2	256	Genital	1.0	0.2, 2.8	0.0	0.0, 1.5	12
**Closson 2018[138]**	**USA**	**2012-15**	**NAAT**	**65**	**508**	**Rectal**	**12.8**	**10.2, 16.0**	**15.6**	**12.7, 19.1**	**11**
Closson 2018[138]	USA	2012-15	NAAT	55	519	Pharyngeal	10.6	8.2, 13.5	0.0	0.0, 0.7	11
Closson 2018[138]	USA	2012-15	NAAT	31	542	Genital	5.7	4.1, 8.0	6.0	4.3, 8.3	11
**Liu 2016[139]**	**USA**	**2012-15**	**NAAT-AC2**	**55**	**556**	**Pharyngeal**	**9.9**	**7.7, 12.7**	**0.0**	**0.0, 0.7**	**12**
Liu 2016[139]	USA	2012-15	NAAT-AC2	47	554	Rectal	8.5	6.4, 11.1	10.3	8.1, 13.2	12
**Mena 2018[140]**	**USA**	**2012-15**	**NAAT-AC2**	**37**	**485**	**Rectal**	**7.6**	**5.6, 10.3**	**9.2**	**7.0, 12.1**	**13**
Mena 2018[140]	USA	2012-15	NAAT-AC2	30	485	Pharyngeal	6.2	4.4, 8.7	0.0	0.0, 0.8	13
**Ruutel 2015[141]**	**Estonia**	**2013**	**PCR**	**1**	**65**	**Genital**	**1.5**	**0.3, 8.2**	**1.3**	**0.2, 7.9**	**14**
**Tang 2020[142]**	**USA**	**2012-2014**	**AC2 NAAT**	**86**	**557**	**Multisite**	**15.4**	**12.7, 18.7**	**16.8**	**13.9, 20.1**	**11**
**Calas 2021[143]**	**France**	**2014-2015**	**FTD Urethritis Basic Kit**	**2**	**27**	**Rectal**	**7.4**	**2.1, 23.4**	**9.0**	**2.8, 25.4**	**12**
Calas 2021[143]	France	2014-2015	FTD Urethritis Basic Kit	2	53	Genital	3.8	1.0, 12.8	3.9	1.1, 12.9	12
Calas 2021[143]	France	2014-2015	FTD Urethritis Basic Kit	3	44	Pharyngeal	6.8	2.3, 18.2	0.0	0.0, 8.0	12
**Mustanski 2018[144]**	**USA**	**2013-15**	**NAAT-AC2**	**45**	**891**	**Rectal**	**5.1**	**3.8, 6.7**	**6.2**	**4.8, 7.9**	**18**
Mustanski 2018[144]	USA	2013-15	NAAT-AC2	7	893	Genital	0.8	0.4, 1.6	0.6	0.2, 1.3	18
**Achterberg 2019[145]**	**Netherlands**	**2014-2016**	**AC2 NAAT**	**89**	**1,130**	**Multisite**	**7.9**	**6.4, 9.6**	**8.4**	**7.0, 10.2**	**12**
**Achterberg 2019[146]**	**Netherlands**	**2017**	**NAAT**	**530**	**4,465**	**Multisite**	**11.9**	**11.0, 12.9**	**12.9**	**11.1, 13.9**	**12**
Achterberg 2019[146]	Netherlands	2017	NAAT	142	4,451	Genital	3.2	2.7, 3.7	3.2	2.7, 3.8	12

1. Gonorrhoea point prevalence data (continued)

Reference	Country	Year(s)	Diagnostic method	No. of MSM		Anatomical sample site	Uncorrected prevalence (%)	95% CI	Corrected prevalence (%)*	95% CI	Axis score
				Positive	Tested						
Achterberg 2019[146]	Netherlands	2017	NAAT	364	4,394	Rectal	8.3	7.5, 9.1	10.1	9.2, 11.0	12
Achterberg 2019[146]	Netherlands	2017	NAAT	256	4,429	Pharyngeal	5.9	5.1, 6.5	0.0	0.0, 0.1	12
**Ceccarani 2019[147]**	**Italy**	**2015**	**Versant CT/NG DNA assay**	**35**	**125**	**Rectal**	**28.0**	**20.9, 36.4**	**40.6**	**32.4, 49.4**	**11**
**Grov 2016[148]**	**USA**	**2014**	**Abbott Realtime Assay**	**19**	**1,071**	**Rectal**	**1.8**	**4.8, 9.4**	**2.1**	**1.4, 3.1**	**12**
**Chan 2018[149]**	**USA**	**2014-17**	**NAAT-AC2**	**24**	**415**	**Genital**	**5.8**	**3.9, 8.5**	**6.1**	**4.2, 8.8**	**12**
**Low 2017[150]**	**Switzerland**	**2015-16**	**Culture**	**117**	**230**	**Multisite**	**51**	**44.5, 57.2**	**56.4**	**49.9, 62.6**	**8**
**Wilson 2021[151]**	**UK**	**2015-2016**	**NAAT-AC2**	**52**	**509**	**Multisite**	**10.2**	**7.9, 13.2**	**11.0**	**8.6, 14.0**	**12**
Wilson 2021[151]	UK	2015-2016	NAAT-AC2	17	509	Genital	3.3	2.1, 5.3	3.3	2.1, 5.3	12
Wilson 2021[151]	UK	2015-2016	NAAT-AC2	34	509	Rectal	6.7	4.8, 9.2	8.1	6.1, 10.8	12
Wilson 2021[151]	UK	2015-2016	NAAT-AC2	34	509	Pharyngeal	6.7	4.8, 9.2	0.0	0.0, 0.7	12
**Druckler 2018[152]**	**Netherlands**	**2016**	**NAAT-AC2**	**382**	**4,925**	**Rectal**	**7.8**	**7.0, 8.5**	**9.5**	**8.7, 10.3**	**10**
Druckler 2018[152]	Netherlands	2016	NAAT-AC2	145	4,925	Genital	2.9	2.5, 3.5	2.9	2.5, 3.4	10
Druckler 2018[152]	Netherlands	2016	NAAT-AC2	329	4,925	Pharyngeal	6.7	6.0, 7.4	0.0	0.0, 0.1	10
**Salow 2017[153]**	**USA**	**2016**	**Gene Xpert NAAT**	**18**	**145**	**Rectal**	**12.4**	**8.0, 18.8**	**15.1**	**10.2, 21.9**	**11**
Salow 2017[153]	USA	2016	Gene Xpert NAAT	16	148	Pharyngeal	10.8	6.8, 16.8	0.0	0.0, 2.5	11
**Abara 2020[154]**	**USA**	**2015-2019**	**NAAT**	**23888**	**139,718**	**Multisite**	**17.1**	**16.9, 17.3**	**16.5**	**16.3, 16.7**	**12**
Abara 2020[154]	USA	2015-2019	NAAT	9703	126,972	Genital	7.6	7.5, 7.8	6.8	6.7, 7.0	12
Abara 2020[154]	USA	2015-2019	NAAT	11745	101,466	Rectal	11.6	11.4, 11.8	14.4	14.2, 14.6	12
Abara 2020[154]	USA	2015-2019	NAAT	11330	123,326	Pharyngeal	9.1	9.0, 9.3	0.0	0.0, 0.0	12
**Barbee 2021[155]**	**USA**	**2016-2018**	**AC2 NAAT**	**13**	**140**	**Rectal**	**9.3**	**5.5, 15.2**	**11.0**	**6.8, 17.2**	**14**
Barbee 2021[155]	USA	2016-2018	AC2 NAAT	2	140	Genital	1.4	0.4, 5.1	0.8	0.1, 4.0	14
Barbee 2021[155]	USA	2016-2018	AC2 NAAT	11	140	Pharyngeal	8.0	4.4, 13.5	0.0	0.0, 2.7	14
**Chapin-Bardales 2020[156]**	**USA**	**2017**	**AC2 NAAT**	**74**	**1,627**	**Pharyngeal**	**4.5**	**3.6, 5.7**	**0.0**	**0.0, 0.2**	**15**
Chapin-Bardales 2020[156]	USA	2017	AC2 NAAT	59	1,627	Rectal	3.6	2.8, 4.6	4.3	3.5, 5.4	15
**Foschi 2018[157]**	**Italy**	**2017**	**NAAT**	**45**	**165**	**Multisite**	**27.2**	**21.1, 34.5**	**29.9**	**23.4, 37.2**	**12**
Foschi 2018[157]	Italy	2017	NAAT	27	165	Genital	13.3	11.5, 22.8	16.3	11.4, 22.6	12
**Johnson-Jones 2019[158]**	**USA**	**2017**	**NAAT-AC2**	**95**	**2,072**	**Pharyngeal**	**4.6**	**3.8, 5.6**	**0.0**	**0.0, 0.2**	**13**
Johnson-Jones 2019[158]	USA	2017	NAAT-AC2	91	2,023	Rectal	4.5	3.7, 5.5	5.4	4.5, 6.5	13
**Harvey-Lavoie 2021[159]**	**Canada**	**2016-2017**	**NAAT (Cobas Amplicor)**	**79**	**1,177**	**Multisite**	**6.7**	**5.4, 8.3**	**5.9**	**4.7, 7.4**	**15**
Harvey-Lavoie 2021[159]	Canada	2016-2017	NAAT (Cobas Amplicor)	5	1,171	Genital	0.4	0.2, 1.0	0.1	0.0, 0.5	15
Harvey-Lavoie 2021[159]	Canada	2016-2017	NAAT (Cobas Amplicor)	34	1,156	Rectal	2.9	2.1, 4.1	3.7	2.8, 4.9	15
Harvey-Lavoie 2021[159]	Canada	2016-2017	NAAT (Cobas Amplicor)	55	1,175	Pharyngeal	4.7	3.6, 6.0	0.0	0.0, 0.3	15
**Fernandez-Huerta 2020[160]**	**Spain**	**2017-2018**	Gene Xpert CT/NG	**67**	**489**	**Multisite**	**13.7**	**10.9, 17.0**	**14.9**	**12.0, 18.3**	**11**
**Jansen 2020[161]**	**Germany**	**2018**	**AC2 NAAT**	**205**	**2,203**	**Multisite**	**9.3**	**8.2, 10.6**	**9.5**	**8.4, 10.8**	**15**
Jansen 2020[161]	Germany	2018	AC2 NAAT	32	2,203	Genital	1.4	1.0, 2.0	1.2	0.8, 1.8	15
Jansen 2020[161]	Germany	2018	AC2 NAAT	133	2,203	Rectal	6.0	5.1, 7.1	7.0	6.0, 8.2	15

1. Gonorrhoea point prevalence data (continued)

Reference	Country	Year(s)	Diagnostic method	No. of MSM		Anatomical sample site	Uncorrected prevalence (%)	95% CI	Corrected prevalence (%)*	95% CI	Axis score
				Positive	Tested						
Jansen 2020[161]	Germany	2018	AC2 NAAT	110	2,203	Pharyngeal	5.0	4.2, 6.0	0.0	0.0, 0.2	15
**Rahib 2022[162]**	**France**	**2018**	**Cobas Amplicor**	**186**	**1,930**	**Multisite**	**9.6**	**8.4, 11.0**	**10.3**	**9.0, 11.8**	**18**
Rahib 2022[162]	France	2018	Cobas Amplicor	10	1,930	Genital	0.5	0.3, 1.0	0.2	0.1, 0.6	18
Rahib 2022[162]	France	2018	Cobas Amplicor	84	1,930	Rectal	4.4	3.5, 5.4	5.2	4.3, 6.3	18
Rahib 2022[162]	France	2018	Cobas Amplicor	138	1,930	Pharyngeal	7.2	6.1, 8.4	0.0	0.0, 0.2	18
**Assaf 2022[163]**	**USA**	**2018-2020**	**AC2 NAAT**	**1744**	**16,189**	**Multisite**	**10.8**	**10.3, 11.3**	**11.2**	**9.2, 13.6**	**13**
**Assaf 2022$^{\chi}$[163]**	**USA**	**2018-2020**	**AC2 NAAT**	**82**	**787**	**Multisite**	**10.4**	**8.5, 12.7**	**11.6**	**11.1, 12.1**	**13**
Assaf 2022[163]	USA	2018-2020	AC2 NAAT	514	16,737	Genital	3.1	2.8, 3.3	3.1	2.8, 3.3	13
Assaf 2022[163]	USA	2018-2020	AC2 NAAT	1130	16,385	Rectal	6.9	6.5, 7.3	8.4	8.0, 8.8	13
Assaf 2022[163]	USA	2018-2020	AC2 NAAT	949	16,729	Pharyngeal	5.7	5.3, 6.0	0.0	0.0, 0.0	13
Assaf 2022$^{\chi}$[163]	USA	2018-2020	AC2 NAAT	25	821	Genital	3.1	2.8, 3.3	3.1	2.1, 4.5	13
Assaf 2022$^{\chi}$[163]	USA	2018-2020	AC2 NAAT	56	794	Rectal	7.1	5.5, 9.0	8.6	6.8, 10.7	13
Assaf 2022$^{\chi}$[163]	USA	2018-2020	AC2 NAAT	49	827	Pharyngeal	5.9	4.5, 7.7	0.0	0.0, 0.5	13
**Tyulnev 2020[164]**	**Russian Fed**	**2019-2020**	**PCR-RT**	**36**	**518**	**Rectal**	**6.9**	**5.1, 9.5**	**8.4**	**6.3, 11.1**	**5**
Tyulnev 2020[164]	Russian Fed	2019-2020	PCR-RT	9	518	Genital	1.7	0.9, 3.3	1.6	0.8, 3.0	5
Tyulnev 2020[164]	Russian Fed	2019-2020	PCR-RT	29	518	Pharyngeal	5.6	3.9, 7.9	0.0	0.0, 0.7	5

The bold rows are the included data points in the meta-analysis.
The non-bold rows are additional data points from the same paper from different anatomical sites not included in the meta-analysis due to the paper providing a multisite value or another anatomical site prevalence which was higher.
Studies where there are multiple data points included from the same paper are presented as multiple bold rows, due to different study years (documented in the year(s) column), different locations (city documented in the footnote below: $^{\nabla}$) and different populations within the same study (documented using notation which is defined in the footnote below).
* Prevalence corrected for sensitivity and specificity of diagnostic assay used
** Burkina Faso, Togo, Mali, Cote D'Ivoire
UK: United Kingdom of Great Britain and Northern Ireland
USA: United States of America
UR Tanzania: United Republic of Tanzania
$^{\lambda}$: Male sex worker cohort $^{\delta}$: Transgender female sex worker cohort $^{\chi}$: Men who have sex with men and women cohort $^{\phi}$: Transgender female cohort $^{\alpha}$: 95% MSM cohort (5% other).
$^{\varphi}$: Clinic setting $^{\theta}$: Community setting
$^{\nabla}$: Studies with multiple data points due to different population locations: Tanga, Dar Es Salaam, Francistown, Gabarone, Abuja, Lagos, Lahore, Karachi, Karnataka, Andhra Pradesh, Maharashtra, Tamil Nadu, Rawalpindi, Abbottabad, Hanoi, Ho Chi Minh, Bandung, Jakarta, Surabaya, San Miguel, San Salvador, Port Moresby, Lae and Mount Hagen.
NR: not reported. For these studies we calculated the average number of years from study period to publication amongst all included studies which was 4 years and categorised the studies where no study period was reported within the subgroup of their publication date – 4 years.
For data points from studies where the study period crossed the two time-categories (2000-2011 and 2012-2022), the midpoint of the study was used to classify the data point.
$^{\Delta}$: Multiple countries: Guatemala, Panama, Honduras, Nicaragua and El Salvador
Corrected prevalence (%): prevalence corrected for the sensitivity and specificity of the specific biological assay

2. Chlamydia point prevalence data among men who have sex with men by Sustainable Development Goal region

Reference	Country	Year(s)	Diagnostic method	No. of MSM		Anatomical sample site	Uncorrected prevalence (%)	95% CI	Corrected prevalence (%)*	95% CI	Axis score
				Positive	Tested						
**Sub-Saharan Africa**											
**Sanders 2010[9]**	**Kenya**	**NR**	**NAAT-AC2**	**5**	**43**	**Genital**	**11.6**	**5.1, 24.5**	**13.6**	**6.3, 26.8**	**9**
Sanders 2010[9]	Kenya	NR	NAAT-AC2	3	43	Rectal	7.0	2.4, 18.6	8.9	3.5, 21.1	9
**Wade 2010[10]**	**Senegal**	**2004**	**NAAT-AC2**	**17**	**419**	**Genital**	**4.1**	**2.6, 6.4**	**4.0**	**2.5, 6.3**	**12**
**Wade 2010[10]**	**Senegal**	**2007**	**NAAT-AC2**	**16**	**500**	**Genital**	**3.2**	**2.0, 5.1**	**3.0**	**1.8, 4.9**	**12**
**Vuylsteke 2012[λ,11]**	**Cote d'Ivoire**	**2007-08**	**NAAT-AC2**	**3**	**94**	**Multisite**	**3.2**	**1.1, 9.0**	**3.0**	**1.0, 8.7**	**12**
Vuylsteke 2012[λ,11]	Cote d'Ivoire	2007-08	NAAT-AC2	1	94	Rectal	1.1	0.0, 3.1	1.7	0.4, 6.8	12
Vuylsteke 2012[λ,11]	Cote d'Ivoire	2007-08	NAAT-AC2	1	94	Genital	1.1	0.0, 3.1	2.5	0.7, 8.0	12
**Kim 2016[12]**	**Uganda**	**2008**	**NAAT-AC2**	**3**	**286**	**Rectal**	**1.1**	**0.4, 3.0**	**1.2**	**0.4, 3.2**	**14**
Kim 2016[12]	Uganda	2008	NAAT-AC2	3	286	Genital	1.1	0.4, 3.0	1.2	0.4,3.2	14
**Muraguri N 2015[13]**	**Kenya**	**2010**	**NAAT-Roche Amplicor**	**12**	**290**	**Combined**	**4.1**	**2.4, 7.1**	**4.1**	**2.3, 7.0**	**13**
Muraguri N 2015[13]	Kenya	2010	NAAT-Roche Amplicor	10	290	Rectal	3.4	1.9, 6.2	4.2	2.4, 7.2	13
Muraguri N 2015[13]	Kenya	2010	NAAT-Roche Amplicor	2	290	Genital	0.69	0.2, 2.5	0.0	0.0, 1.3	13
**Sanders 2014[14]**	**Kenya**	**2011**	**NAAT-AC2**	**21**	**244**	**Multisite**	**8.6**	**5.9, 12.7**	**10.5**	**7.2, 15.0**	**11**
Sanders 2014[14]	Kenya	2011	NAAT-AC2	20	244	Genital	8.2	5.4, 12.3	10.5	7.2, 15.0	11
Sanders 2014[14]	Kenya	2011	NAAT-AC2	15	244	Rectal	6.1	3.8, 9.9	6.5	4.1, 10.4	11
**Rebe 2015[15]**	**South Africa**	**2012**	**NAAT-AC2**	**16**	**200**	**Rectal**	**8.0**	**5.0, 12.6**	**10.2**	**6.7, 15.2**	**10**
Rebe 2015[15]	South Africa	2012	NAAT-AC2	7	200	Genital	3.5	1.7, 7.0	3.4	1.6, 6.9	10
Rebe 2015[15]	South Africa	2012	NAAT-AC2	0	200	Pharyngeal	0.0	0.0, 1.9	0.0	0.0, 1.9	10
**Ross 2014[16]**	**UR Tanzania[∇]**	**2012**	**NAAT-AC2**	**3**	**40**	**Genital**	**7.5**	**2.6, 19.9**	**8.2**	**2.9, 20.7**	**11**
**Ross 2014[16]**	**UR Tanzania[∇]**	**2012**	**NAAT-AC2**	**23**	**180**	**Rectal**	**12.8**	**8.7, 18.4**	**16.5**	**11.8, 22.6**	**11**
Ross 2014[16]	UR Tanzania	2012	NAAT-AC2	4	179	Genital	2.2	0.9, 5.6	1.8	0.6, 5.0	11
Ross 2014[16]	UR Tanzania	2012	NAAT-AC2	1	40	Rectal	2.5	0.4, 12.9	3.0	0.6, 13.7	11
**Tafuma 2014[17]**	**Botswana[∇]**	**2012**	**PCR**	**24**	**275**	**Rectal**	**8.6**	**5.9, 12.7**	**11.0**	**7.8, 15.3**	**11**
**Tafuma 2014[17]**	**Botswana[∇]**	**2012**	**PCR**	**17**	**145**	**Genital**	**11.5**	**7.5, 18.0**	**13.0**	**8.4, 19.4**	**11**
Tafuma 2014[17]	Botswana	2012	PCR	16	275	Genital	5.9	3.6, 9.2	6.2	3.9, 9.7	11
Tafuma 2014[17]	Botswana	2012	PCR	4	145	Rectal	3.1	1.1, 6.9	3.8	1.7, 8.3	11
**Venter 2019[18]**	**South Africa**	**2011-2012**	**PCR**	**15**	**199**	**Rectal**	**7.5**	**4.6, 12.1**	**9.6**	**6.3, 14.5**	**12**
Venter 2019[18]	South Africa	2011-2012	PCR	7	200	Genital	3.5	1.7, 7.0	3.4	1.6, 6.9	12
Venter 2019[18]	South Africa	2011-2012	PCR	0	194	Pharnygeal	0.0	0.0, 1.9	0.0	0.0, 1.9	12
**Kayode 2020[19]**	**Nigeria**	**2013**	**PCR**	**235**	**1,447**	**Multisite**	**16.4**	**14.4, 18.2**	**18.8**	**16.9, 20.9**	**12**
**Keshinro 2016[20]**	**Nigeria[∇]**	**2013-16**	**NAAT-AC2**	**80**	**546**	**Multisite**	**14.7**	**11.9, 17.9**	**16.8**	**13.9, 20.2**	**14**
**Keshinro 2016[20]**	**Nigeria[∇]**	**2013-16**	**NAAT-AC2**	**61**	**316**	**Multisite**	**19.3**	**15.3, 24.0**	**22.3**	**18.1, 27.2**	**14**
Keshinro 2016[20]	Nigeria	2013-16	NAAT-AC2	NR	546	Genital	4.6	3.1, 6.6	4.7	3.2, 6.8	14

Keshinro 2016[20]	Nigeria	2013-16	NAAT-AC2	NR	546	Rectal	11.7	9.2, 14.7	15.1	12.3, 18.3	14

2. Chlamydia point prevalence data (continued)

Reference	Country	Year(s)	Diagnostic method	No. of MSM		Anatomical sample site	Uncorrected prevalence (%)	95% CI	Corrected prevalence (%)*	95% CI	Axis score
				Positive	Tested						
Keshinro 2016[20]	Nigeria	2013-16	NAAT-AC2	NR	316	Genital	5.4	3.4, 8.5	5.6	3.6, 8.8	14
Keshinro 2016[20]	Nigeria	2013-16	NAAT-AC2	NR	316	Rectal	16.5	12.8, 21.0	21.4	17.2, 26.2	14
**De Baetelier 2020[21]**	**B. Faso, Togo, Mali, CID****	**2016**	**Abbott RealTime PCR**	**72**	**497**	**Multisite**	**14.5**	**11.7, 17.9**	**16.6**	**13.6, 20.1**	**9**
**Jones 2020[22]**	**South Africa**	**2015-2016**	**GeneXpert CT/NG**	**47**	**189**	**Rectal**	**24.9**	**19.2, 31.5**	**32.4**	**26.1 39.3**	**11**
Jones 2020[22]	South Africa	2015-2016	GeneXpert CT/NG	29	270	Genital	10.7	7.6, 15.0	11.2	7.9, 15.5	11
**Ngetsa 2020[23]**	**Kenya**	**2016-2017**	**GeneXpert CT/NG**	**14**	**104**	**Rectal**	**13.5**	**8.2, 21.3**	**17.4**	**11.3, 25.8**	**16**
**Otieno 2020[24]**	**Kenya**	**2015-2016**	**Cobas Amplicor CT/NG**	**61**	**619**	**Genital**	**9.9**	**7.7, 12.5**	**11.0**	**8.8, 13.8**	**19**
Otieno 2020[24]	Kenya	2015-2016	Cobas Amplicor CT/NG	19	619	Rectal	3.1	2.0, 4.7	3.8	2.6, 5.6	19
**Ferre 2019[25]**	**Togo**	**2017**	**Allplex STI Essential Assay**	**20**	**207**	**Rectal**	**9.7**	**6.3, 14.5**	**12.5**	**8.6, 17.6**	**12**
**Twahirwa 2021[26]**	**Rwanda**	**2018**	**GeneXpert CT/NG**	**67**	**737**	**Multisite**	**9.1**	**7.2, 11.4**	**10.1**	**8.1, 12.5**	**11**
**Northern Africa and Western Asia**											
**Mor 2012[27]**	**Israel**	**2002-08**	**PCR**	**23**	**1,064**	**Genital**	**2.2**	**1.4, 3.2**	**1.8**	**1.2, 2.8**	**10**
**Hancali 2019[28]**	**Morocco**	**2017**	**GeneXpert CT/NG**	**22**	**238**	**Rectal**	**9.2**	**6.2, 13.6**	**11.8**	**8.3, 16.6**	**8**
**Zucker 2022[29]**	**Israel**	**2019-2020**	**GeneXpert CT/NG**	**29**	**210**	**Rectal**	**13.8**	**9.8, 19.1**	**17.8**	**13.2, 23.6**	**13**
Zucker 2022[29]	Israel	2019-2020	GeneXpert CT/NG	2	210	Genital	1.0	0.3, 3.4	1.6	0.6, 4.3	13
Zucker 2022[29]	Israel	2019-2020	GeneXpert CT/NG	6	210	Pharyngeal	2.9	1.3, 6.1	8.0	5.0, 12.4	13
**Central and Southern Asia**											
**Rehan 2009[λ30]**	**India[∇]**	**2004**	**PCR**	**6**	**395**	**Genital**	**1.5**	**0.7, 3.3**	**0.8**	**0.3, 2.3**	**13**
**Rehan 2009[λ30]**	**India[∇]**	**2004**	**PCR**	**4**	**394**	**Rectal**	**1.0**	**0.4, 2.6**	**1.1**	**0.4, 2.7**	**13**
Rehan 2009[λ30]	India	2004	PCR	5	395	Genital	1.2	0.5, 2.9	0.5	0.1, 1.8	13
**Brahmam 2008[31]**	**India[∇]**	**2006-2007**	**NAAT**	**5**	**298**	**Genital**	**1.6**	**0.7, 3.9**	**1.1**	**0.4, 3.0**	**14**
**Brahmam 2008[31]**	**India[∇]**	**2006-07**	**NAAT**	**26**	**1,621**	**Genital**	**1.6**	**1.1, 2.3**	**1.1**	**0.7, 1.7**	**14**
**Brahmam 2008[31]**	**India[∇]**	**2006-07**	**NAAT**	**26**	**653**	**Genital**	**4.0**	**2.7, 5.8**	**4.0**	**2.7, 5.7**	**14**
**Brahmam 2008[31]**	**India[∇]**	**2006-07**	**NAAT**	**12**	**2025**	**Genital**	**0.6**	**0.3, 1.0**	**0.0**	**0.0, 0.2**	**14**
**Hawkes 2009[λ32]**	**Pakistan[∇]**	**2007**	**NAAT-Roche Amplicor PCR**	**22**	**559**	**Rectal**	**3.9**	**2.6, 5.9**	**4.8**	**3.4, 7.0**	**12**
**Hawkes 2009[λ32]**	**Pakistan[∇]**	**2007**	**NAAT-Roche Amplicor PCR**	**1**	**16**	**Rectal**	**6.3**	**1.1, 28.3**	**8.0**	**1.7, 30.6**	**12**
**Hawkes 2009[δ32]**	**Pakistan[∇]**	**2007**	**NAAT-Roche Amplicor PCR**	**4**	**87**	**Rectal**	**4.6**	**1.8, 11.2**	**5.8**	**2.5, 12.8**	**12**
**Hawkes 2009[λ32]**	**Pakistan[∇]**	**2007**	**NAAT-Roche Amplicor PCR**	**25**	**253**	**Rectal**	**9.9**	**6.8, 14.2**	**12.7**	**9.2, 17.4**	**12**
Hawkes 2009[λ32]	Pakistan	2007	NAAT-Roche Amplicor PCR	0	559	Genital	0.0	0.0, 0.7	0.0	0.0, 0.7	12
Hawkes 2009[δ32]	Pakistan	2007	NAAT-Roche Amplicor PCR	0	16	Genital	0.0	0.0, 19.4	0.0	0.0, 19.4	12
Hawkes 2009[λ32]	Pakistan	2007	NAAT-Roche Amplicor PCR	1	87	Genital	1.1	0.2, 6.2	0.5	0.0, 5.1	12
Hawkes 2009[δ32]	Pakistan	2007	NAAT-Roche Amplicor PCR	0	253	Genital	0.0	0.0, 1.5	0.0	0.0 1.5	12
**Safren, 2020[35]**	**India**	**2016**	**Abbott RealTime PCR**	**88**	**608**	**Multisite**	**14.5**	**11.9, 17.5**	**16.8**	**14.0, 20.0**	**17**

2. Chlamydia point prevalence data (continued)

Reference	Country	Year(s)	Diagnostic method	No. of MSM		Anatomical sample site	Uncorrected prevalence (%)	95% CI	Corrected prevalence (%)*	95% CI	Axis score
				Positive	Tested						
**Eastern and South-Eastern Asia**											
**Chau 2016[36]**	**Hong Kong SAR**	**NR**	**AC2 NAAT**	**18**	**158**	**Rectal**	**11.4**	**7.3, 17.3**	**14.7**	**10.0, 21.0**	**11**
Chau 2016[36]	Hong Kong SAR	NR	AC2 NAAT	7	158	Genital	4.4	2.2, 8.9	4.4	2.2, 8.9	11
Chau 2016[36]	Hong Kong SAR	NR	AC2 NAAT	4	158	Pharyngeal	2.5	1.0, 6.3	2.8	1.2, 6.8	11
**Wei 2009[37]**	**China**	**1999-2000**	**LCR**	**3**	**38**	**Genital**	**7.9**	**2.7, 20.8**	**8.0**	**2.8, 20.9**	**12**
**Girault 2004[38]**	**Cambodia**	**2000**	**NAAT-Cobas Amplicor**	**3**	**206**	**Rectal**	**1.0**	**0.5, 4.2**	**1.0**	**0.3, 3.6**	**14**
**Jiang 2006[39]**	**China**	**2003**	**NAAT-Roche Amplicor**	**9**	**122**	**Genital**	**8.0**	**3.9, 13.4**	**8.3**	**4.6, 14.6**	**11**
**Hoang 2006[40]**	**Vietnam[∇]**	**2005-06**	**PCR**	**30**	**397**	**Genital**	**7.6**	**5.3, 10.6**	**8.3**	**6.0, 11.4**	**15**
**Hoang 2006[40]**	**Vietnam[∇]**	**2005-06**	**PCR**	**20**	**393**	**Genital**	**5.0**	**3.3, 7.7**	**5.2**	**3.4, 7.8**	**15**
Hoang 2006[40]	Vietnam	2005-06	PCR	21	397	Rectal	5.4	3.5, 8.0	6.8	4.7, 9.7	15
Hoang 2006[40]	Vietnam	2005-06	PCR	15	393	Rectal	3.8	2.3, 6.2	4.7	3.0, 7.3	15
**Pattanasin 2018[41]**	**Thailand**	**2006-10**	**NAAT-Roche Amplicor**	**243**	**1,695**	**Multisite**	**14.3**	**12.8, 16.1**	**16.3**	**14.6, 18.2**	**14**
Pattanasin 2018[41]	Thailand	2006-10	NAAT-Roche Amplicor	53	1,695	Genital	3.1	2.4, 4.1	2.9	2.2, 3.8	14
Pattanasin 2018[41]	Thailand	2006-10	NAAT-Roche Amplicor	34	1,689	Pharyngeal	2.0	1.4, 2.8	2.3	1.7, 3.1	14
Pattanasin 2018[41]	Thailand	2006-10	NAAT-Roche Amplicor	110	1,552	Rectal	8.0	5.9, 8.5	10.2	8.8, 11.8	14
**Tongtoyai 2015[42]**	**Thailand**	**2006-10**	**NAAT-Roche Amplicor**	**151**	**1,596**	**Rectal**	**9.5**	**8.1, 11.0**	**12.2**	**10.7, 13.9**	**14**
Tongtoyai 2015[42]	Thailand	2006-10	NAAT-Roche Amplicor	78	1,743	Genital	4.5	3.6, 5.6	4.6	3.7, 5.6	14
Tongtoyai 2015[42]	Thailand	2006-10	NAAT-Roche Amplicor	63	1,743	Phalangeal	3.6	2.8, 4.6	4.1	3.3, 5.1	14
**Morineau 2011[44]**	**Indonesia[∇]**	**2007**	**NAAT-Cobas Amplicor**	**48**	**246**	**Rectal**	**19.5**	**15.0, 24.9**	**25.3**	**20.3, 31.1**	**12**
**Morineau 2011[44]**	**Indonesia[∇]**	**2007**	**NAAT-Cobas Amplicor**	**53**	**242**	**Rectal**	**21.9**	**17.2, 27.5**	**28.4**	**23.1, 34.4**	**12**
**Morineau 2011[44]**	**Indonesia[∇]**	**2007**	**NAAT-Cobas Amplicor**	**53**	**249**	**Rectal**	**21.3**	**16.7, 26.8**	**27.7**	**22.5, 33.5**	**12**
Morineau 2011[44]	Indonesia	2007	NAAT-Cobas Amplicor	11	250	Genital	4.4	2.5, 7.7	4.4	2.5, 7.8	12
Morineau 2011[44]	Indonesia	2007	NAAT-Cobas Amplicor	17	250	genital	6.8	4.3, 10.6	7.3	4.7, 11.2	12
Morineau 2011[44]	Indonesia	2007	NAAT-Cobas Amplicor	9	249	Genital	3.6	1.9, 6.7	3.5	1.8, 6.6	12
**Prabawanti 2011[45]**	**Indonesia**	**2007**	**NAAT-Roche Amplicor**	**223**	**734**	**Rectal**	**30.4**	**27.2, 33.8**	**39.6**	**36.1, 43.2**	**10**
**Jung 2012[46]**	**Rep of Korea**	**2008**	**PCR**	**2**	**106**	**Genital**	**1.9**	**0.5, 6.6**	**1.4**	**0.3, 5.9**	**10**
**Fu 2015[47]**	**China**	**2009**	**NAAT-Roche Amplicor**	**27**	**413**	**Genital**	**6.5**	**4.5, 9.3**	**7.0**	**4.9, 9.9**	**10**
**Pham 2012[48]**	**Vietnam**	**2009**	**NAAT-Roche Amplicor**	**12**	**381**	**Genital**	**3.2**	**1.8, 5.4**	**3.0**	**1.7, 5.2**	**15**
**Wong 2013[58]**	**Hong Kong SAR**	**2011**	**NAAT-AC2**	**47**	**994**	**Genital**	**4.7**	**3.6, 6.2**	**4.8**	**3.6, 6.3**	**14**
**Wickersham 2017[δ 49]**	**Malaysia**	**2014**	**NAAT-AC2**	**5**	**186**	**Genital**	**2.7**	**1.2, 6.1**	**2.4**	**1.0, 5.7**	**15**
**Hinkan 2018[166]**	**Thailand**	**2014-15**	**RT-PCR**	**101**	**346**	**Genital**	**29.1**	**24.7, 34.2**	**32.6**	**27.9, 37.7**	**10**
Hinkan 2018[166]	Thailand	2014-15	RT-PCR	61	346	Pharyngeal	17.6	14.0, 22.0	20.0	16.1, 24.5	10
Hinkan 2018[166]	Thailand	2014-15	RT-PCR	59	346	Rectal	17.0	13.5, 21.4	22.0	18.0, 26.7	10
**Chen, 2021[51]**	**Vietnam**	**2016**	**Abbott RealTime PCR**	**39**	**205**	**Multisite**	**19.0**	**14.2, 24.9**	**22.0**	**16.8, 28.1**	**15**

Chen, 2021[51]	Vietnam	2016	Abbott RealTime PCR	14	205	Genital	6.8	4.1, 11.1	7.6	4.7, 12.0	15

2. Chlamydia point prevalence data (continued)

Reference	Country	Year(s)	Diagnostic method	No. of MSM		Anatomical sample site	Uncorrected prevalence (%)	95% CI	Corrected prevalence (%)*	95% CI	Axis score
				Positive	Tested						
Chen, 2021[51]	Vietnam	2016	Abbott RealTime PCR	27	205	Rectal	13.2	9.2, 18.5	16.8	12.3, 22.5	15
Chen, 2021[51]	Vietnam	2016	Abbott RealTime PCR	10	205	Pharyngeal	4.9	2.7, 8.7	5.7	3.3, 9.7	15
**Hiransuthikul, 2019[52]**	**Thailand**	**2015-2016**	**Abbott RealTime PCR**	**349**	**1,610**	**Multisite**	**21.7**	**19.7, 23.8**	**25.2**	**23.1, 27.4**	**13**
Hiransuthikul, 2019[52]	Thailand	2015-2016	Abbott RealTime PCR	112	1,610	Genital	7.0	5.8, 8.3	7.6	6.4, 9.0	13
Hiransuthikul, 2019[52]	Thailand	2015-2016	Abbott RealTime PCR	242	1,610	Rectal	15.0	13.4, 16.9	19.4	17.5, 21.4	13
Hiransuthikul, 2019[52]	Thailand	2015-2016	Abbott RealTime PCR	48	1,610	Pharyngeal	3.0	2.3, 3.9	3.4	2.6, 4.4	13
**Yang 2018[φ][53]**	**China**	**2015-17**	**NAAT-Roche Amplicor**	**29**	**153**	**Multisite**	**19.0**	**13.5, 25.9**	**22.0**	**16.1, 29.2**	**14**
**Yang 2018[θ][53]**	**China**	**2015-17**	**NAAT-Roche Amplicor**	**55**	**310**	**Multisite**	**17.7**	**13.9, 22.4**	**20.4**	**16.3, 25.2**	**14**
Yang 2018[φ][53]	China	2015-17	NAAT-Roche Amplicor	18	153	Genital	11.8	7.6, 17.8	13.3	8.8, 19.6	14
Yang 2018[φ][53]	China	2015-17	NAAT-Roche Amplicor	10	153	Rectal	6.5	3.6, 11.6	8.3	4.9, 13.7	14
Yang 2018[φ][53]	China	2015-17	NAAT-Roche Amplicor	1	153	Pharyngeal	0.7	0.1, 3.6	0.8	0.2, 3.8	14
Yang 2018[θ][53]	China	2015-17	NAAT-Roche Amplicor	13	310	Genital	4.2	2.5, 7.0	4.2	2.5, 7.1	14
Yang 2018[θ][53]	China	2015-17	NAAT-Roche Amplicor	42	310	Rectal	13.6	10.2, 17.8	17.6	13.7, 22.2	14
Yang 2018[θ][53]	China	2015-17	NAAT-Roche Amplicor	5	310	Pharyngeal	1.6	0.7, 3.7	1.8	0.8, 4.0	14
**Adamson, 2022[54]**	**Vietnam**	**2017-2019**	**Cobas Amplicor PCR**	**322**	**1,489**	**Multisite**	**21.6**	**19.6, 23.8**	**25.1**	**23.0, 27.4**	**14**
Adamson, 2022[54]	Vietnam	2017-2019	Cobas Amplicor PCR	61	1,489	Genital	4.1	3.2, 5.3	4.1	3.2, 5.2	14
Adamson, 2022[54]	Vietnam	2017-2019	Cobas Amplicor PCR	163	1,489	Rectal	11.5	9.9, 13.3	14.8	13.1, 16.8	14
Adamson, 2022[54]	Vietnam	2017-2019	Cobas Amplicor PCR	21	1,489	Pharyngeal	1.4	0.9, 2.2	1.6	1.1, 2.4	14
**Songtaweesin, 2022[55]**	**Thailand**	**2018-2019**	**Abbott RealTime PCR**	**30**	**200**	**Multisite**	**15.0**	**10.7, 20.6**	**17.2**	**12.6, 23.0**	**11**
Songtaweesin, 2022[55]	Thailand	2018-2019	Abbott RealTime PCR	7	200	Genital	3.5	1.7, 7.0	3.4	1.6, 6.9	11
Songtaweesin, 2022[55]	Thailand	2018-2019	Abbott RealTime PCR	28	200	Rectal	14.0	9.9, 19.5	18.1	13.4, 24.0	11
**Zhou, 2019[56]**	**China**	**2017-2018**	**Cobas Amplicor PCR**	**69**	**379**	**Multisite**	**18.2**	**14.6, 22.4**	**21.0**	**17.2, 25.4**	**17**
Zhou, 2019[56]	China	2017-2018	Cobas Amplicor PCR	12	379	Genital	3.2	1.8, 5.5	3.0	1.7, 5.2	17
Zhou, 2019[56]	China	2017-2018	Cobas Amplicor PCR	59	379	Rectal	15.6	12.3, 19.6	20.2	16.5 24.5	17
Zhou, 2019[56]	China	2017-2018	Cobas Amplicor PCR	6	379	Pharyngeal	1.6	0.7, 3.4	1.8	0.9, 3.7	17
**Latin American and the Caribbean**											
**Clark 2007[57]**	**Peru**	**2000-2001**	**NAAT-Roche Amplicor**	**5**	**85**	**Genital**	**6.0**	**2.5, 13.0**	**6.4**	**2.8, 13.7**	**13**
**Soto 2007[60]**	**Multiple[Δ]**	**2001-2002**	**Cobas Amplicor PCR**	**101**	**1,409**	**Genital**	**7.2**	**5.9, 8.6**	**7.8**	**6.5, 9.3**	**12**
**Clark 2009[61]**	**Peru**	**2003**	**NAAT-Roche Amplicor**	**5**	**541**	**Genital**	**1.1**	**0.4, 2.2**	**0.5**	**0.2, 1.5**	**13**
**Konda 2013[χ][62]**	**Peru**	**2003-2007**	**NAAT-Roche Amplicor**	**14**	**193**	**Genital**	**7.3**	**4.4, 11.8**	**7.9**	**4.9, 12.6**	**12**
**Konda 2013[62]**	**Peru**	**2003-2007**	**NAAT-Roche Amplicor**	**2**	**75**	**Genital**	**2.7**	**0.7, 9.2**	**2.4**	**0.6, 8.8**	**12**
Konda 2013[χ][62]	Peru	2003-2007	NAAT-Roche Amplicor	14	312	Pharyngeal	4.5	2.7, 7.4	5.1	3.2, 8.1	12
Konda 2013[χ][62]	Peru	2003-2007	NAAT-Roche Amplicor	43	312	Rectal	14.1	10.4, 18.1	18.2	14.3, 22.9	12

2. Chlamydia point prevalence data (continued)

Reference	Country	Year(s)	Diagnostic method	No. of MSM		Anatomical sample site	Uncorrected prevalence (%)	95% CI	Corrected prevalence (%)*	95% CI	Axis score
				Positive	Tested						
**Dos Ramos Farias 2011[δ167]**	**Argentina**	**2006-2009**	**PCR**	**4**	**80**	**Rectal**	**5.0**	**2.0, 12.2**	**6.3**	**2.7, 13.9**	**11**
**Perez-Brumer 2013[64]**	**Peru**	**2007**	**NAAT-Roche Amplicor**	**23**	**560**	**Genital**	**4.1**	**2.8, 6.1**	**4.1**	**2.7, 6.1**	**13**
**Figueroa 2013[65]**	**Jamaica**	**2007-2008**	**NAAT-AC2**	**22**	**201**	**Genital**	**11**	**7.3, 16.0**	**12.4**	**8.5, 17.6**	**10**
**Pando 2012[χ168]**	**Brazil**	**2007-2009**	**PCR**	**0**	**25**	**Rectal**	**0.0**	**0.0, 13.3**	**0.0**	**0.0, 13.3**	**12**
**Pando 2012[168]**	**Brazil**	**2007-2009**	**PCR**	**2**	**70**	**Rectal**	**2.9**	**0.8, 9.8**	**3.5**	**1.1, 10.8**	**12**
**Creswell 2012[66]**	**El Salvador[∇]**	**2008**	**PCR**	**8**	**110**	**Rectal**	**7.3**	**3.7, 13.7**	**9.3**	**5.2, 16.2**	**15**
**Creswell 2012[66]**	**El Salvador[∇]**	**2008**	**PCR**	**12**	**460**	**Genital**	**2.6**	**1.5, 4.5**	**2.3**	**1.3, 4.1**	**15**
Creswell 2012[66]	El Salvador	2008	PCR	8	390	Rectal	2.1	1.0, 4.0	2.5	1.3, 4.6	15
Creswell 2012[66]	El Salvador	2008	PCR	5	188	Genital	2.7	1.1, 6.1	2.4	1.0, 5.7	15
**Leon 2013[67]**	**Peru**	**2008**	**NAAT-AC2**	**136**	**718**	**Rectal**	**19**	**16.2, 22.0**	**24.6**	**21.6, 27.9**	**7**
Leon 2013[67]	Peru	2008	NAAT-AC2	34	718	Pharyngeal	4.8	3.4, 6.5	5.5	4.0, 7.4	7
**Castillo 2015[68]**	**Peru**	**2009**	**NAAT-AC2**	**135**	**713**	**Genital**	**19**	**16.2, 22.0**	**24.6**	**21.6, 27.9**	**12**
**Cunha 2015[69]**	**Brazil**	**2010-2012**	**NAAT-AC2**	**28**	**279**	**Rectal**	**10**	**7.0, 14.1**	**12.8**	**9.4, 17.3**	**14**
Cunha 2015[69]	Brazil	2010-2012	NAAT-AC2	6	273	Genital	2.2	1.0, 4.7	1.8	0.8, 4.2	14
**Allan Blitz 2017[70]**	**Peru**	**2010-2014**	**NAAT-AC2**	**53**	**387**	**Rectal**	**13.7**	**10.6, 17.5**	**17.7**	**14.2, 21.8**	**12**
Allan Blitz 2017[70]	Peru	2010-2014	NAAT-AC2	22	387	Pharyngeal	5.7	3.8, 8.5	6.5	4.4, 9.4	12
**Galarraga 2014[71]**	**Mexico**	**2012**	**NAAT-AC2**	**26**	**267**	**Genital**	**9.81**	**6.7, 13.9**	**10.9**	**7.7, 15.2**	**14**
**Morales-Miranda 2013[72]**	**Belize**	**2012**	**PCR**	**9**	**130**	**Genital**	**6.9**	**3.7, 12.6**	**7.0**	**3.8, 12.8**	**8**
Morales-Miranda 2013[72]	Belize	2012	PCR	6	130	Rectal	4.3	2.1, 9.7	5.4	2.6, 10.7	8
**Kojima 2017[73]**	**Peru**	**2012-13**	**NAAT-AC2**	**57**	**312**	**Rectal**	**18.6**	**14.4, 22.9**	**21.1**	**17.0, 26.0**	**14**
**Cabeza 2015[74]**	**Peru**	**2012-14**	**NAAT-AC2**	**130**	**834**	**Rectal**	**15.6**	**13.3, 18.2**	**20.2**	**17.6, 23.0**	**9**
Cabeza 2015[74]	Peru	2012-14	NAAT-AC2	38	834	Pharyngeal	4.5	3.3, 6.2	5.1	3.8, 6.8	9
Cabeza 2015[74]	Peru	2012-14	NAAT-AC2	29	834	Genital	3.5	2.4, 5.0	3.4	2.3, 4.8	9
**Passaro 2018[75]**	**Peru**	**2012-14**	**NAAT-AC2**	**65**	**787**	**Rectal**	**8.3**	**6.5, 10.4**	**10.6**	**8.7, 13.0**	**11**
Passaro 2018[75]	Peru	2012-14	NAAT-AC2	31	787	Pharyngeal	3.9	2.8, 5.5	4.4	3.2 6.1	11
Passaro 2018[75]	Peru	2012-14	NAAT-AC2	28	787	Genital	3.6	2.5, 5.1	3.5	2.4, 5.0	11
**Chow 2017[76]**	**Peru**	**2013-14**	**NAAT-AC2**	**43**	**312**	**Rectal**	**13.8**	**10.4, 18.1**	**17.8**	**14.0, 22.5**	**11**
**Mendizabal-Burastero 2015[77]**	**Guatemala**	**2014**	**PCR-Digene Hybrid Capture**	**25**	**524**	**Rectal**	**4.7**	**3.3, 7.0**	**5.9**	**4.2, 8.3**	**7**
Mendizabal-Burastero 2015[77]	Guatemala	2014	PCR-Digene Hybrid Capture	10	524	Genital	1.9	1.0, 3.5	1.3	0.6, 2.6	7
Mendizabal-Burastero 2015[77]	Guatemala	2014	PCR-Digene Hybrid Capture	12	524	Pharyngeal	2.2	1.3, 4.0	2.5	1.5, 4.2	7
**Grinsztejn 2017[ϕ78]**	**Brazil**	**2015**	**PCR**	**46**	**345**	**Rectal**	**14.1**	**10.2, 17.3**	**18.2**	**14.5, 22.6**	**17**

2. Chlamydia point prevalence data (continued)

Reference	Country	Year(s)	Diagnostic method	No. of MSM		Anatomical sample site	Uncorrected prevalence (%)	95% CI	Corrected prevalence (%)*	95% CI	Axis score
				Positive	Tested						
**Bristow, 2021[79]**	**Mexico**	**2017-2018**	**GeneXpert CT/NG**	**41**	**212**	**Multisite**	**19.3**	**14.6, 25.2**	**22.3**	**17.2, 28.4**	**14**
Bristow, 2021[79]	Mexico	2017-2018	GeneXpert CT/NG	16	212	Genital	7.5	4.7, 11.9	8.2	5.2, 12.6	14
Bristow, 2021[79]	Mexico	2017-2018	GeneXpert CT/NG	25	212	Rectal	11.8	8.1, 16.8	15.2	11.0, 20.7	14
Bristow, 2021[79]	Mexico	2017-2018	GeneXpert CT/NG	6	212	Pharyngeal	2.8	1.3, 6.0	3.2	1.5, 6.5	14
**Moriarty, 2019[80]**	**Peru**	**2017**	**Gen-Probe Aptima**	**32**	**120**	**Rectal**	**26.7**	**19.6, 35.2**	**34.7**	**26.8, 43.6**	**15**
**Jean Louis, 2020[81]**	**Haiti**	**2018-2019**	**GeneXpert CT/NG**	**24**	**216**	**Multisite**	**11.1**	**7.6, 16.0**	**12.5**	**8.7, 17.6**	**16**
Jean Louis, 2020[81]	Haiti	2018-2019	GeneXpert CT/NG	8	216	Genital	3.7	1.9, 7.1	3.6	1.8, 7.0	16
Jean Louis, 2020[81]	Haiti	2018-2019	GeneXpert CT/NG	17	216	Rectal	7.9	5.0, 12.2	10.1	6.7, 14.8	16
**Australia and New Zealand**											
**Lister 2004[82]**	**Australia**	**2001**	**SDA (BD ProbeTec)**	**5**	**34**	**Rectal**	**14.7**	**6.5, 30.1**	**19.2**	**9.4, 35.3**	**11**
Lister 2004[82]	Australia	2001	SDA (BD ProbeTec)	0	30	Pharyngeal	0.0	0.0, 11.4	0.0	0.0, 11.4	11
Lister 2004[82]	Australia	2001	SDA (BD ProbeTec)	4	80	Genital	5.0	2.0, 12.2	1.5	0.3, 7.1	11
Lister 2004[82]	Australia	2001	SDA (BD ProbeTec)	7	303	Genital	2.3	1.1, 4.7	0.0	0.0, 1.3	11
Lister 2004[82]	Australia	2001	SDA (BD ProbeTec)	10	185	Rectal	5.4	3.0, 9.7	7.1	4.2, 11.7	11
Lister 2004[82]	Australia	2001	SDA (BD ProbeTec)	1	220	Pharyngeal	0.5	0.1, 2.5	1.5	0.5, 4.1	11
**Lister 2003[83]**	**Australia**	**2001-02**	**NAAT-cobas amplicor**	**30**	**507**	**Rectal**	**5.9**	**4.2, 8.3**	**7.5**	**5.5, 10.1**	**14**
Lister 2003[83]	Australia	2001-02	NAAT-cobas amplicor	9	511	Genital	1.8	0.9, 3.3	1.2	0.5, 2.5	14
Lister 2003[83]	Australia	2001-02	NAAT-cobas amplicor	3	521	Pharyngeal	0.6	0.2, 1.7	0.7	0.3, 1.8	14
**Hamlyn 2006[84]**	**Australia**	**2001-04**	**SDA (BD ProbeTec)**	**11**	**253**	**Rectal**	**4.5**	**2.4, 7.6**	**5.9**	**3.6, 9.5**	**13**
Hamlyn 2006[84]	Australia	2001-04	SDA (BD ProbeTec)	1	253	Genital	0.5	0.1, 2.2	0.0	0.0, 1.5	13
Hamlyn 2006[84]	Australia	2001-04	SDA (BD ProbeTec)	2	253	Pharyngeal	1.0	0.2, 2.8	3.0	1.5, 5.9	13
**Jin 2007[85]**	**Australia**	**2001-06**	**SDA**	**65**	**1,408**	**Rectal**	**4.6**	**3.6, 5.8**	**6.0**	**4.9, 7.4**	**14**
Jin 2007[85]	Australia	2001-06	SDA	16	1,417	Genital	1.1	0.7, 1.8	0.0	0.0, 0.3	14
**Templeton 2008[169]**	**Australia**	**2001-07**	**SDA (BD ProbeTec)**	**13**	**1,227**	**Pharyngeal**	**1.1**	**0.6, 1.8**	**3.2**	**2.3, 4.3**	**14**
**Lister 2005[86]**	**Australia**	**2002**	**SDA (BD ProbeTec)**	**10**	**185**	**Rectal**	**5.4**	**3.0, 9.7**	**7.1**	**4.2, 11.7**	**14**
Lister 2005[86]	Australia	2002	SDA (BD ProbeTec)	7	303	Genital	2.3	1.1, 4.7	0.0	0.0, 1.3	14
Lister 2005[86]	Australia	2002	SDA (BD ProbeTec)	1	220	Pharyngeal	0.5	0.1 2.5	1.5	0.5, 4.0	14
**Guy 2011[170]**	**Australia**	**2004**	**NAAT**	**202**	**2,295**	**Genital**	**8.8**	**7.7, 10.0**	**9.7**	**8.6, 11.0**	**13**
**Guy 2011[170]**	**Australia**	**2005**	**NAAT**	**198**	**2,352**	**Genital**	**8.4**	**7.4, 9.6**	**9.2**	**8.1, 10.5**	**13**
**Guy 2011[170]**	**Australia**	**2006**	**NAAT**	**198**	**2,272**	**Genital**	**8.7**	**7.6, 10.0**	**9.6**	**8.5, 10.9**	**13**
**Guy 2011[170]**	**Australia**	**2007**	**NAAT**	**191**	**2,415**	**Genital**	**7.9**	**6.9, 9.1**	**8.6**	**7.6, 9.8**	**13**
**Guy 2011[170]**	**Australia**	**2008**	**NAAT**	**227**	**2,443**	**Genital**	**9.3**	**8.2, 10.5**	**10.3**	**9.2, 11.6**	**13**
**Lim 2012[171]**	**Australia**	**2006-09**	**PCR**	**488**	**7,872**	**Multisite**	**6.2**	**5.7, 6.8**	**6.2**	**5.7, 6.8**	**12**
**Goddard 2019[88]**	**Australia**	**2010-2015**	**AC2 NAAT**	**14**	**617**	**Rectal**	**2.3**	**1.4, 3.8**	**2.8**	**1.7, 4.4**	**14**

Martin-Sanchez 2020[χ90]	Australia	2011-2018	SDA (BD ProbeTec)	1413	13,780	Multisite	10.2	9.7, 10.8	4.6	4.3, 5.0	14

2. Chlamydia point prevalence data (continued)

Reference	Country	Year(s)	Diagnostic method	No. of MSM		Anatomical sample site	Uncorrected prevalence (%)	95% CI	Corrected prevalence (%)*	95% CI	Axis score
				Positive	Tested						
**Martin-Sanchez 2020[90]**	**Australia**	**2011-2018**	**SDA (BD ProbeTec)**	**1272**	**11,951**	**Multisite**	**10.7**	**10.2, 11.3**	**5.2**	**4.8, 5.6**	**14**
Martin-Sanchez 2020[90]	Australia	2011-2018	SDA (BD ProbeTec)	410	11,699	Genital	3.5	3.2, 3.9	0.0	0.0, 0.0	14
Martin-Sanchez 2020[90]	Australia	2011-2018	SDA (BD ProbeTec)	989	11,043	Rectal	9.0	8.4, 9.5	11.8	11.2, 12.4	14
Martin-Sanchez 2020[χ90]	Australia	2011-2018	SDA (BD ProbeTec)	483	13,584	Genital	3.6	3.3, 3.9	0.0	0.0, 0.0	14
Martin-Sanchez 2020[χ90]	Australia	2011-2018	SDA (BD ProbeTec)	1072	12,447	Rectal	8.6	8.1, 9.1	11.2	10.7, 11.8	14
Cornelisse 2017[172]	Australia	2011-2015	SDA	46	946	Rectal	5.0	3.7, 6.4	6.4	5.0, 8.1	15
Cornelisse 2017[172]	Australia	2011-2015	SDA	30	946	Genital	3.2	2.2, 4.5	0.0	0.0, 0.4	15
**Cornelisse 2018[92]**	**Australia**	**2015**	**NAAT**	**40**	**395**	**Rectal**	**10.1**	**7.5, 13.5**	**13.0**	**10.0, 16.7**	**14**
**Cornelisse 2018[94]**	**Australia**	**2016-2018**	**AC2 NAAT**	**123**	**1,772**	**Rectal**	**6.9**	**5.,9 8.2**	**7.4**	**6.3, 8.8**	**16**
**Saxton 2022[95]**	**New Zealand**	**2017-2019**	**SDA (BD ProbeTec)**	**34**	**150**	**Multisite**	**22.7**	**16.7, 30.0**	**19.0**	**13.5, 26.0**	**15**
**Tabesh 2022[96]**	**Australia**	**2018-2019**	**AC2 NAAT**	**498**	**3,938**	**Multisite**	**12.6**	**11.6, 13.7**	**14.3**	**13.2, 15.4**	**13**
Tabesh 2022[96]	Australia	2018-2019	AC2 NAAT	122	3,938	Genital	3.1	2.6, 3.7	2.9	2.4, 3.5	13
Tabesh 2022[96]	Australia	2018-2019	AC2 NAAT	394	3,938	Rectal	10.0	9.1, 11.0	12.8	11.8, 13.9	13
Tabesh 2022[96]	Australia	2018-2019	AC2 NAAT	87	3,938	Pharyngeal	2.2	1.8, 2.7	2.5	2.1, 3.0	13
**Oceania**											
**Badman 2018[97]**	**Papua New Guinea**	**2016**	**Xpert CT/NG**	**61**	**733**	**Rectal**	**8.3**	**6.5, 10.6**	**10.6**	**8.6, 13.1**	**15**
**Hakim 2021[98]**	**Papua New Guinea[∇]**	**2017**	**GeneXpert Ct/NG**	**79**	**400**	**Multisite**	**19.8**	**16.1, 23.9**	**23.0**	**19.2, 27.4**	**15**
**Hakim 2021[98]**	**Papua New Guinea[∇]**	**2017**	**GeneXpert Ct/NG**	**70**	**352**	**Multisite**	**19.9**	**16.1, 24.4**	**22.2**	**18.2, 26.8**	**15**
**Hakim 2021[98]**	**Papua New Guinea[∇]**	**2017**	**GeneXpert Ct/NG**	**22**	**111**	**Multisite**	**19.8**	**13.5, 28.2**	**28.3**	**20.8, 37.3**	**15**
Hakim 2021[98]	**Papua New Guinea**	2017	GeneXpert Ct/NG	42	400	Genital	10.5	7.9, 13.9	11.8	9.0, 15.3	15
Hakim 2021[98]	**Papua New Guinea**	2017	GeneXpert Ct/NG	30	400	Rectal	7.6	5.3, 10.5	9.7	7.2, 13.0	15
Hakim 2021[98]	**Papua New Guinea**	2017	GeneXpert Ct/NG	46	352	Genital	12.6	7.7, 20.1	14.6	11.3, 18.7	15
Hakim 2021[98]	**Papua New Guinea**	2017	GeneXpert Ct/NG	17	352	Rectal	4.8	3.0, 7.6	6.9	3.5, 13.3	15
Hakim 2021[98]	**Papua New Guinea**	2017	GeneXpert Ct/NG	14	111	Genital	13.1	9.9, 17.0	17.2	11.3, 25.2	15
Hakim 2021[98]	**Papua New Guinea**	2017	GeneXpert Ct/NG	4	111	Rectal	3.6	1.4, 8.9	7.3	5.1, 10.5	15
**Europe and North America**											
**Moncada 2009[3]**	**USA**	**NR**	**NAAT-AC2**	**66**	**907**	**Rectal**	**7.3**	**5.8, 9.2**	**9.3**	**7.6, 11.4**	**12**
Moncada 2009[3]	USA	NR	NAAT-AC2	60	882	Genital	6.8	5.3, 8.7	7.3	5.8, 9.2	12
**Moncada 2015[99]**	**USA**	**NR**	**NAAT-AC2**	**24**	**260**	**Multisite**	**9.2**	**6.3, 13.4**	**10.2**	**7.1, 14.5**	**12**
**Sexton 2011[100]**	**USA**	**NR**	**NAAT-AC2**	**38**	**286**	**Rectal**	**13.3**	**9.8, 17.7**	**17.2**	**13.2, 22.0**	**12**
Sexton 2011[100]	USA	NR	NAAT-AC2	5	286	Pharyngeal	1.8	0.8, 4.0	2.0	0.9, 4.4	12
**Benn 2007[101]**	**England**	**1999-2001**	**LCR**	**66**	**599**	**Multisite**	**11.1**	**8.8, 13.8**	**11.4**	**9.1, 14.2**	**11**
**Manavi 2004[173]**	**Scotland**	**1999-02**	**LCR**	**32**	**443**	**Rectal**	**7.2**	**5.2, 10.0**	**7.2**	**5.1, 10.0**	**11**
**Leuridan 2005[102]**	**Belgium**	**1999-04**	**NAAT-Cobas Amplicor**	**11**	**115**	**Genital**	**9.6**	**5.4, 16.3**	**10.7**	**6.3, 17.7**	**11**

**Bloomfield 2002[α][103]**	**USA**	**2000**	**SDA (BD ProbeTec)**	**1**	**76**	**Genital**	**1.3**	**0.2, 7.1**	**0.0**	**0.0, 4.8**	**12**

2. Chlamydia point prevalence data (continued)

Reference	Country	Year(s)	Diagnostic method	No. of MSM		Anatomical sample site	Uncorrected prevalence (%)	95% CI	Corrected prevalence (%)*	95% CI	Axis score
				Positive	Tested						
**Javanbakht 2009[106]**	**USA**	**2000-05**	**NAAT**	**127**	**4,157**	**Genital**	**3.1**	**2.6, 3.6**	**2.9**	**2.4, 3.4**	**10**
**Dilley 2003[107]**	**USA**	**2001**	**NAAT**	**65**	**2,380**	**Genital**	**2.7**	**2.2, 3.5**	**2.4**	**1.9, 3.1**	**11**
**Russell 2007[108]**	**USA**	**2002**	**LCR**	**4**	**206**	**Genital**	**2.0**	**0.8, 4.9**	**1.6**	**0.6, 4.4**	**13**
**Vall-Mayans 2007[109]**	**Spain**	**2002-03**	**PCR**	**6**	**132**	**Genital**	**4.5**	**2.1, 9.6**	**4.6**	**2.1, 9.6**	**12**
**Kent 2005[φ][110]**	**USA**	**2003**	**SDA (BD ProbeTec)**	**290**	**3,300**	**Rectal**	**8.8**	**7.9, 9.8**	**11.5**	**10.5, 12.6**	**12**
**Kent 2005[θ][110]**	**USA**	**2003**	**SDA (BD ProbeTec)**	**41**	**719**	**Rectal**	**5.7**	**4.2, 7.6**	**7.5**	**5.7, 9.6**	**12**
Kent 2005[θ][110]	USA	2003	SDA (BD ProbeTec)	292	5,305	Genital	5.5	4.9, 6.2	0.0	0.0, 0.1	12
Kent 2005[θ][110]	USA	2003	SDA (BD ProbeTec)	26	783	Genital	3.3	2.3, 4.8	0.0	0.0, 0.5	12
Kent 2005[θ][110]	USA	2003	SDA (BD ProbeTec)	61	4,658	Pharyngeal	1.3	1.0, 1.7	3.9	3.4, 4.5	12
Kent 2005[θ][110]	USA	2003	SDA (BD ProbeTec)	12	719	Pharyngeal	1.7	1.0, 2.9	5.1	3.7, 7.0	12
**Mayer 2012[111]**	**USA**	**2004-10**	**NAAT-AC2**	**12**	**898**	**Genital**	**1.3**	**0.8, 2.3**	**0.7**	**0.3, 1.5**	**13**
**Annan 2009[112]**	**England**	**2005-06**	**SDA (BD ProbeTec)**	**247**	**3,017**	**Rectal**	**8.2**	**7.3, 9.2**	**10.7**	**9.7, 11.9**	**11**
Annan 2009[112]	England	2005-06	SDA (BD ProbeTec)	41	3,076	Pharyngeal	1.3	1.0, 1.8	3.9	3.3, 4.6	11
Annan 2009[112]	England	2005-06	SDA (BD ProbeTec)	165	3,076	Genital	5.4	4.6, 6.2	1.9	1.5, 2.5	11
**Bozicevic 2008[113]**	**Croatia**	**2006**	**NAAT-AC2**	**46**	**360**	**Genital**	**12.8**	**9.7, 16.6**	**14.5**	**11.3, 18.5**	**11**
**Baker 2009[114]**	**USA**	**2006-07**	**SDA (BD ProbeTec)**	**4**	**147**	**Multisite**	**2.8**	**1.1, 6.8**	**0.0**	**0.0, 2.5**	**9**
Baker 2009[114]	USA	2006-07	SDA (BD ProbeTec)	3	147	Rectal	2.1	0.7, 5.8	2.7	1.1, 6.8	9
Baker 2009[114]	USA	2006-07	SDA (BD ProbeTec)	0	147	Genital	0.0	0.0, 2.6	0.0	0.0, 2.5	9
Baker 2009[114]	USA	2006-07	SDA (BD ProbeTec)	1	147	Pharyngeal	0.7	0.1, 3.8	2.1	0.7, 5.9	9
**Baud 2008 [174]**	**Switzerland**	**2006-07**	**PCR**	**0**	**9**	**Genital**	**0.0**	**0.0, 29.9**	**0.0**	**0.0, 29.9**	**10**
**Ota 2009[115]**	**Canada**	**2006-08**	**NAAT-AC2**	**19**	**248**	**Rectal**	**7.7**	**5.0, 11.7**	**9.8**	**6.7, 14.2**	**13**
Ota 2009[115]	Canada	2006-08	NAAT-AC2	5	248	Pharyngeal	2.0	9.0, 4.6	2.3	1.0, 5.0	13
Ota 2009[115]	Canada	2006-08	NAAT-AC2	12	248	Genital	4.8	2.8, 8.3	4.9	2.9, 8.4	13
**Smelov 2017[175]**	**Russian Fed**	**2006-09**	**PCR**	**4**	**14**	**Multisite**	**28.6**	**11.7, 54.7**	**32.1**	**13.9, 57.9**	**14**
**Bernstein 2009[116]**	**USA**	**2007**	**GenProbe APTIMA**	**19**	**397**	**Genital**	**4.8**	**2.8, 8.3**	**4.9**	**3.2, 7.5**	**12**
**Klausner 2009[117]**	**USA**	**2007**	**NAAT**	**465**	**4,806**	**Rectal**	**9.7**	**8.9, 10.5**	**12.5**	**11.5, 13.4**	**12**
**Klausner 2009[117]**	**USA**	**2007**	**Culture**	**3**	**452**	**Rectal**	**0.7**	**0.2, 1.9**	**0.0**	**0.0, 0.8**	**12**
Klausner 2009[117]	USA	2007	Culture	0	13	Pharyngeal	0.0	0.0, 22.8	0.0	0.0, 22.8	12
Klausner 2009[117]	USA	2007	NAAT	54	3397	Pharyngeal	1.6	1.2, 2.1	1.8	1.4, 2.3	12
**Mimiaga 2008[118]**	**USA**	**2007**	**SDA (BD ProbeTec)**	**7**	**107**	**Rectal**	**6.1**	**3.2, 12.9**	**8.0**	**4.2, 14.7**	**13**
Mimiaga 2008[118]	USA	2007	SDA (BD ProbeTec)	3	111	Genital	2.6	0.9, 7.7	0.0	0.0, 3.3	13
**Fisher 2015[119]**	**England**	**2008**	**NAAT-AC2**	**18**	**202**	**Multisite**	**9.0**	**5.7, 13.6**	**10.0**	**6.6, 14.9**	**16**
Fisher 2015[119]	England	2008	NAAT-AC2	2	202	Pharyngeal	1.0	0.3, 3.5	1.1	0.3, 3.8	16
Fisher 2015[119]	England	2008	NAAT-AC2	3	202	Genital	1.5	0.5, 4.3	1.0	0.3, 3.5	16

Fisher 2015[119]	England	2008	NAAT-AC2	13	202	Rectal	6.4	3.8, 10.7	8.1	5.1, 12.7	16

2. Chlamydia point prevalence data (continued)

Reference	Country	Year(s)	Diagnostic method	No. of MSM		Anatomical sample site	Uncorrected prevalence (%)	95% CI	Corrected prevalence (%)*	95% CI	Axis score
				Positive	Tested						
**Soni 2009[120]**	**England**	**2008**	**SDA (BD ProbeTec)**	**39**	**850**	**Multisite**	**7.8**	**3.4, 6.2**	**1.8**	**1.1, 3.0**	**13**
Soni 2009[120]	England	2008	SDA (BD ProbeTec)	23	412	Rectal	5.6	3.8, 8.2	7.3	5.2, 10.2	13
Soni 2009[120]	England	2008	SDA (BD ProbeTec)	16	438	Genital	3.7	2.3, 5.9	0.0	0.0, 0.9	13
**Dodge 2012[χ176]**	**USA**	**2009**	**NAAT-AC2**	**6**	**58**	**Rectal**	**10.3**	**4.8, 20.8**	**13.2**	**6.8, 24.3**	**7**
**Freeman 2011[121]**	**USA**	**2009**	**NAAT-AC2**	**6**	**473**	**Pharyngeal**	**1.3**	**0.6, 2.7**	**1.5**	**0.7, 3.0**	**11**
**Cuypers 2011[122]**	**Netherlands**	**2009-10**	**NAAT**	**18**	**99**	**NR**	**18.2**	**11.8, 26.9**	**20.1**	**13.4, 29.0**	**9**
**Dudareva-Vizule 2014[123]**	**Germany**	**2009-10**	**NAAT-AC2**	**164**	**2,050**	**Rectal**	**8.0**	**6.9, 9.3**	**10.2**	**9.0, 11.6**	**15**
Dudareva-Vizule 2014[123]	Germany	2009-10	NAAT-AC2	32	2,197	Pharyngeal	1.5	1.0, 2.1	1.7	1.2, 2.3	15
Dudareva-Vizule 2014[123]	Germany	2009-10	NAAT-AC2	23	685	Genital	3.4	2.3, 5.0	3.0	2.0, 4.6	15
**Reinton 2013[125]**	**Norway**	**2009-11**	**NAAT-Roche Amplicor**	**228**	**2,284**	**Multisite**	**10.0**	**8.8, 11.3**	**11.2**	**9.9, 12.5**	**13**
Reinton 2013[125]	Norway	2009-11	NAAT-Roche Amplicor	64	2,284	Genital	2.8	2.2, 3.6	3.0	2.4, 3.8	13
Reinton 2013[125]	Norway	2009-11	NAAT-Roche Amplicor	182	2,284	Rectal	8.0	6.9, 9.1	10.2	9.0, 11.5	13
**Mayer 2014[124]**	**USA**	**2010**	**NAAT-AC2**	**93**	**1,553**	**Rectal**	**6.0**	**4.9, 7.3**	**7.6**	**6.4, 9.0**	**13**
Mayer 2014[124]	USA	2010	NAAT-AC2	31	1,553	Genital	2.0	1.4, 2.8	1.6	1.1, 2.3	13
**Park 2012[126]**	**USA**	**2010**	**NAAT-AC2**	**210**	**12,454**	**Pharyngeal**	**1.7**	**1.5, 1.9**	**1.9**	**1.7, 2.2**	**13**
**Perkins 2011[θ127]**	**USA**	**2010**	**NAAT**	**14**	**183**	**Rectal**	**7.7**	**4.6, 12.4**	**9.8**	**6.3, 14.9**	**9**
**Perkins 2011[φ127]**	**USA**	**2010**	**NAAT**	**124**	**1,138**	**Rectal**	**10.9**	**9.2, 12.8**	**14.0**	**12.1, 16.2**	**9**
Perkins 2011[θ127]	USA	2010	NAAT	5	184	Genital	2.7	1.2, 6.2	2.4	1.0, 5.8	9
Perkins 2011[θ127]	USA	2010	NAAT	2	189	Pharyngeal	1.1	0.3, 3.8	1.2	0.4, 4.0	9
Perkins 2011[θ127]	USA	2010	NAAT	139	1753	Genital	7.9	6.8, 9.3	8.7	7.5, 10.1	9
**Bozicevic 2012[128]**	**Croatia**	**2010-11**	**Abbott Realtime PCR**	**33**	**387**	**Pharyngeal**	**8.5**	**6.1, 11.7**	**9.4**	**6.8, 12.7**	**14**
**Van Liere 2013[129]**	**Netherlands**	**2010-11**	**SDA (BD ProbeTec)**	**71**	**674**	**Multisite**	**10.5**	**8.4, 13.1**	**4.9**	**3.6, 6.9**	**14**
**Van Liere 2013[χ129]**	**Netherlands**	**2010-11**	**SDA (BD ProbeTec)**	**14**	**252**	**Multisite**	**5.7**	**3.3, 9.1**	**0.0**	**0.0, 1.5**	**14**
**Remis 2016[130]**	**Canada**	**2010-12**	**SDA (BD ProbeTec)**	**0**	**148**	**Genital**	**0.0**	**0.0, 2.5**	**0.0**	**0.0, 2.5**	**11**
**Van Liere 2015[131]**	**Netherlands**	**2011-12**	**NAAT-cobas amplicor**	**693**	**7,094**	**Rectal**	**9.8**	**9.1, 10.5**	**12.6**	**11.8, 13.4**	**13**
**Van Rooijen 2015[177]**	**Netherlands**	**2011-12**	**NAAT-AC2**	**909**	**10,140**	**Rectal**	**9.0**	**8.4, 9.5**	**11.5**	**10.9, 12.2**	**13**
Van Rooijen 2015[177]	Netherlands	2011-12	NAAT-AC2	570	13,065	Genital	4.4	4.0, 4.7	4.4	4.1, 4.8	13
Van Rooijen 2015[177]	Netherlands	2011-12	NAAT-AC2	148	13,111	Pharyngeal	1.1	1.0, 1.3	1.3	1.1, 1.5	13
**Nelson 2019[136]**	**Canada**	**2011-2013**	**SDA (BD ProbeTec)**	**3**	**86**	**Multisite**	**3.5**	**1.2, 9.8**	**0.0**	**0.0, 4.3**	**13**
**Bamberger 2019[χ135]**	**USA**	**2012-2014**	**AC2 NAAT**	**37**	**215**	**Multisite**	**17.2**	**12.8, 22.8**	**19.8**	**15.0, 25.6**	**9**
**Bamberger 2019[135]**	**USA**	**2012-2014**	**AC2 NAAT**	**165**	**1,002**	**Multisite**	**16.5**	**14.3, 18.9**	**19.0**	**16.7, 21.5**	**9**
Bamberger 2019[135]	USA	2012-2014	AC2 NAAT	51	969	Genital	5.3	4.0, 6.9	5.5	4.2, 7.1	9
Bamberger 2019[135]	USA	2012-2014	AC2 NAAT	95	632	Rectal	15.0	12.5, 18.0	19.4	16.5, 22.7	9
Bamberger 2019[135]	USA	2012-2014	AC2 NAAT	24	967	Pharyngeal	2.5	1.7, 3.7	2.8	1.9, 4.1	9

2. Chlamydia point prevalence data (continued)

Reference	Country	Year(s)	Diagnostic method	No. of MSM		Anatomical sample site	Uncorrected prevalence (%)	95% CI	Corrected prevalence (%)*	95% CI	Axis score
				Positive	Tested						
Bamberger 2019[χ135]	USA	2012-2014	AC2 NAAT	21	209	Genital	10.0	6.7, 14.9	11.2	7.6, 16.22	9
Bamberger 2019[χ135]	USA	2012-2014	AC2 NAAT	15	89	Rectal	16.9	10.5, 26.0	21.8	14.5, 31.5	9
Bamberger 2019[χ135]	USA	2012-2014	AC2 NAAT	6	211	Pharyngeal	2.8	1.3, 6.1	3.2	1.6, 6.6	9
**Gratrix 2014[133]**	**Canada**	**2012**	**NAAT-AC2**	**158**	**972**	**Multisite**	**16.3**	**14.1, 18.7**	**18.7**	**16.4, 21.3**	**11**
Gratrix 2014[133]	Canada	2012	NAAT-AC2	42	972	Genital	4.3	3.2, 5.8	4.3	3.2, 5.8	11
Gratrix 2014[133]	Canada	2012	NAAT-AC2	137	972	Rectal	14.1	12.1, 16.4	18.2	15.9, 20.8	11
**Dolling 2016[137]**	**England**	**2012-13**	**NAAT**	**10**	**248**	**Rectal**	**4.0**	**2.2, 7.3**	**5.0**	**2.9, 8.4**	**12**
Dolling 2016[137]	England	2012-13	NAAT	3	244	Pharyngeal	1.0	0.4, 3.6	1.1	0.4, 3.4	12
Dolling 2016[137]	England	2012-13	NAAT	3	255	Genital	1.0	0.4, 3.4	0.2	0.0, 1.9	12
**Lallemand 2016[178]**	**Germany**	**2012-13**	**NAAT-AC2**	**19**	**549**	**Genital**	**3.5**	**2.2, 5.3**	**3.4**	**2.1, 5.2**	**14**
**Lampkin 2016[179]**	**USA**	**2012-14**	**NAAT**	**2**	**14**	**Rectal**	**14.3**	**4.0, 39.9**	**18.5**	**6.0, 44.5**	**9**
Lampkin 2016[179]	USA	2012-14	NAAT	1	14	Genital	7.1	1.3, 31.5	7.3	1.3, 31.6	9
Lampkin 2016[179]	USA	2012-14	NAAT	1	14	Pharyngeal	7.1	1.3, 31.5	8.1	1.6, 32.6	9
**Closson 2018[138]**	**USA**	**2012-15**	**NAAT**	**95**	**509**	**Rectal**	**18.7**	**15.5, 22.3**	**24.2**	**20.7, 28.2**	**11**
Closson 2018[138]	USA	2012-15	NAAT	28	519	Pharyngeal	5.4	3.8, 7.7	6.1	4.4, 8.5	11
Closson 2018[138]	USA	2012-15	NAAT	35	542	Genital	6.5	4.7, 8.9	7.0	5.1, 9.4	11
**Liu 2016[139]**	**USA**	**2012-15**	**NAAT-AC2**	**58**	**555**	**Rectal**	**10.5**	**8.2, 13.3**	**13.5**	**10.9, 16.6**	**13**
Liu 2016[139]	USA	2012-15	NAAT-AC2	18	557	Pharyngeal	2.7	2.1, 5.1	3.1	1.9, 4.9	13
Liu 2016[139]	USA	2012-15	NAAT-AC2	21	557	Genital	3.8	2.5, 5.7	3.7	2.4, 5.6	13
**Mena 2018[140]**	**USA**	**2012-15**	**NAAT-AC2**	**66**	**485**	**Rectal**	**13.6**	**10.8, 17.0**	**17.6**	**14.4, 21.2**	**13**
Mena 2018[140]	USA	2012-15	NAAT-AC2	16	485	Genital	3.3	2.0, 5.3	3.1	1.9, 5.1	13
Mena 2018[140]	USA	2012-15	NAAT-AC2	12	485	Pharyngeal	2.5	1.4, 4.3	2.8	1.7, 4.7	13
**Ruutel 2015[141]**	**Estonia**	**2013**	**PCR**	**1**	**65**	**Genital**	**2.0**	**0.3, 8.2**	**1.6**	**0.3, 8.2**	**14**
**Tang, 2020[142]**	**USA**	**2012-2014**	**AC2 NAAT**	**75**	**557**	**Multisite**	**13.5**	**10.9, 16.6**	**15.4**	**12.6, 18.6**	**11**
**Calas 2021[143]**	**France**	**2014-2015**	**FTD Urethritis Basic Kit**	**2**	**27**	**Rectal**	**7.4**	**2.1, 23.4**	**9.4**	**3.0, 26.0**	**12**
Calas 2021[143]	France	2014-2015	FTD Urethritis Basic Kit	2	53	Genital	3.8	1.0, 12.8	3.7	1.0, 12.6	12
Calas 2021[143]	France	2014-2015	FTD Urethritis Basic Kit	2	45	Pharyngeal	4.4	1.2, 14.8	5.0	1.5, 15.7	12
**Mustanski 2018[144]**	**America**	**2013-15**	**NAAT-AC2**	**77**	**891**	**Rectal**	**8.6**	**7.0, 10.7**	**11.0**	**9.1, 13.2**	**18**
Mustanski 2018[144]	America	2013-15	NAAT-AC2	22	893	Genital	2.5	1.6, 3.7	2.2	1.4, 3.3	18
**Grov 2016[148]**	**USA**	**2014**	**Abbott Realtime Assay**	**47**	**1,071**	**Rectal**	**4.4**	**3.3, 5.8**	**5.5**	**4.3, 7.0**	**12**
Grov 2016[148]	USA	2014	Abbott Realtime Assay	15	1,070	Genital	1.4	0.9, 2.3	0.8	0.4, 1.6	12
**Van der Veer 2016[132]**	**Netherlands**	**2014**	**NAAT-AC2**	**31**	**678**	**Genital**	**4.6**	**3.2, 6.4**	**4.7**	**3.3, 6.5**	**13**
**Achterbeg 2019[146]**	**Netherlands**	**2017**	**NAAT**	**435**	**4,465**	**Multisite**	**9.8**	**8.9, 10.7**	**10.9**	**10.0, 11.8**	**12**
Achterbeg 2019[146]	Netherlands	2017	NAAT	144	4,447	Genital	3.2	2.8, 3.8	3.0	2.6, 3.6	12

Achterbeg 2019[146]	Netherlands	2017	NAAT	320	4,399	Rectal	7.3	6.5, 8.1	9.3	8.4, 10.2	12

2. Chlamydia point prevalence data (continued)

Reference	Country	Year(s)	Diagnostic method	No. of MSM		Anatomical sample site	Uncorrected prevalence (%)	95% CI	Corrected prevalence (%)*	95% CI	Axis score
				Positive	Tested						
Achterbeg 2019[146]	Netherlands	2017	NAAT	61	4,427	Pharyngeal	1.4	1.1, 1.8	1.6	1.2, 2.0	12
**Achterbeg 2019[145]**	**Netherlands**	**2014-2016**	**AC2 NAAT**	**81**	**1,130**	**Multisite**	**7.2**	**5.8, 8.8**	**7.8**	**6.4, 9.5**	**12**
**Ceccarani 2019[147]**	**Italy**	**2015**	**Versant CT/NG DNA assay**	**55**	**125**	**Rectal**	**44.0**	**35.6, 52.8**	**51.0**	**42.3, 59.6**	**11**
**Chan 2018[149]**	**USA**	**2014-17**	**NAAT-AC2**	**49**	**415**	**Multisite**	**11.8**	**9.1, 15.3**	**13.3**	**10.4, 16.9**	**12**
**Wilson 2021[151]**	**UK**	**2015-2016**	**NAAT-AC2**	**39**	**509**	**Multisite**	**7.7**	**5.7, 10.3**	**8.4**	**6.3, 11.1**	**12**
Wilson 2021[151]	UK	2015-2016	NAAT-AC2	11	509	Genital	2.2	1.2, 3.8	1.8	1.0, 3.4	12
Wilson 2021[151]	UK	2015-2016	NAAT-AC2	31	509	Rectal	6.1	4.3, 8.5	7.7	5.7, 10.4	12
Wilson 2021[151]	UK	2015-2016	NAAT-AC2	5	509	Pharyngeal	1.0	0.4, 2.3	1.1	0.5, 2.5	12
**Druckler 2018[152]**	**Netherlands**	**2016**	**NAAT-AC2**	**387**	**4,925**	**Rectal**	**7.9**	**7.1, 8.6**	**10.1**	**9.3, 11.0**	**10**
Druckler 2018[152]	Netherlands	2016	NAAT-AC2	176	4,925	Genital	3.6	3.1, 4.1	3.5	3.0, 4.0	10
Druckler 2018[152]	Netherlands	2016	NAAT-AC2	59	4,925	Pharyngeal	1.2	0.9, 1.5	1.4	1.1, 1.7	10
**Rondaeu 2019[180]**	**France**	**2016**	**NAAT-AC2**	**13**	**111**	**Rectal**	**17.1**	**7.0, 19.0**	**22.1**	**15.4, 30.7**	**11**
**Salow 2017[153]**	**USA**	**2016**	**Gene Xpert NAAT**	**18**	**145**	**Rectal**	**12.4**	**8.0, 18.8**	**16.0**	**10.9, 22.8**	**11**
Salow 2017[153]	USA	2016	Gene Xpert NAAT	2	148	Pharyngeal	1.4	0.4, 4.8	1.6	0.5, 5.2	11
**Abara 2020[154]**	**USA**	**2015-2019**	**NAAT**	**18639**	**139,718**	**Multisite**	**13.3**	**13.2, 13.5**	**14.4**	**14.2, 14.6**	**12**
Abara 2020[154]	USA	2015-2019	NAAT	6412	126,747	Genital	5.2	4.9, 5.2	5.1	5.0, 5.2	12
Abara 2020[154]	USA	2015-2019	NAAT	12631	101,338	Rectal	12.6	12.3, 12.7	16.3	16.0, 16.5	12
Abara 2020[154]	USA	2015-2019	NAAT	1721	93,172	Pharyngeal	1.8	1.8, 1.9	2.0	2.0, 2.1	12
**Barbee 2021[155]**	**USA**	**2016-2018**	**AC2 NAAT**	**14**	**140**	**Rectal**	**10.0**	**6.1, 16.1**	**11.5**	**7.2, 17.9**	**14**
Barbee 2021[155]	USA	2016-2018	AC2 NAAT	2	140	Genital	1.4	0.4, 5.1	0.4	0.0, 3.3	14
Barbee 2021[155]	USA	2016-2018	AC2 NAAT	2	140	Pharyngeal	1.4	0.4, 5.1	1.1	0.3, 4.6	14
**Chapin-Bardales 2020[156]**	**USA**	**2017**	**AC2 NAAT**	**22**	**1,627**	**Pharyngeal**	**1.4**	**0.9, 2.0**	**1.6**	**1.1, 2.3**	**15**
Chapin-Bardales 2020[156]	USA	2017	AC2 NAAT	11	1,627	Pharyngeal	0.7	0.4, 1.2	0.6	0.3, 1.1	15
**Foschi 2018[157]**	**Italy**	**2017**	**NAAT**	**42**	**165**	**Multisite**	**25.4**	**19.4, 32.6**	**29.7**	**23.2, 37.0**	**12**
**Harvey-Lavoie 2020[159]**	**Canada**	**2016-2017**	**NAAT (Cobas Amplicor)**	**44**	**1,177**	**Multisite**	**3.7**	**2.8, 5.0**	**2.5**	**1.8, 3.6**	**15**
Harvey-Lavoie 2020[159]	Canada	2016-2017	NAAT (Cobas Amplicor)	9	1,171	Genital	0.4	0.2, 1.1	0.0	0.0, 0.3	15
Harvey-Lavoie 2020[159]	Canada	2016-2017	NAAT (Cobas Amplicor)	33	1,156	Rectal	2.4	2.0, 4.0	2.9	2.1, 4.0	15
Harvey-Lavoie 2020[159]	Canada	2016-2017	NAAT (Cobas Amplicor)	6	1,175	Pharyngeal	0.4	0.2, 1.1	0.5	0.2, 1.0	15
**Johnson-Jones 2019[158]**	**USA**	**2017**	**NAAT-AC2**	**148**	**2,024**	**Rectal**	**7.3**	**6.3, 8.5**	**9.3**	**8.1, 10.6**	**13**
Johnson-Jones 2019	USA	2017	NAAT-AC2	29	2,072	Pharyngeal	1.4	1.0, 2.0	1.6	1.1, 2.2	13
**Fernandez-Huerta 2021[160]**	**Spain**	**2017-2018**	**Gene Xpert CT/NG**	**48**	**489**	**Multisite**	**9.8**	**7.5, 12.8**	**10.9**	**8.5, 14.0**	**11**
**Jansen 2020[161]**	**Germany**	**2018**	**AC2 NAAT**	**227**	**2,203**	**Multisite**	**10.3**	**9.1, 11.6**	**10.0**	**8.8, 11.3**	**15**
Jansen 2020[161]	Germany	2018	AC2 NAAT	45	2,203	Genital	2.0	1.5, 2.7	1.6	1.1, 2.2	15
Jansen 2020[161]	Germany	2018	AC2 NAAT	178	2,203	Rectal	8.1	7.0, 9.3	9.8	8.7, 11.1	15

Jansen 2020[161]	Germany	2018	AC2 NAAT	26	2,203	Pharyngeal	1.1	0.8, 1.7	1.3	0.9, 1.8	15
**Rahib 2022[162]**	**France**	**2018**	**Cobas Amplicor**	**180**	**1,930**	**Multisite**	**9.3**	**8.1, 10.7**	**10.3**	**9.0, 11.8**	**18**

2. Chlamydia point prevalence data (continued)

Reference	Country	Year(s)	Diagnostic method	No. of MSM		Anatomical sample site	Uncorrected prevalence (%)	95% CI	Corrected prevalence (%)*	95% CI	Axis score
				Positive	Tested						
Rahib 2022[162]	France	2018	Cobas Amplicor	36	1,930	Genital	1.9	1.4, 2.6	1.4	1.0, 2.0	18
Rahib 2022[162]	France	2018	Cobas Amplicor	120	1,930	Rectal	7.3	6.2, 8.5	9.2	8.0, 10.6	18
Rahib, 2022[162]	France	2018	Cobas Amplicor	34	1,930	Pharyngeal	1.8	1.3, 2.5	2.0	1.5, 2.7	18
**Assaf, 2022[163]**	**USA**	**2018-2020**	**AC2 NAAT**	**1,585**	**4,969**	**Multisite**	**31.9**	**30.6, 33.2**	**37.5**	**36.1, 38.8**	**13**
**Assaf 2022[χ][163]**	**USA**	**2018-2020**	**AC2 NAAT**	**77**	**226**	**Multisite**	**34.1**	**28.2, 40.5**	**40.1**	**33.9, 46.6**	**13**
Assaf 2022[163]	USA	2018-2020	AC2 NAAT	413	16,742	Genital	2.5	2.2, 2.7	2.1	1.9, 2.4	13
Assaf 2022[163]	USA	2018-2020	AC2 NAAT	1,316	16,424	Rectal	8.0	7.6, 8.4	8.6	6.9, 10.8	13
Assaf 2022[163]	USA	2018-2020	AC2 NAAT	26	3,776	Pharyngeal	0.6	0.1, 3.3	0.8	0.5, 1.1	13
Assaf 2022[χ][163]	USA	2018-2020	AC2 NAAT	31	822	Genital	3.8	2.7, 5.3	3.7	2.6, 5.2	13
Assaf 2022[χ][163]	USA	2018-2020	AC2 NAAT	54	798	Rectal	6.8	5.2, 8.7	10.2	9.8, 10.7	13
Assaf 2022[χ][163]	USA	2018-2020	AC2 NAAT	1	167	Pharyngeal	0.6	0.1, 3.3	0.7	0.1, 3.4	13
**Samarasekara 2021[181]**	**UK**	**2019**	**NAAT**	**75**	**6,613**	**Pharyngeal**	**1.1**	**0.9, 1.4**	**1.5**	**1.2, 1.8**	**9**
**Tyulnev, 2020[164]**	**Russian Fed**	**2019-2020**	**PCR-RT**	**63**	**518**	**Rectal**	**12.2**	**9.6, 15.3**	**15.5**	**12.6, 18.8**	**5**
Tyulnev 2020[164]	Russian Fed	2019-2020	PCR-RT	22	518	Genital	4.2	2.8, 6.3	4.2	2.8, 6.3	5
Tyulnev 2020[164]	Russian Fed	2019-2020	PCR-RT	22	518	Pharyngeal	4.2	2.8, 6.3	4.8	3.2, 7.0	5

The bold rows are the included data points in the meta-analysis.
The non-bold rows are additional data points from the same paper from different anatomical sites not included in the meta-analysis due to the paper providing a multisite value or another anatomical site prevalence which was higher.
Studies where there are multiple data points included from the same paper are presented as multiple bold rows, due to different study years (documented in the year(s) column), different locations (city documented in the footnote below) and different populations within the same study (documented using notation which is defined in the footnote below).
* Prevalence corrected for sensitivity and specificity of diagnostic assay used
** Burkina Faso, Togo, Mali, Cote D'Ivoire
UK: United Kingdom of Great Britain and Northern Ireland
USA: United States of America
UR Tanzania: United Republic of Tanzania
[λ]: Male sex worker cohort [δ]: Transgender female sex worker cohort [χ]: Men who have sex with men and women cohort [ϕ]: Transgender female cohort [α]: 95% MSM cohort (5% other).
[φ]: Clinic setting [θ]: Community setting
[∇]: Studies with multiple data points due to different population locations: Tanga, Dar Es Salaam, Francistown, Gabarone, Abuja, Lagos, Lahore, Karachi, Karnataka, Andhra Pradesh, Maharashtra, Tamil Nadu, Rawalpindi, Abbottabad, Hanoi, Ho Chi Minh, Bandung, Jakarta, Surabaya, San Miguel, San Salvador, Port Moresby, Lae and Mount Hagen.
NR: not reported. For these studies we calculated the average number of years from study period to publication amongst all included studies which was 4 years and categorised the studies where no study period was reported within the subgroup of their publication date – 4 years.
For data points from studies where the study period crossed the two time-categories (2000-2011 and 2012-2022), the midpoint of the study was used to classify the data point.
[Δ]: Guatemala, Panama, Honduras, Nicaragua, El Salvador
Corrected prevalence (%): prevalence corrected for the sensitivity and specificity of the specific biological assay

3. Trichomoniasis point prevalence data among men who have sex with men by Sustainable Development Goal region

Reference	Country	Year(s)	No. of MSM		Anatomical sample site	Uncorrected prevalence (%)	95% CI	Diagnostic method	Corrected prevalence (%)*	95% CI	Axis score
			Positive	Tested							
**Sub-Saharan Africa**											
**Vuylsteke 2012[λ,11]**	**Cote d'Ivoire**	**2007-08**	**2**	**94**	**Multisite**	**2.1**	**0.7, 7.4**	**PCR**	**0.0**	**0.0, 3.9**	**12**
Vuylsteke 2012[λ]	Cote d'Ivoire	2007-08	2	94	Rectal	2.1	0.7, 7.4	PCR	0.0	0.0, 3.9	12
Vuylsteke 2012[λ]	Cote d'Ivoire	2007-08	0	94	Genital	0.0	0.0, 3.9	PCR	0.0	0.0, 3.9	12
**Ferre 2019[25]**	**Togo**	**2017**	**0**	**207**	**Rectal**	**0.0**	**0.0, 1.8**	**PCR**	**0.0**	**0.0, 1.8**	**12**
**Latin America and the Caribbean**											
**Figueroa 2013[65]**	**Jamaica**	**2007-08**	**0**	**100**	**Genital**	**0.0**	**0.0, 3.7**	**TV-ASR**	**0.0**	**0.0, 3.7**	**10**
**Creswell 2012[66]**	**El Salvador[∇]**	**2008**	**1**	**188**	**Genital**	**0.5**	**0.1, 3.0**	**PCR**	**0.0**	**0.0, 2.0**	**15**
**Creswell 2012[66]**	**El Salvador[∇]**	**2008**	**6**	**460**	**Genital**	**1.3**	**0.6, 2.6**	**PCR**	**0.0**	**0.0, 0.8**	**15**
**Europe and North America**											
**Ruutel 2015[141]**	**Estonia**	**2013**	**0**	**65**	**Genital**	**0.0**	**0.0, 5.6**	**PCR**	**0.0**	**0.0, 5.6**	**14**
**Van der Veer C 2016[132]**	**Netherlands**	**2014**	**0**	**678**	**Genital**	**0.0**	**0.0, 0.6**	**TV Aptima**	**0.0**	**0.0, 0.6**	**13**
**Jansen 2020[161]**	**Germany**	**2018**	**2**	**2,203**	**Multisite**	**0.0**	**0.0, 0.3**	**TV Aptima**	**0.0**	**0.0, 0.2**	**15**
Jansen 2020[161]	Germany	2018	0	2,203	Genital	0.0	0.0, 0.2	TV Aptima	0.0	0.0, 0.2	15
Jansen 2020[161]	Germany	2018	2	2,202	Rectal	0.0	0.0, 0.3	TV Aptima	0.0	0.0, 0.2	15
Jansen 2020[161]	Germany	2018	0	2,203	Pharyngeal	0.0	0.0, 0.2	TV Aptima	0.0	0.0, 0.2	15
**Tyulnev 2020[164]**	**Russian Fed**	**2019-2020**	**1**	**518**	**Genital**	**0.0**	**0.0, 1.1**	**Amplipram NCMT (PCR-RT)**	**0.0**	**0.0, 0.7**	**5**
Tyulnev 2020[164]	Russian Fed	2019-2020	0	518	Rectal	0.0	0.0, 0.7	Amplipram NCMT (PCR-RT)	0.0	0.0, 0.7	5
Tyulnev 2020[164]	Russian Fed	2019-2020	0	518	Pharyngeal	0.0	0.0, 0.7	Amplipram NCMT (PCR-RT)	0.0	0.0, 0.7	5

The bold rows are the included data points in the meta-analysis.
The non-bold rows are additional data points from the same paper from different anatomical sites not included in the meta-analysis due to the paper providing a multisite value or another anatomical site prevalence which was higher.
Studies where there are multiple data points included from the same paper are presented as multiple bold rows, due to different study years (documented in the year(s) column), different locations (city documented in the footnote below) and different populations within the same study (documented using notation which is defined in the footnote below).
* Prevalence corrected for sensitivity and specificity of diagnostic assay used
[λ]: Male sex worker cohort
[∇]: Studies with multiple data points due to different population locations: San Miguel, San Salvador
Corrected prevalence (%): prevalence corrected for the sensitivity and specificity of the specific biological assay

Supplemental File 3

1. Number of data points included in the meta-analysis by study year and SDG region

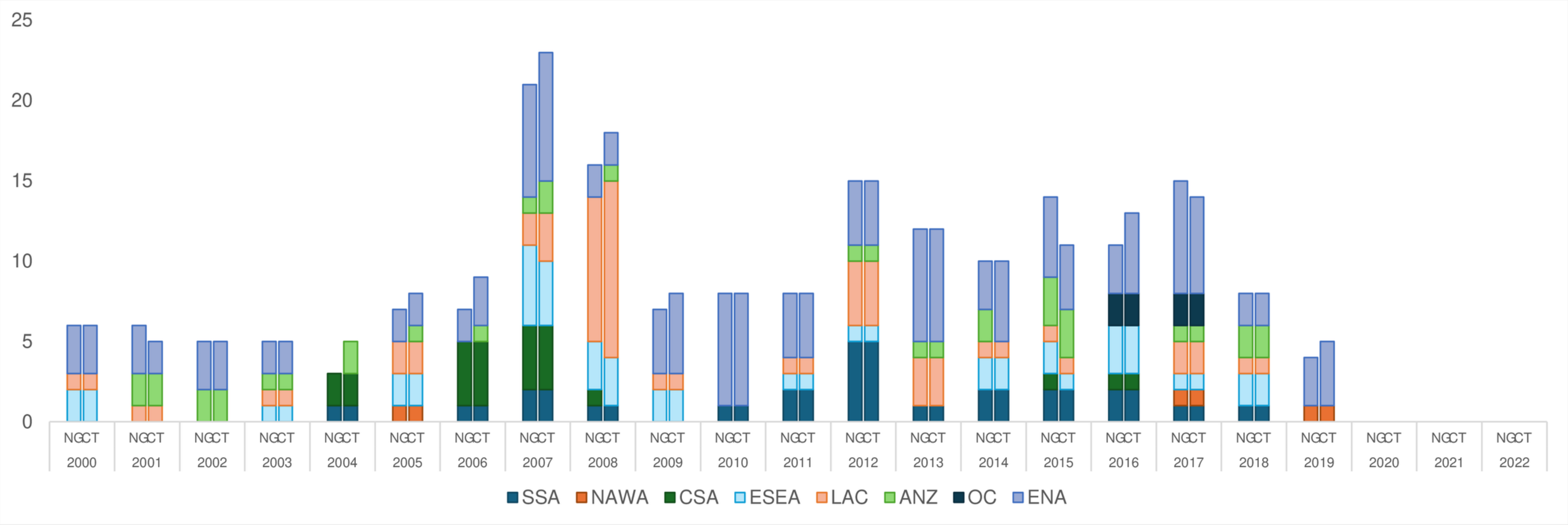

2. Summary of the appraisal tool for cross-sectional studies (AXIS) score of the data points included in the meta-analysis by Sustainable Development Goal region.

**SDG region**	**Number of data points**	
	High risk of bias (AXIS score 0-10)	Low risk of bias (AXIS score 11-20)

**Gonorrhoea**		
Sub-Saharan Africa	3	19
Northern Africa and Western Asia	2	1
Central and Southern Asia	1	12
Eastern and South-Eastern Asia	4	23
Latin America and the Caribbean	5	25
Australia and New Zealand	0	16
Oceania	0	4
Europe and North America	11	62
**Total**	26	162
	**Chlamydia**	
Sub-Saharan Africa	3	19
Northern Africa and Western Asia	2	1
Central and Southern Asia	0	11
Eastern and South-Eastern Asia	4	21
Latin America and the Caribbean	5	22
Australia and New Zealand	0	20
Oceania	0	4
Europe and North America	13	65
**Total**	27	163

3. Funnel plots

3. a) Funnel plots to assess publication bias within included gonorrheoa studies

i) Random-effects using all studies

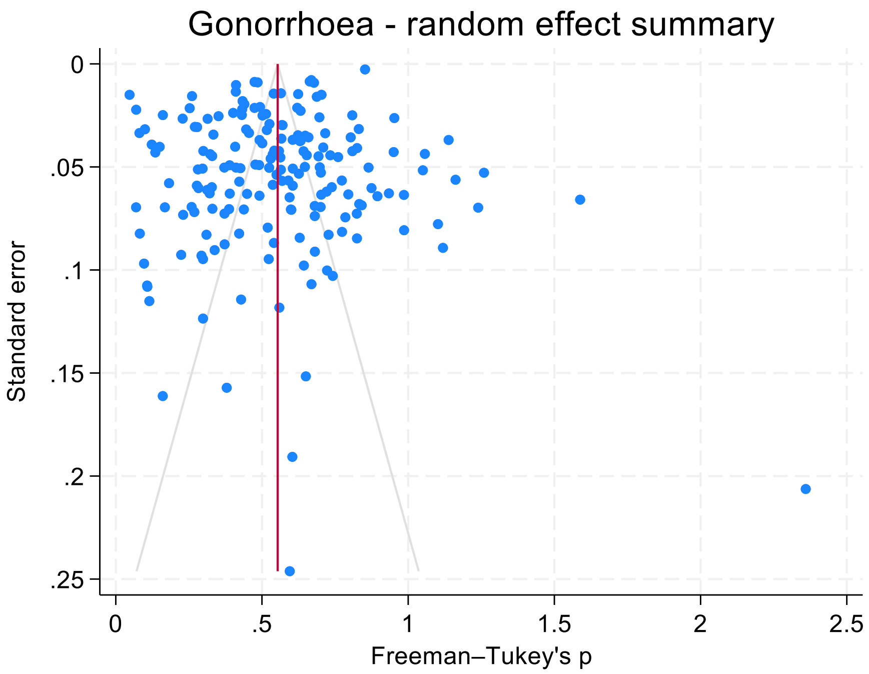

(ii) Random-effects using studies >100 tested

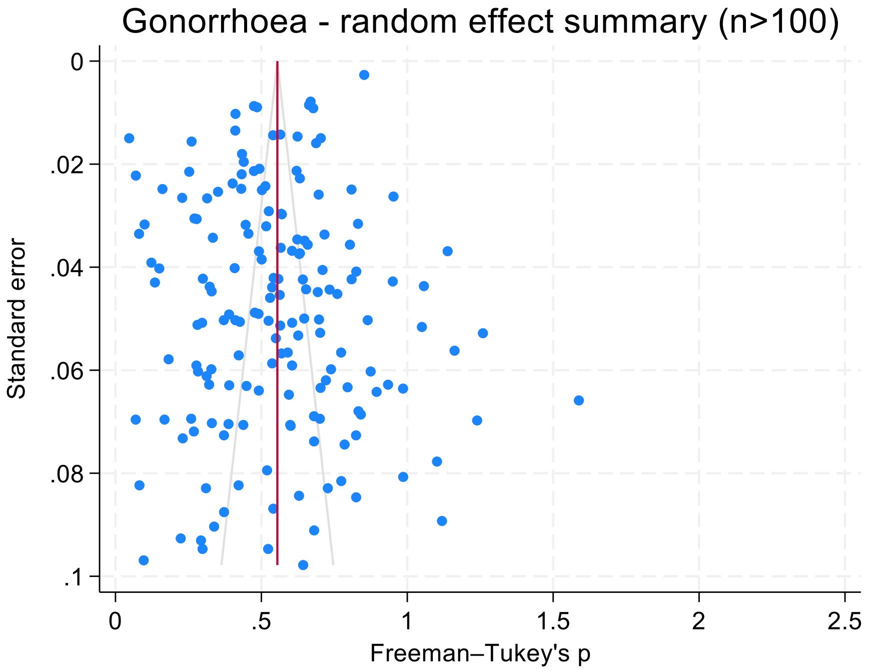

(iii) Fixed-effects using all studies

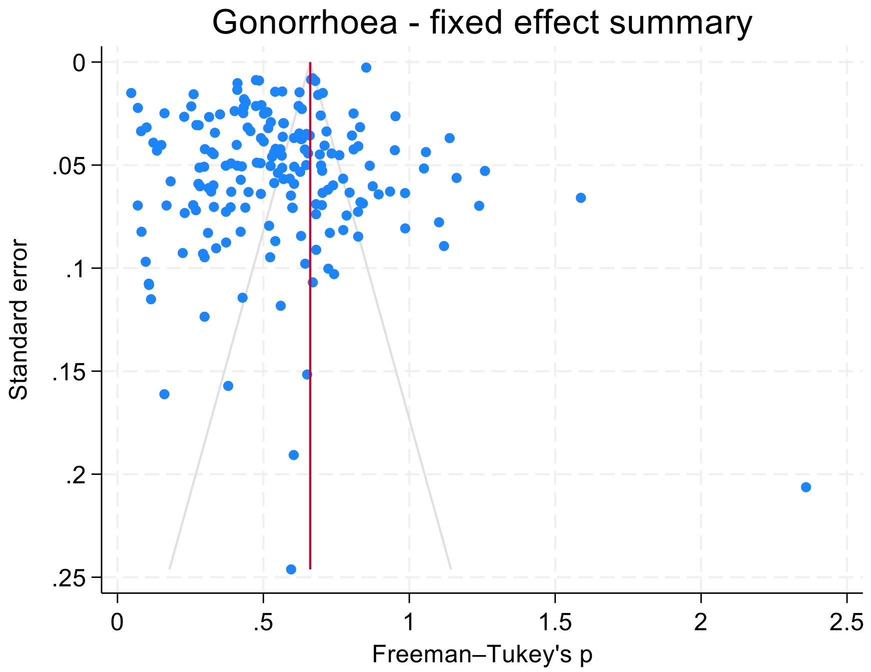

(iv) Fixed-effects using studies >100 tested

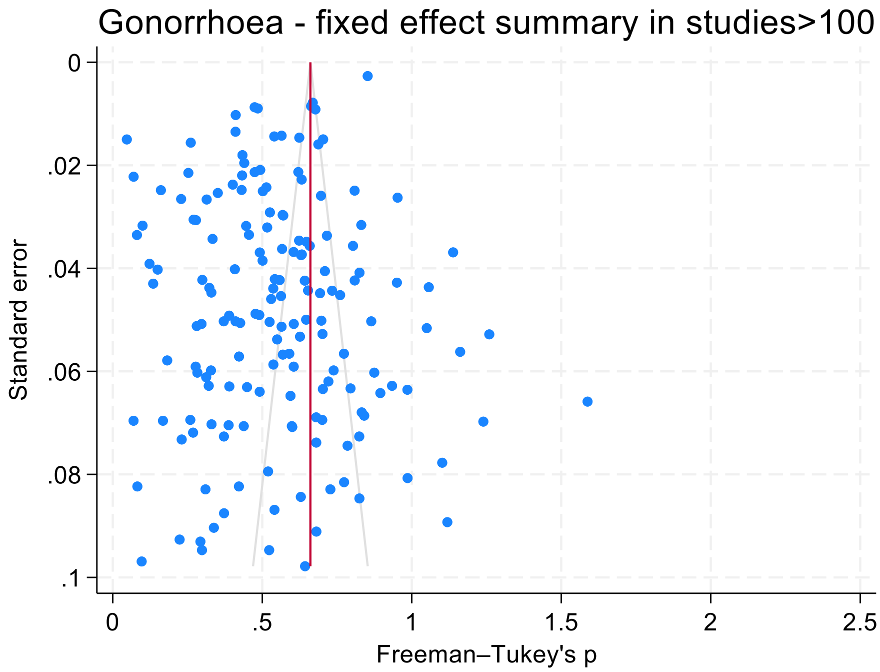

3. b) Funnel plots to assess publication bias within included chlamydia studies

(i) Random-effects using all studies

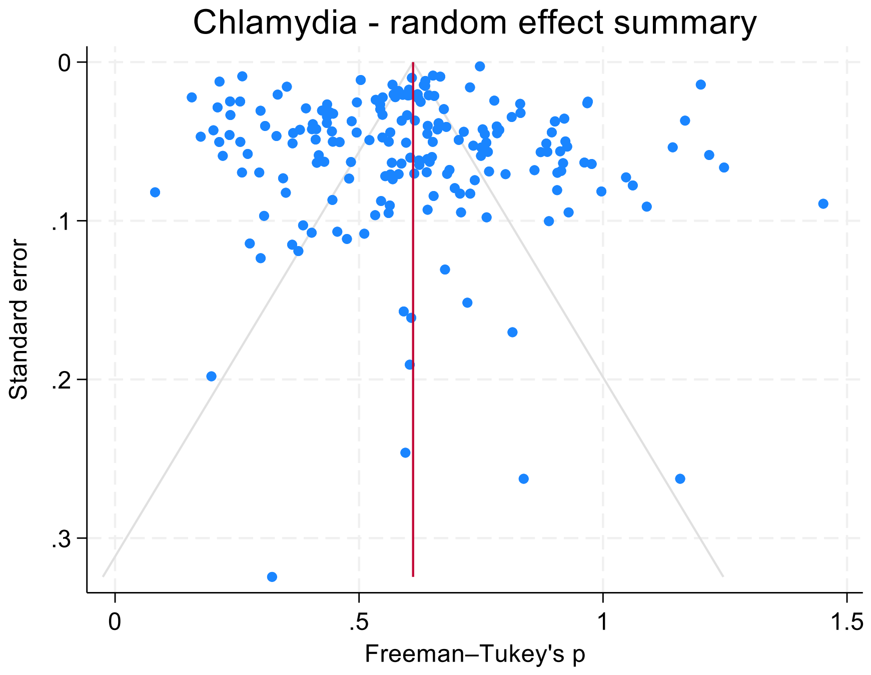

(ii) Random-effects using studies >100 tested

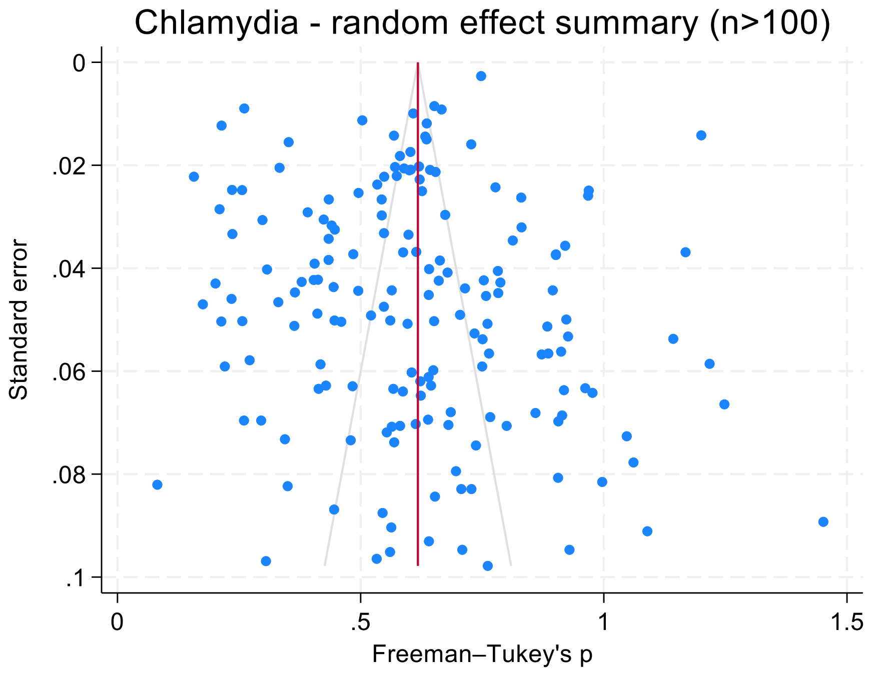

(iii) Fixed-effects using all studies

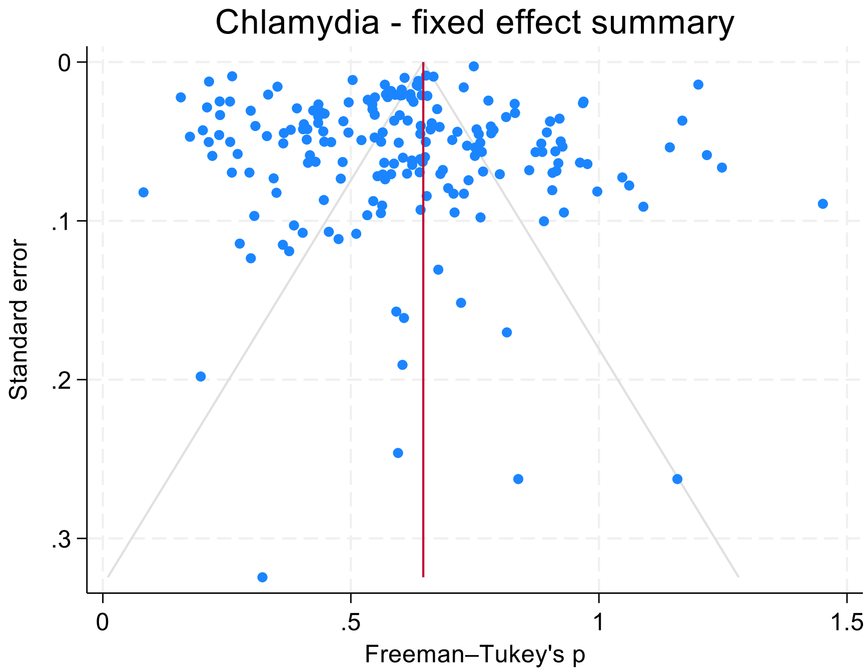

(iv) Fixed-effects using studies >100 tested

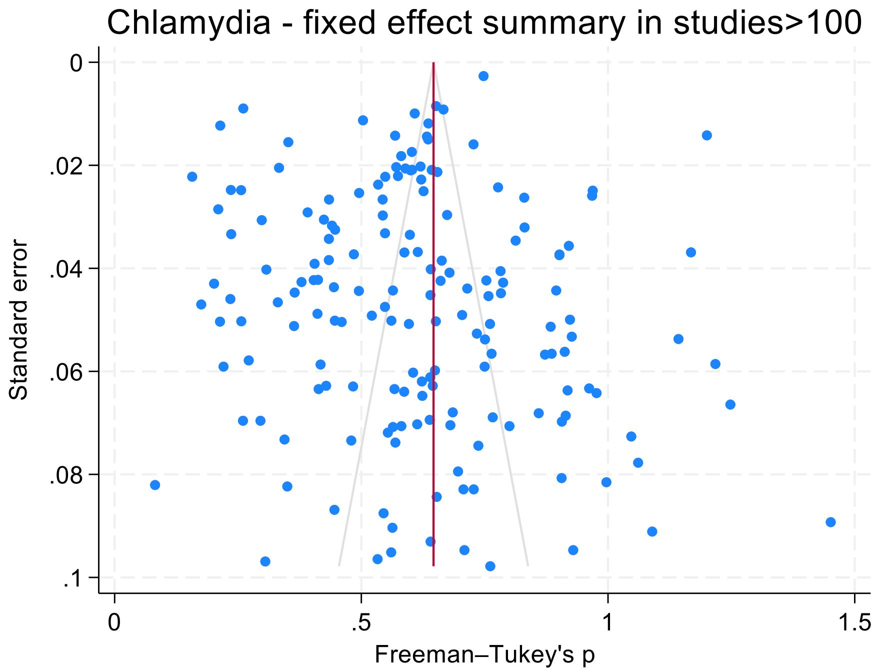

We prepared funnel plots to assess the potential for publication bias using both fixed-effects and random-effects models, due to the large variability in prevalence between regions. We did this for all studies compared with those with >100 MSM tested. We split by number of MSM to assess if studies with a larger number of MSM tested influenced publication bias. We used a transformed scale (Freeman-Tukey) to present the data in the plots for clarity. Reported point estimates were used in the assessment rather than adjusted prevalence estimates. These plots show smaller studies are more likely to show a smaller prevalence, particularly for gonorrhoea. These funnel plots support no publication bias within our study.

Supplemental File 4

1. Gonorrhoea pooled prevalence estimates: global and Sustainable Development Goal regions

1.a. Gonorrhoea global pooled prevalence estimates stratified by Sustainable Development Goal region

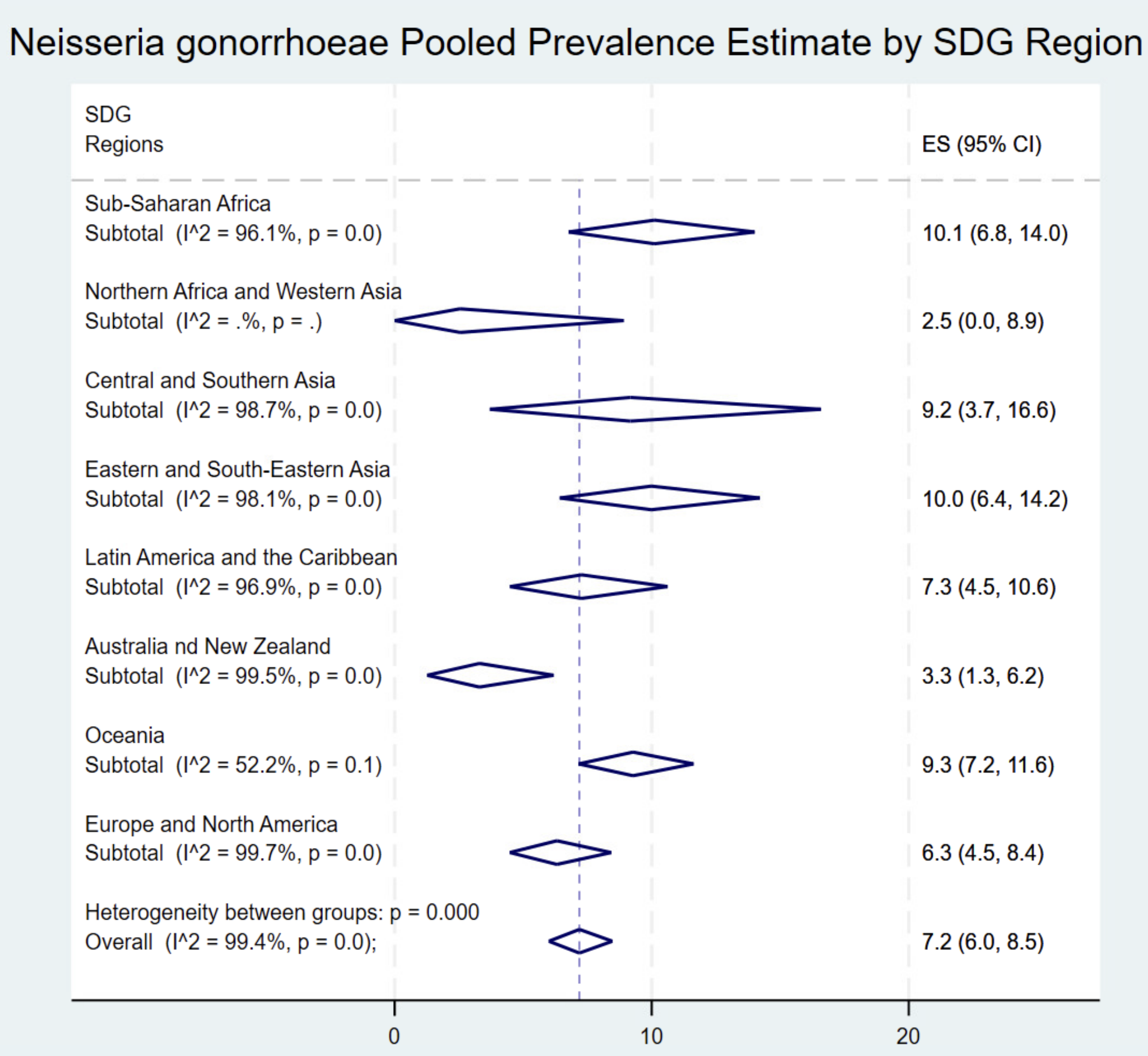

ES (95% CI) = Corrected point prevalence (95% CI); Dashed line = Pooled prevalence estimate using random effects model; Diamond = Pooled prevalence estimate and 95% CI

1.b. Sub-Saharan Africa gonorrhoea pooled prevalence estimates

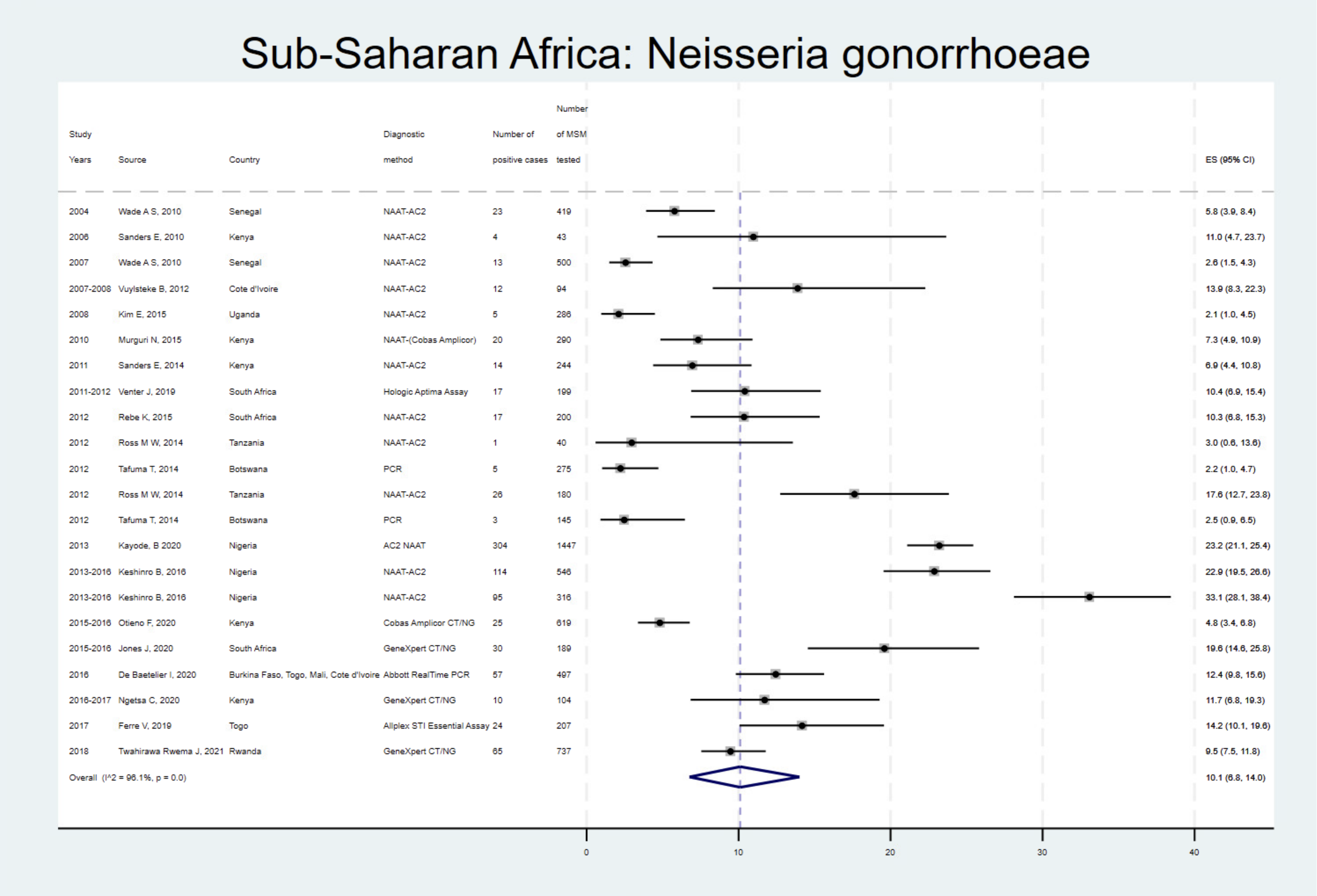

PCR = Polymerase chain reaction; NAAT = Nucleic acid amplification test; AC2 = APTIMA Combo 2; ES (95% CI) = Corrected point prevalence (95% CI); Dashed line = Pooled prevalence estimate using random effects model; Diamond = Pooled prevalence estimate and 95% CI

1.c. Northern Africa and Western Asia gonorrhoea pooled prevalence estimates

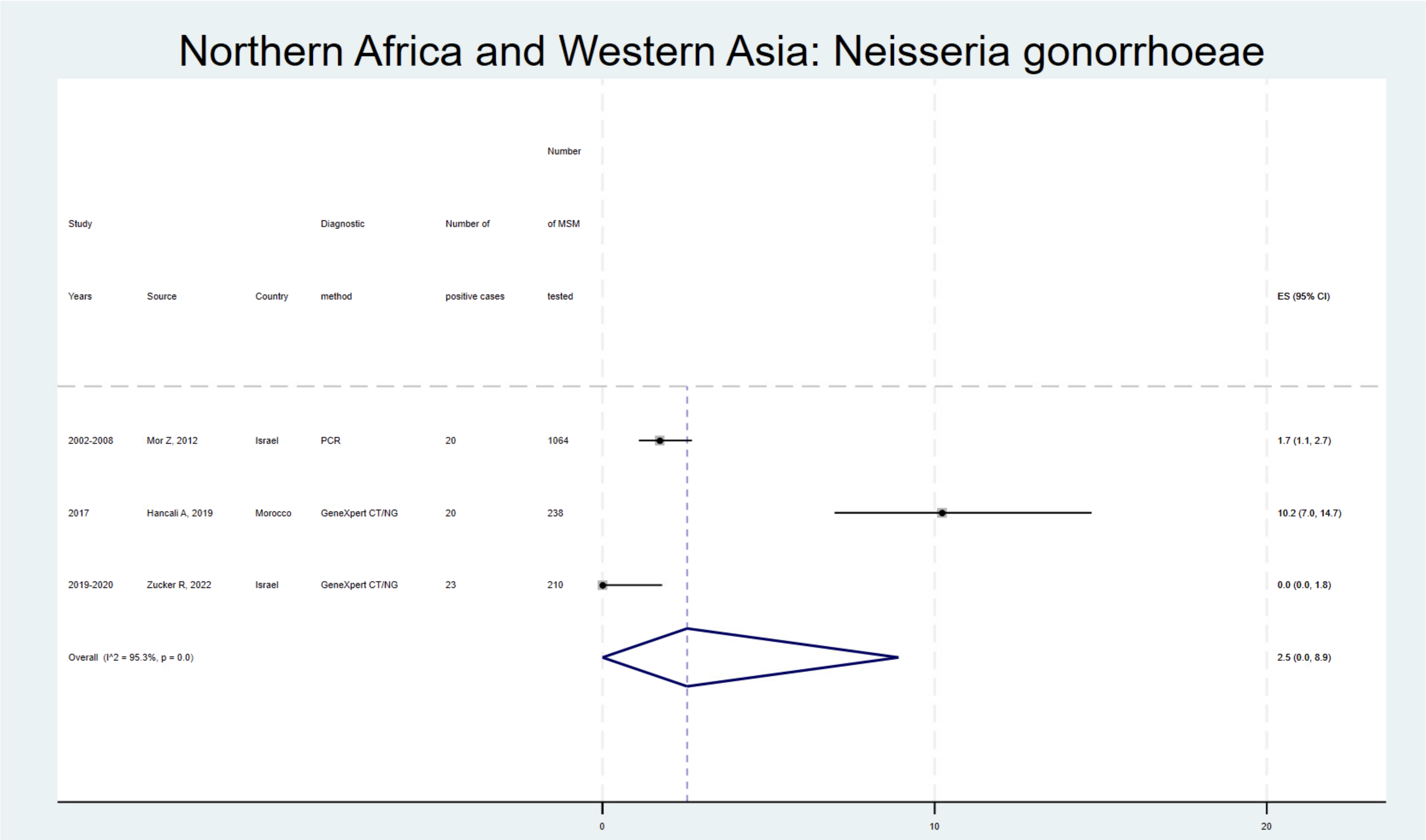

PCR = Polymerase chain reaction; NAAT = Nucleic acid amplification test; AC2 = APTIMA Combo 2; ES (95% CI) = Corrected point prevalence (95% CI); Dashed line = Pooled prevalence estimate using random effects model; Diamond = Pooled prevalence estimate and 95% CI

1.d. Central and Southern Asia gonorrhoea pooled prevalence estimates

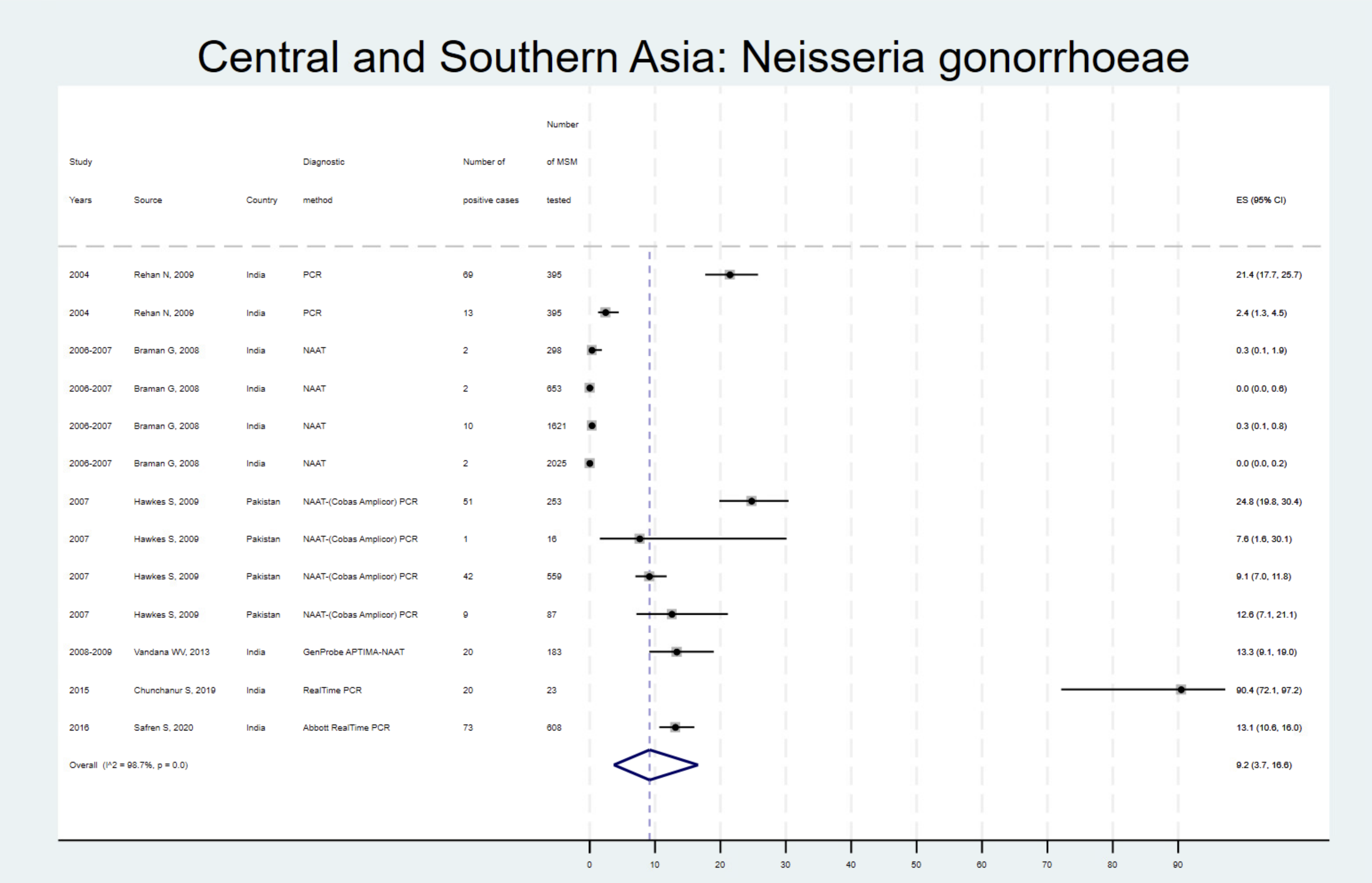

PCR = Polymerase chain reaction; NAAT = Nucleic acid amplification test; ES (95% CI) = Corrected point prevalence (95% CI); Dashed line = Pooled prevalence estimate using random effects model; Diamond = Pooled prevalence estimate and 95% CI

1.e. Eastern and South-Eastern Asia gonorrhoea pooled prevalence estimates

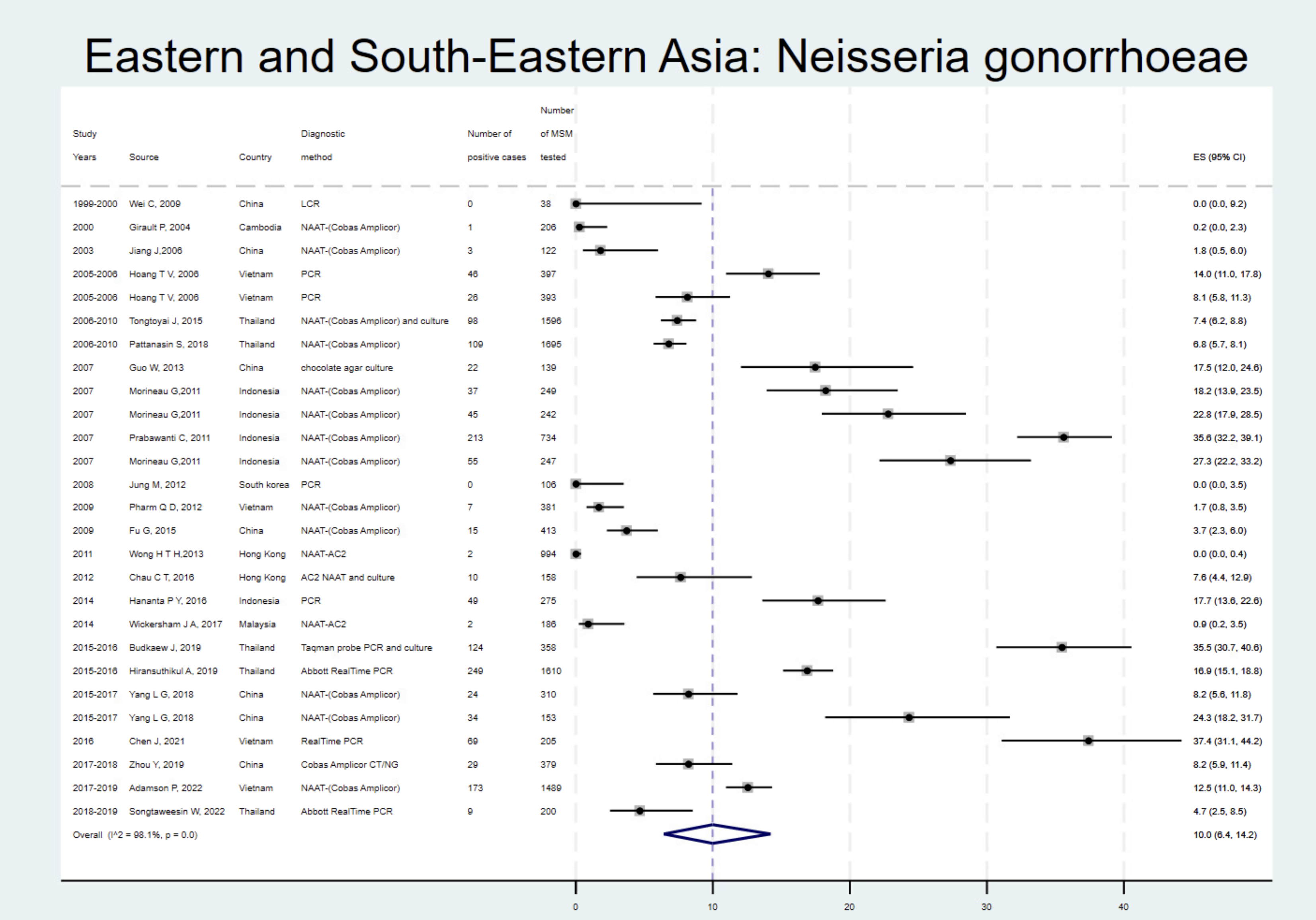

LCR = Ligase chain reaction; PCR = Polymerase chain reaction; NAAT = Nucleic acid amplification test; AC2 = APTIMA Combo 2; ES (95% CI) = Corrected point prevalence (95% CI); Dashed line = Pooled prevalence estimate using random effects model; Diamond = Pooled prevalence estimate and 95% CI

1.f. Latin America and the Caribbean gonorrhoea pooled prevalence estimates

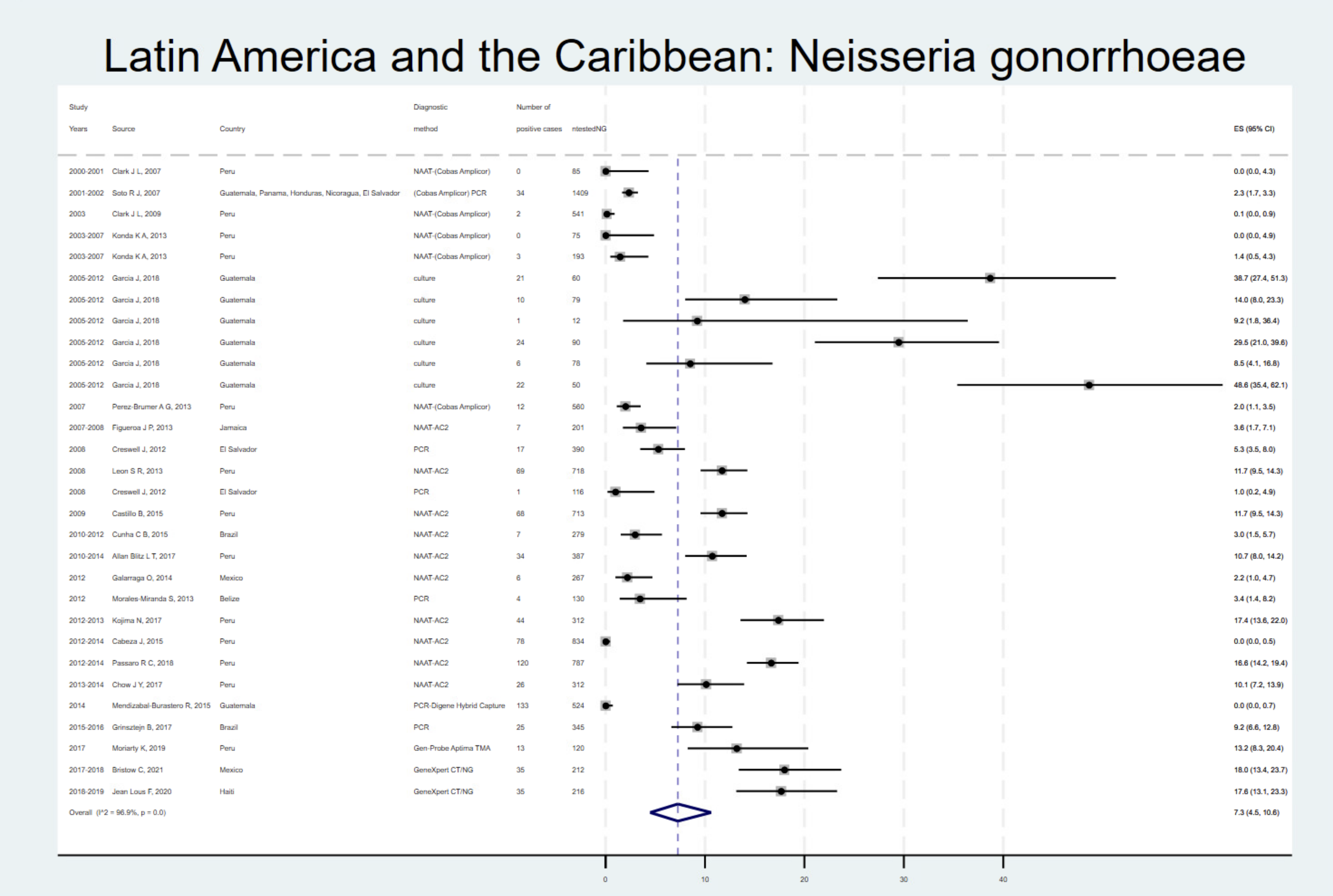

PCR = Polymerase chain reaction; NAAT = Nucleic acid amplification test; AC2 = APTIMA Combo 2; ES (95% CI) = Corrected point prevalence (95% CI); Dashed line = Pooled prevalence estimate using random effects model; Diamond = Pooled prevalence estimate and 95% CI

1.g. Australia and New Zealand gonorrhoea pooled prevalence estimates

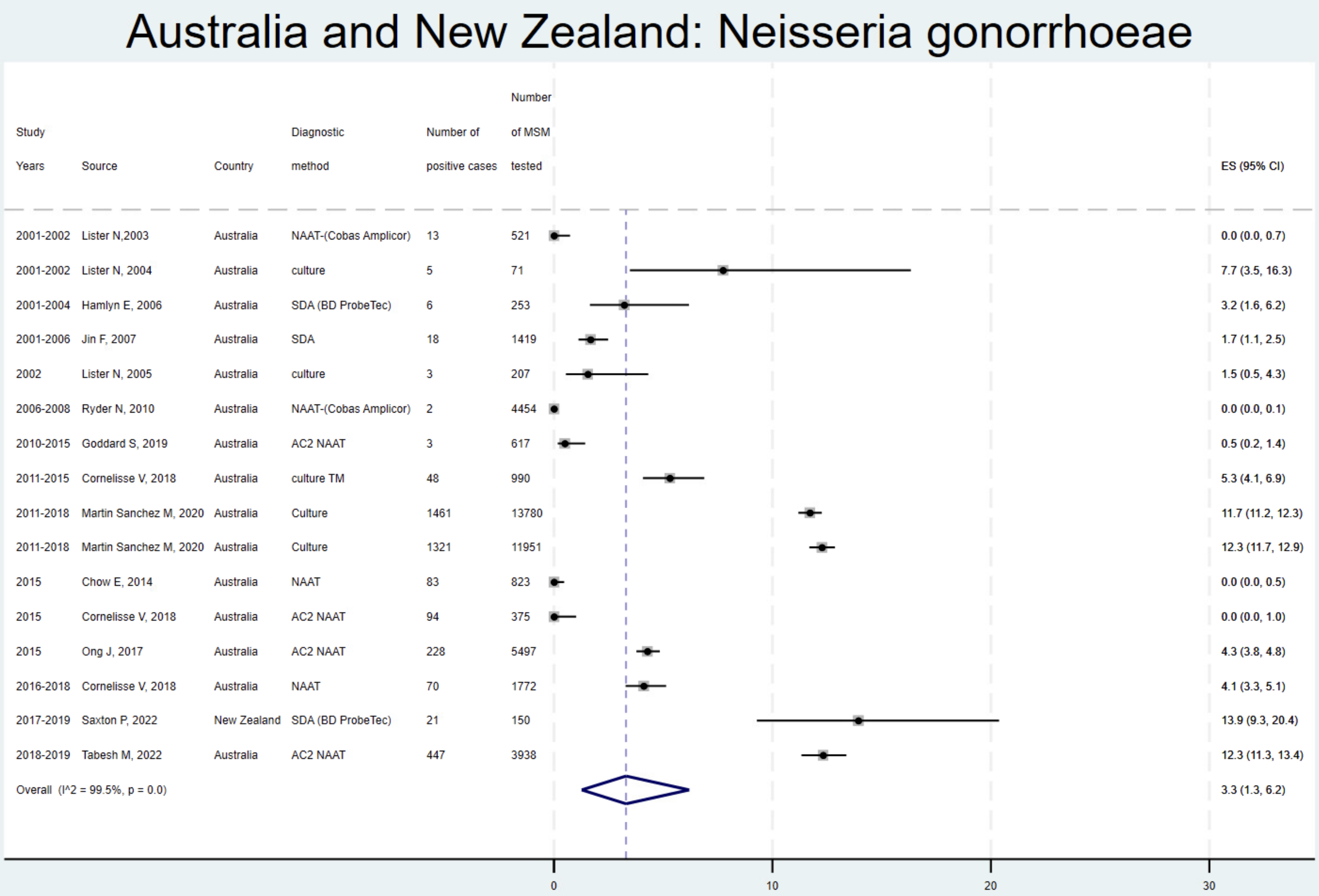

SDA = Strand displacement amplification; PCR = Polymerase chain reaction; NAAT = Nucleic acid amplification test; AC2 = APTIMA Combo 2; ES (95% CI) = Corrected point prevalence (95% CI); Dashed line = Pooled prevalence estimate using random effects model; Diamond = Pooled prevalence estimate and 95% CI

1.h. Oceania gonorrhoea pooled prevalence estimates

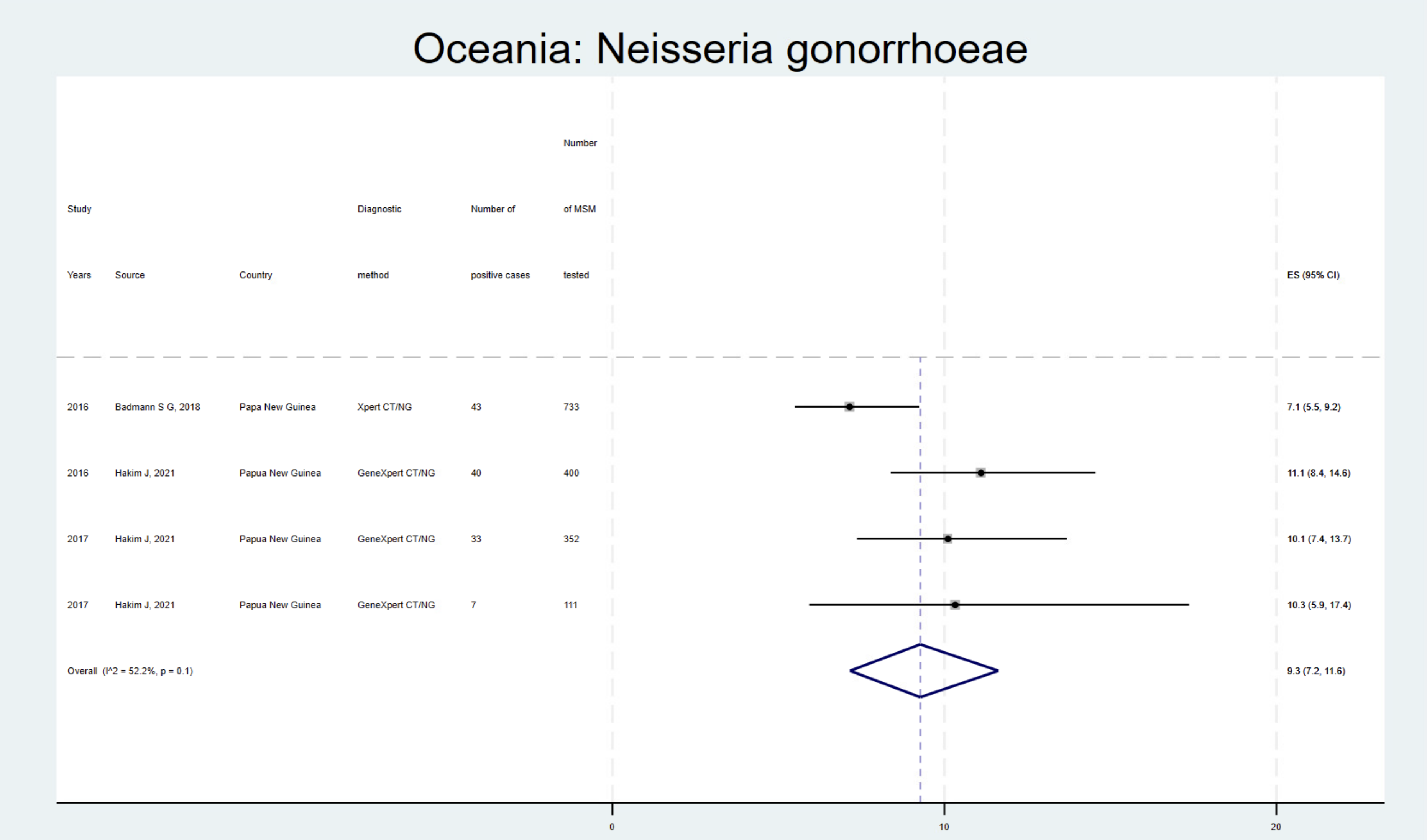

(95% CI) = Corrected point prevalence (95% CI); Dashed line = Pooled prevalence estimate using random effects model; Diamond = Pooled prevalence estimate and 95% CI

1.i. Europe and North America gonorrhoea pooled prevalence estimates

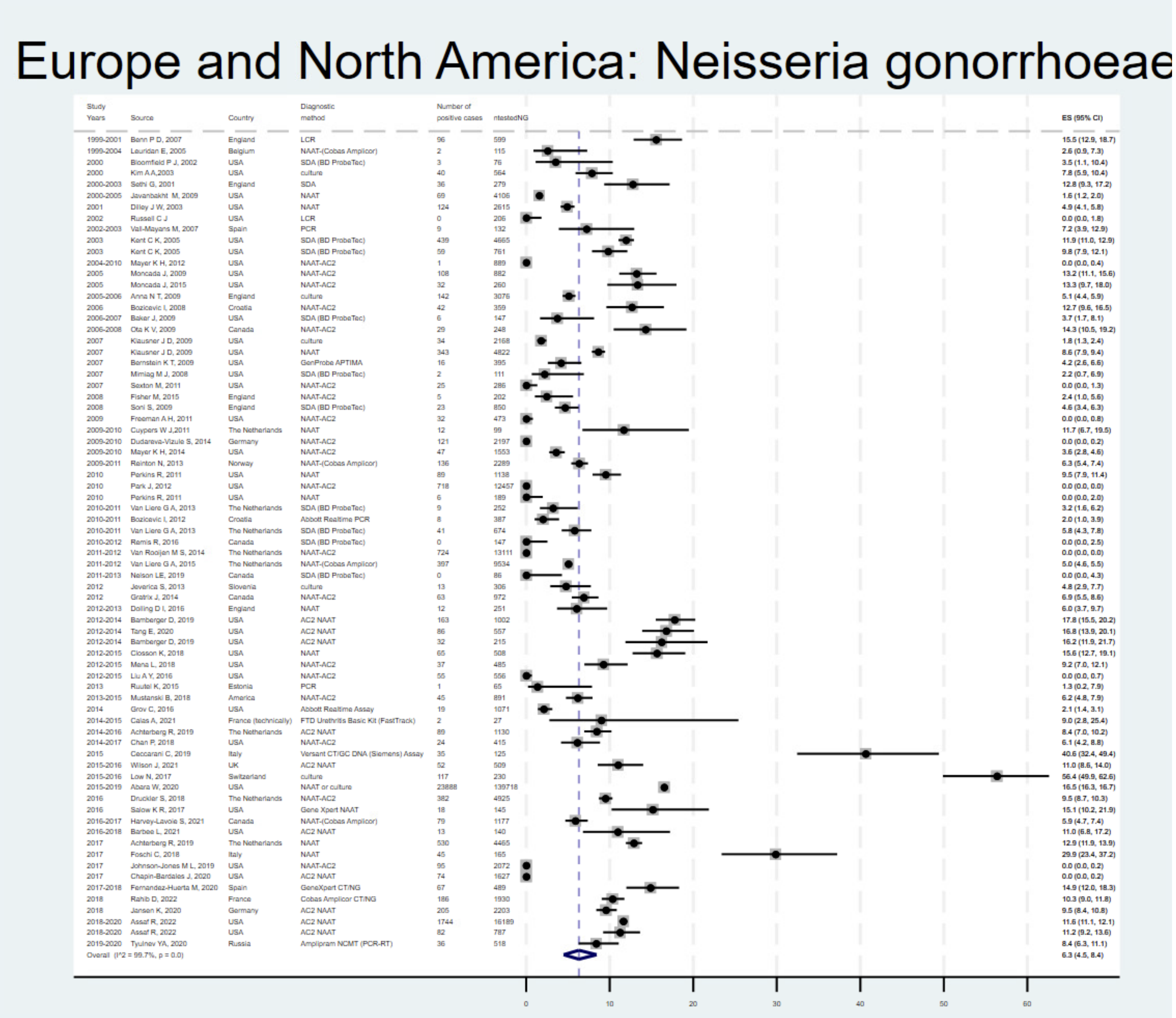

LCR = Ligase chain reaction; PCR = Polymerase chain reaction; NAAT = Nucleic acid amplification test; SDA = Strand displacement amplification; AC2 = APTIMA Combo 2; ES (95% CI) = Corrected point prevalence (95% CI); Dashed line = Pooled prevalence estimate using random effects model; Diamond = Pooled prevalence estimate and 95% CI

2. Chlamydia global and SDG regions

2.a. Chlamydia pooled prevalence estimates: global and Sustainable Development Goal regions

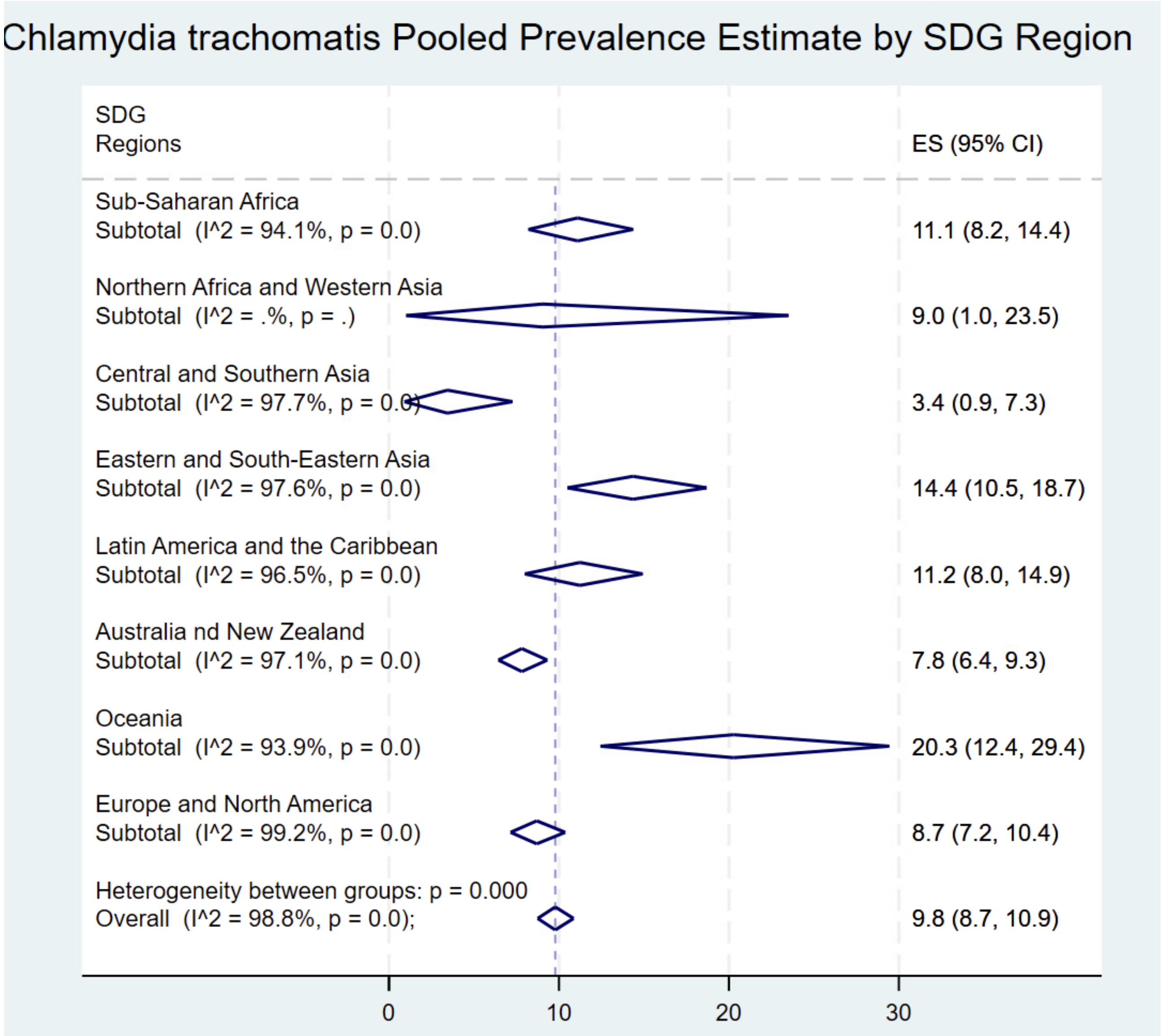

(95% CI) = Corrected point prevalence (95% CI); Dashed line = Pooled prevalence estimate using random effects model; Diamond = Pooled prevalence estimate and 95% CI

2.b. Sub-Saharan Africa chlamydia pooled prevalence estimates

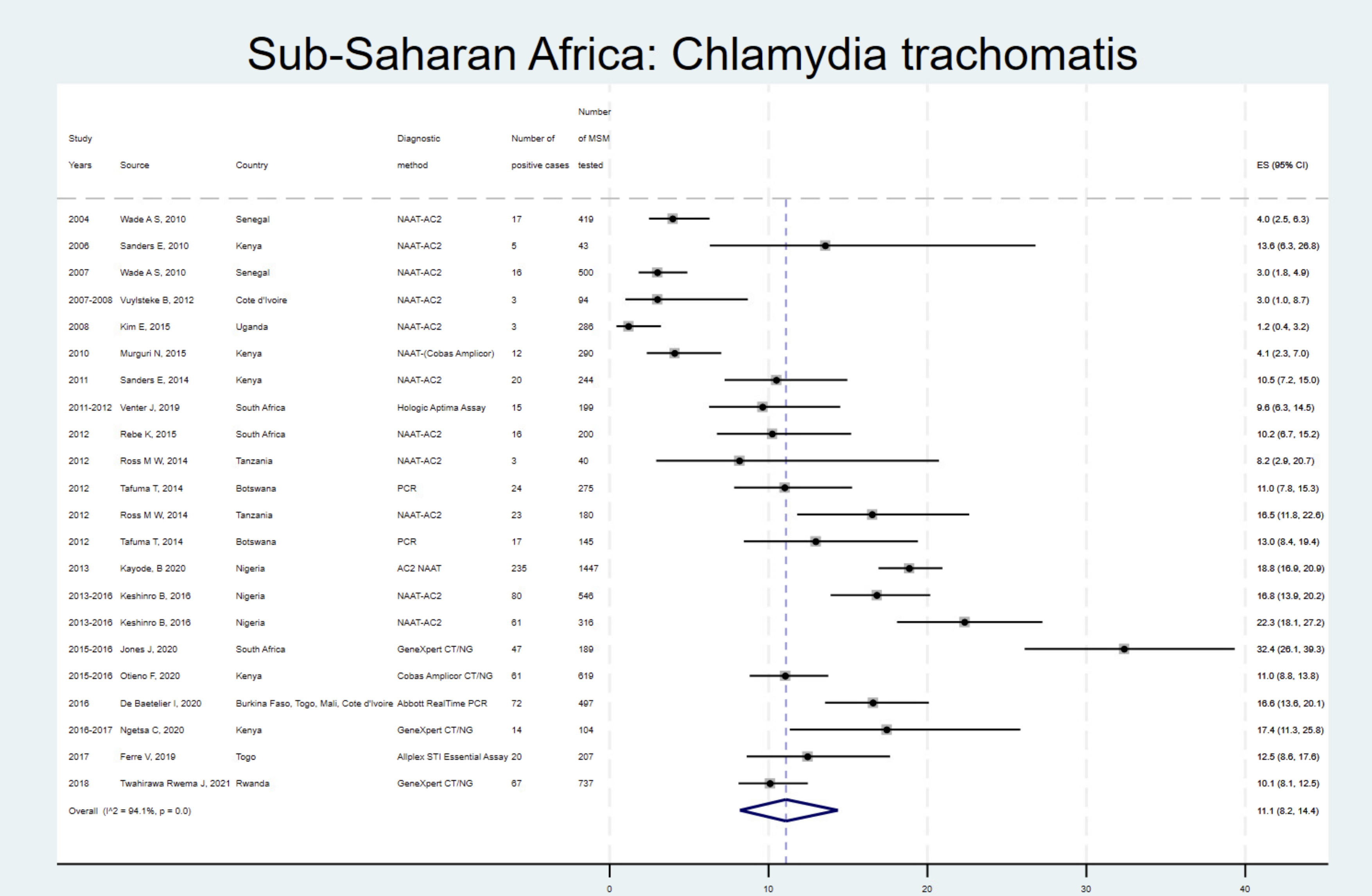

PCR = Polymerase chain reaction; NAAT = Nucleic acid amplification test; AC2 = APTIMA Combo 2; ES (95% CI) = Corrected point prevalence (95% CI); Dashed line = Pooled prevalence estimate using random effects model; Diamond = Pooled prevalence estimate and 95% CI

2.c. Northern Africa and Western Asia chlamydia pooled prevalence estimates

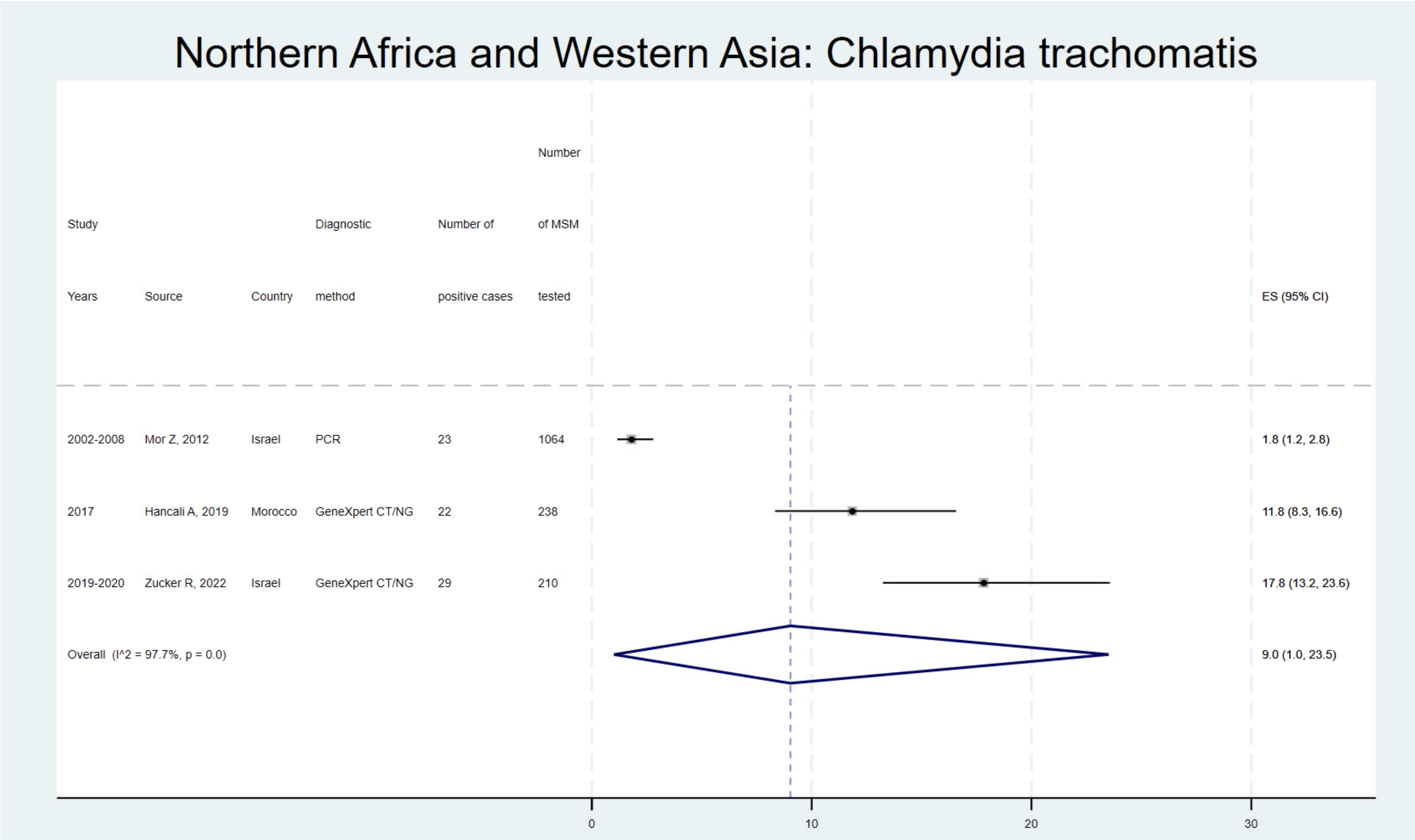

PCR = Polymerase chain reaction; ES (95% CI) = Corrected point prevalence (95% CI); Dashed line = Pooled prevalence estimate using random effects model; Diamond = Pooled prevalence estimate and 95% CI

2.d. Central and Southern Asia chlamydia pooled prevalence estimates

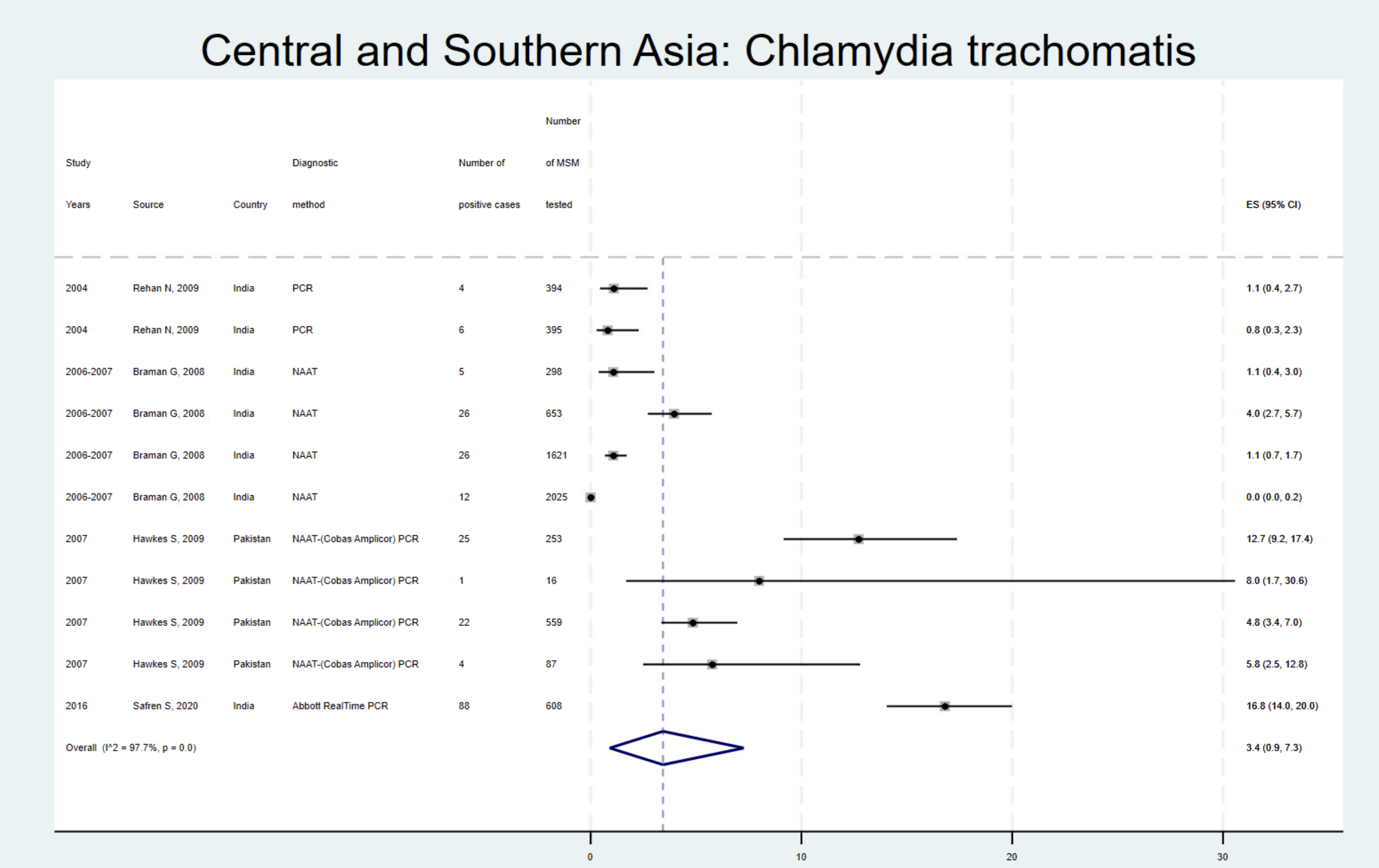

PCR = Polymerase chain reaction; NAAT = Nucleic acid amplification test; AC2 = APTIMA Combo 2; ES (95% CI) = Corrected point prevalence (95% CI); Dashed line = Pooled prevalence estimate using random effects model; Diamond = Pooled prevalence estimate and 95% CI

2.e. Eastern and South-Eastern Asia chlamydia pooled prevalence estimates

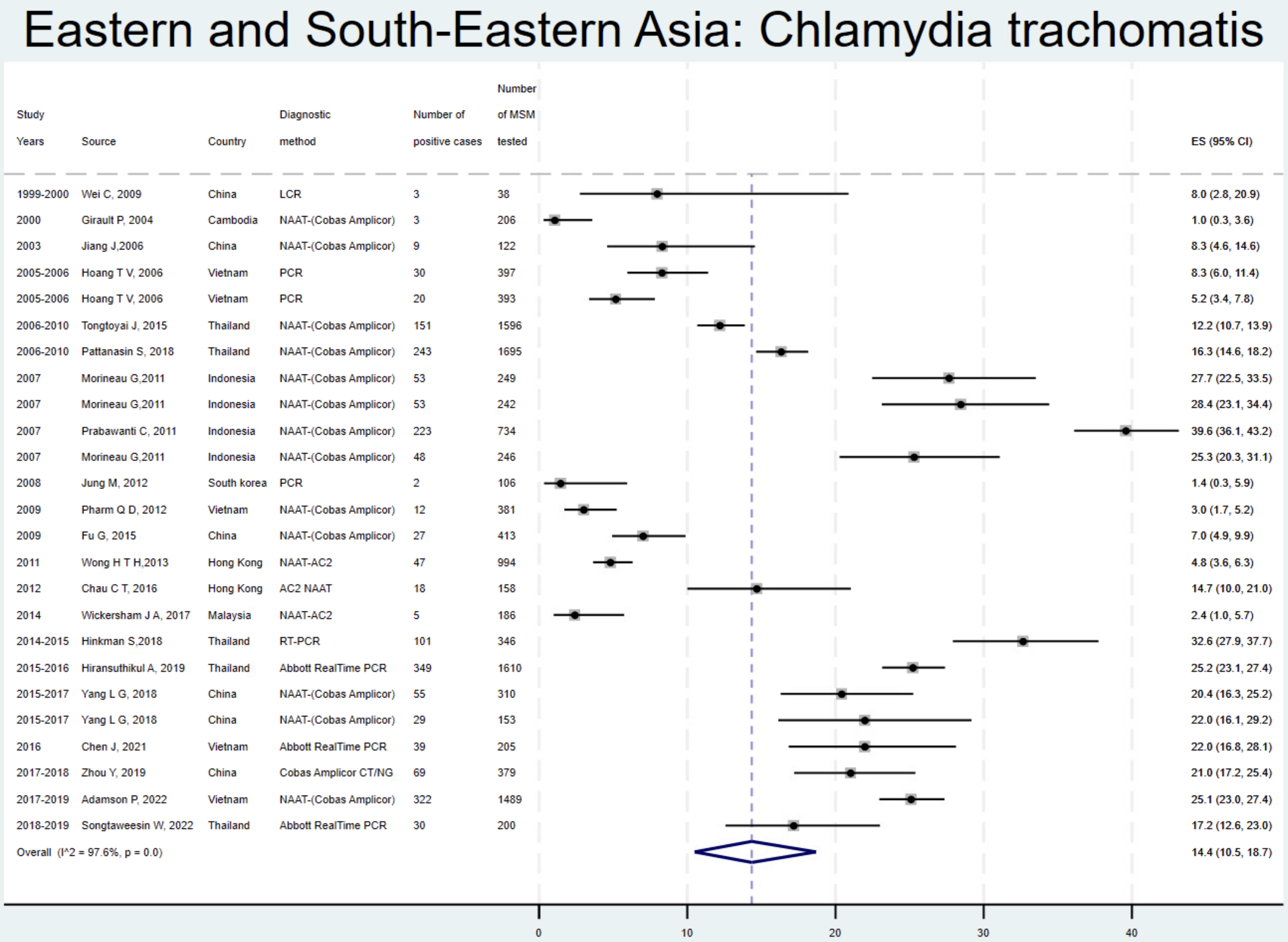

LCR = Ligase chain reaction; PCR = Polymerase chain reaction; RT-PCR = Reverse transcriptase polymerase chain reaction; NAAT = Nucleic acid amplification test; AC2 = APTIMA Combo 2; ES (95% CI) = Corrected point prevalence (95% CI); Dashed line = Pooled prevalence estimate using random effects model; Diamond=Pooled prevalence estimate and 95% CI

2.f. Latin America and the Caribbean chlamydia pooled prevalence estimates

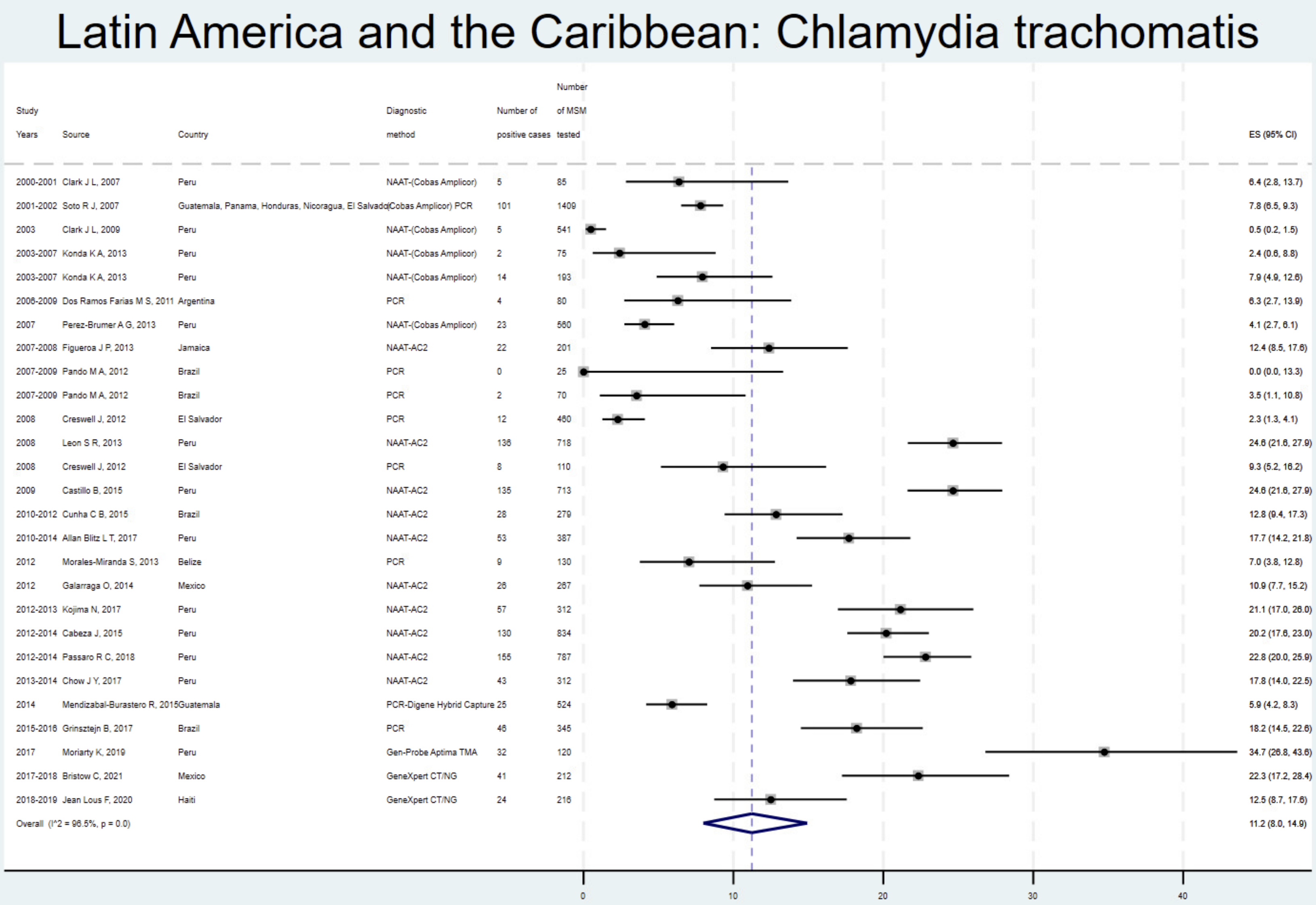

PCR = Polymerase chain reaction; NAAT = Nucleic acid amplification test; AC2 = APTIMA Combo 2; ES (95% CI) = Corrected point prevalence (95% CI); Dashed line = Pooled prevalence estimate using random effects model; Diamond = Pooled prevalence estimate and 95% CI

2.g. Australia and New Zealand chlamydia pooled prevalence estimates

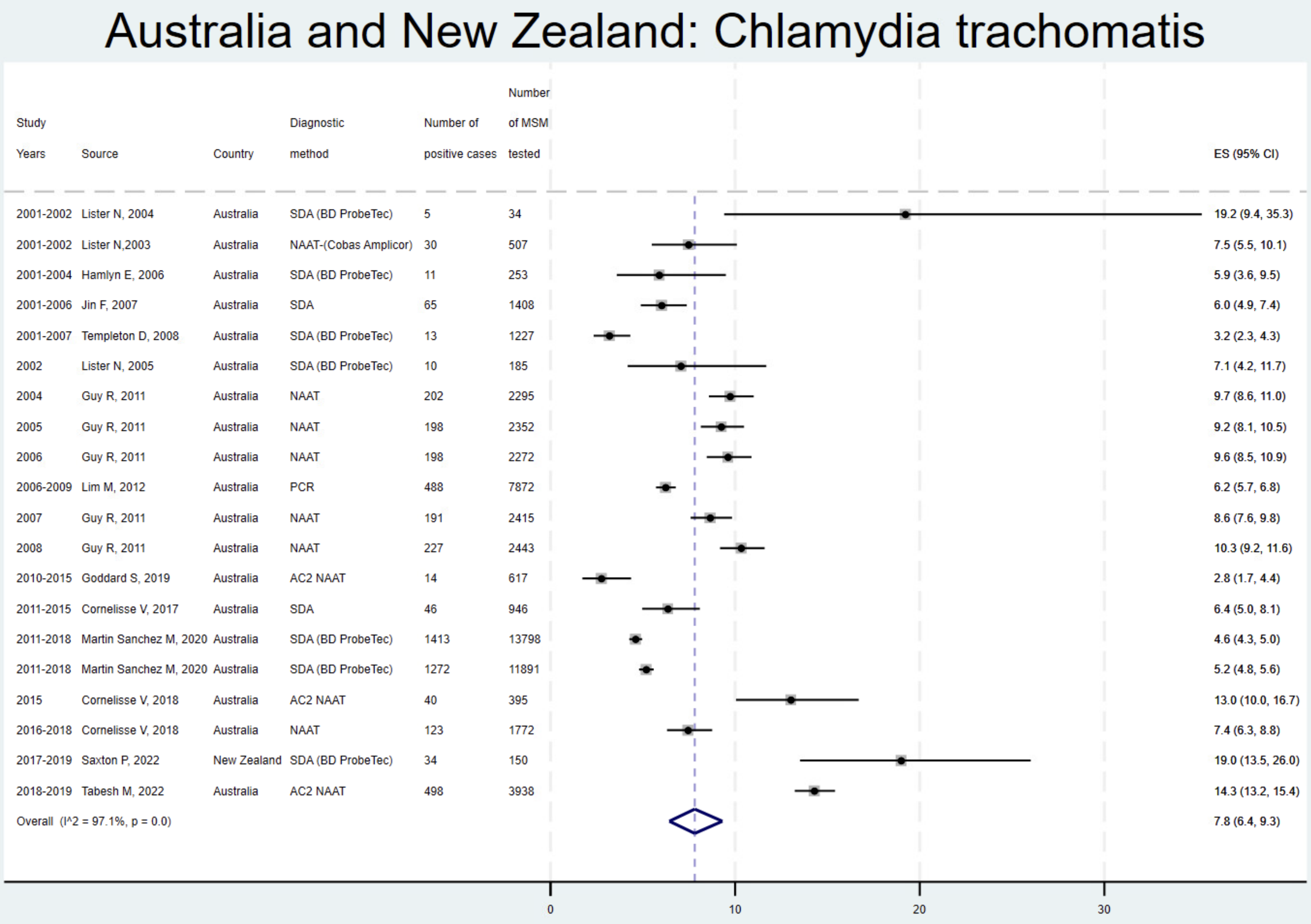

PCR = Polymerase chain reaction; NAAT = Nucleic acid amplification test; AC2 = APTIMA Combo 2; SDA = Strand displacement amplification; ES (95% CI) = Corrected point prevalence (95% CI); Dashed line = Pooled prevalence estimate using random effects model, Diamond = Pooled prevalence estimate and 95% CI

2.h. Oceania chlamydia pooled prevalence estimates

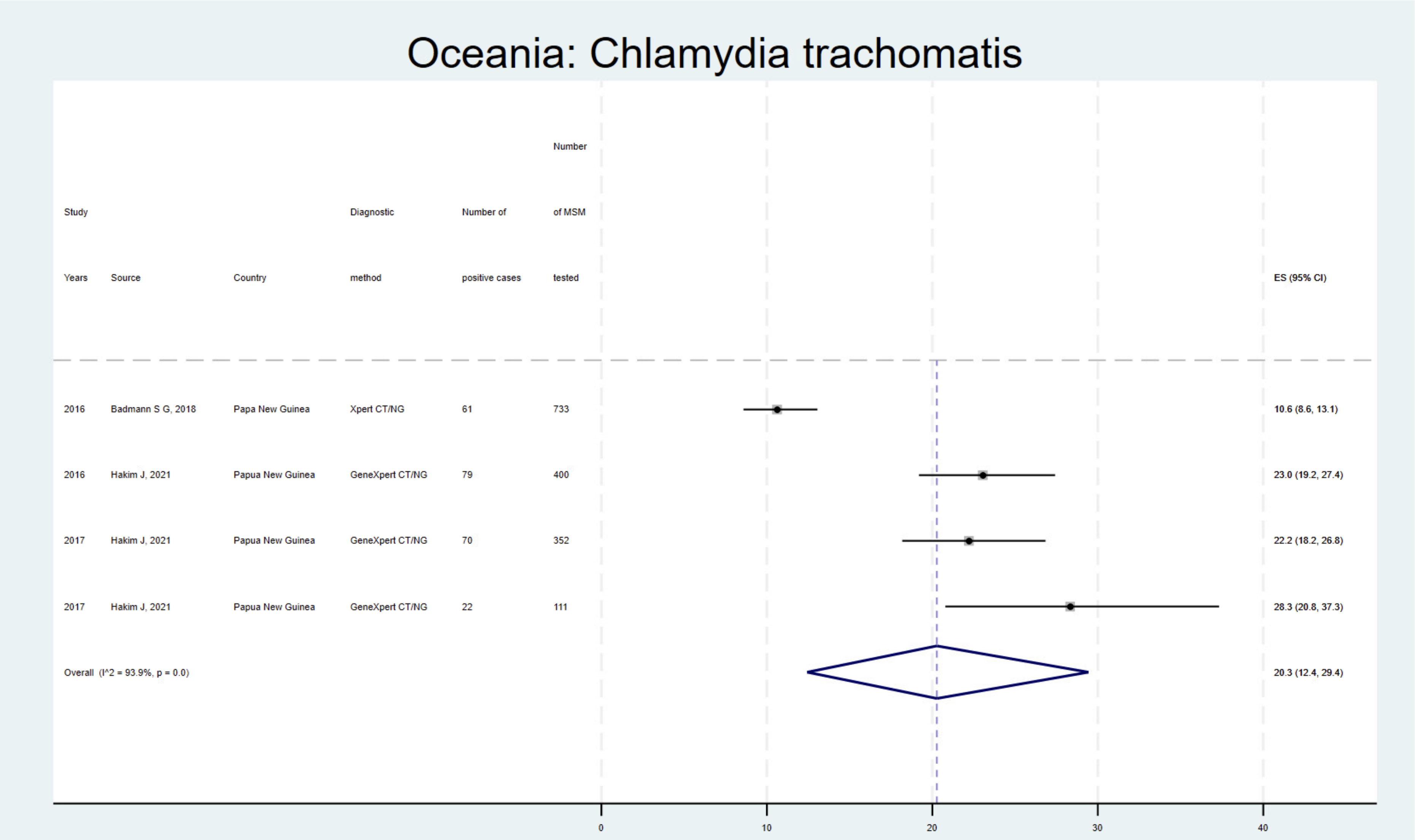

(95% CI) = Corrected point prevalence (95% CI); Dashed line = Pooled prevalence estimate using random effects model; Diamond = Pooled prevalence estimate and 95% CI

2.i. Europe and North America chlamydia pooled prevalence estimates

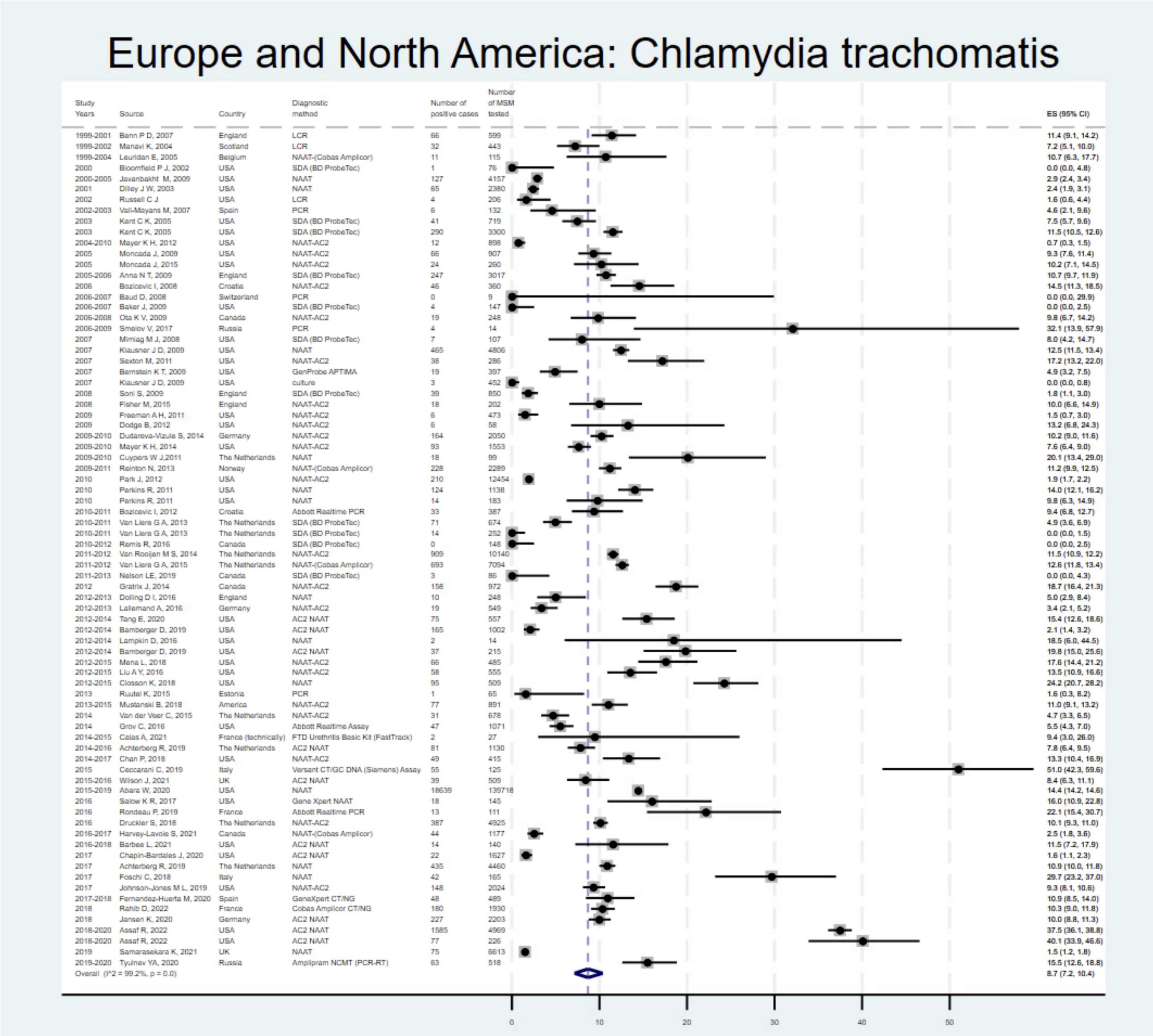

LCR = Ligase chain reaction, PCR = Polymerase chain reaction; NAAT = Nucleic acid amplification test; SDA = Strand displacement amplification; ES (95% CI) = Corrected point prevalence (95% CI); Dashed line = Pooled prevalence estimate using random effects model; Diamond = Pooled prevalence estimate and 95% CI

Supplemental File 5

1. Gonorrhoea and chlamydia: Subgroup analysis of prevalence among men who have sex with men point estimates included in the meta-analysis.

Region	MSM population		Study type		Study setting		Time period		Anatomical Site		AXIS score		Sample size		Age	
	MSW, TGW, and/or TGWSW only	Other MSM studies	Cross-sectional	Other study type	Clinic	Community or community and clinic	2000-2010	2011-2022	Genital and pharyngeal	Rectal	High risk of bias	Low risk of bias	≤ 500	> 500	≤ Median age	> Median age
**Gonorrhoea**																
Sub-Saharan Africa (18 studies)	-		▼	▲	●		●		●		●		●		-	
Northern Africa and Western Asia (3 studies)	-		-		-		-		-		-		-		-	
Central and Southern Asia (6 studies)	●		-		▲	▼	▼	▲	▼	▲	-		●		-	
Eastern and South-Eastern Asia (23 studies)	▲	▼	●		●		●		●		●		●		●	
Latin America and the Caribbean (23 studies)	●		●		●		▼	▲	▼	▲	●		●		●	
Australia and New Zealand (15 studies)	-		●		●		●		▼	▲	-		●		●	
Oceania (2 studies)	-		-		-		-		-		-		-		-	
Europe and North America (67 studies)	●		▼	▲	▲	▼	▼	▲	▼	▲	●		●		●	
**Chlamydia**																
Sub-Saharan Africa (18 studies)	-		▼	▲	●		●		●		●		●		-	
Northern Africa and Western Asia (3 studies)	-		-		-		-		-		-		-		-	
Central and Southern Asia (5 studies)	●		-		●		●		●		-		●		-	
Eastern and South-Eastern Asia (22 studies)	▲	▼	●		●		●		●		●		●		●	
Latin America and the Caribbean (24 studies)	●		●		●		●		▼	▲	●		●		●	
Australia and New Zealand (15 studies)	-		●		▲	▼	●		●		-		●		●	
Oceania (2 studies)	-		-		-		-		-		-		-		-	
Europe and North America (73 studies)	●		●		●		●		▼	▲	●		●		●	

● = Subgroup pooled prevalence estimate for which there is no evidence of effect on the overall or SDG pooled prevalence estimates.

▲▼ = Direction of individual stratified effect compared to overall pooled prevalence, p<0.001.

- = <2 data points for one of the individual subgroup categories and therefore insufficient data points to make a pooled estimate and for subgroup comparison.

Supplemental File 6

1. PRISMA 2020 checklist

Section and Topic	Item #	Checklist item	Location where item is reported (page)
**TITLE**			
Title	1	Identify the report as a systematic review.	1, Title page
**ABSTRACT**			
Abstract	2	See the PRISMA 2020 for Abstracts checklist.	1
**INTRODUCTION**			
Rationale	3	Describe the rationale for the review in the context of existing knowledge.	2
Objectives	4	Provide an explicit statement of the objective(s) or question(s) the review addresses.	2
**METHODS**			
Eligibility criteria	5	Specify the inclusion and exclusion criteria for the review and how studies were grouped for the syntheses.	3, Supplementary file: 1.3
Information sources	6	Specify all databases, registers, websites, organisations, reference lists and other sources searched or consulted to identify studies. Specify the date when each source was last searched or consulted.	3
Search strategy	7	Present the full search strategies for all databases, registers and websites, including any filters and limits used.	3, Supplementary file: 1.1, 1.2
Selection process	8	Specify the methods used to decide whether a study met the inclusion criteria of the review, including how many reviewers screened each record and each report retrieved, whether they worked independently, and if applicable, details of automation tools used in the process.	3-4
Data collection process	9	Specify the methods used to collect data from reports, including how many reviewers collected data from each report, whether they worked independently, any processes for obtaining or confirming data from study investigators, and if applicable, details of automation tools used in the process.	4
Data items	10a	List and define all outcomes for which data were sought. Specify whether all results that were compatible with each outcome domain in each study were sought (e.g. for all measures, time points, analyses), and if not, the methods used to decide which results to collect.	4
	10b	List and define all other variables for which data were sought (e.g. participant and intervention characteristics, funding sources). Describe any assumptions made about any missing or unclear information.	4
Study risk of bias assessment	11	Specify the methods used to assess risk of bias in the included studies, including details of the tool(s) used, how many reviewers assessed each study and whether they worked independently, and if applicable, details of automation tools used in the process.	4
Effect measures	12	Specify for each outcome the effect measure(s) (e.g. risk ratio, mean difference) used in the synthesis or presentation of results.	4-5

Section and Topic	Item #	Checklist item	Location where item is reported (page)
Synthesis methods	13a	Describe the processes used to decide which studies were eligible for each synthesis (e.g. tabulating the study intervention characteristics and comparing against the planned groups for each synthesis (item #5)).	4
	13b	Describe any methods required to prepare the data for presentation or synthesis, such as handling of missing summary statistics, or data conversions.	4
	13c	Describe any methods used to tabulate or visually display results of individual studies and syntheses.	4
	13d	Describe any methods used to synthesize results and provide a rationale for the choice(s). If meta-analysis was performed, describe the model(s), method(s) to identify the presence and extent of statistical heterogeneity, and software package(s) used.	4-5
	13e	Describe any methods used to explore possible causes of heterogeneity among study results (e.g. subgroup analysis, meta-regression).	4-5
	13f	Describe any sensitivity analyses conducted to assess robustness of the synthesized results.	4-5
Reporting bias assessment	14	Describe any methods used to assess risk of bias due to missing results in a synthesis (arising from reporting biases).	4
Certainty assessment	15	Describe any methods used to assess certainty (or confidence) in the body of evidence for an outcome.	4-5
**RESULTS**			
Study selection	16a	Describe the results of the search and selection process, from the number of records identified in the search to the number of studies included in the review, ideally using a flow diagram.	Figure 1, 5-6
	16b	Cite studies that might appear to meet the inclusion criteria, but which were excluded, and explain why they were excluded.	Figure 1
Study characteristics	17	Cite each included study and present its characteristics.	5-6, Supplementary file: 2
Risk of bias in studies	18	Present assessments of risk of bias for each included study.	9, 11-12, Supplementary file: 2
Results of individual studies	19	For all outcomes, present, for each study: (a) summary statistics for each group (where appropriate) and (b) an effect estimate and its precision (e.g. confidence/credible interval), ideally using structured tables or plots.	Supplementary file: 2
Results of syntheses	20a	For each synthesis, briefly summarise the characteristics and risk of bias among contributing studies.	9-11
	20b	Present results of all statistical syntheses conducted. If meta-analysis was done, present for each the summary estimate and its precision (e.g. confidence/credible interval) and measures of statistical heterogeneity. If comparing groups, describe the direction of the effect.	Table 1 and 2, Figure 2, 6-1, Supplementary file: 4
	20c	Present results of all investigations of possible causes of heterogeneity among study results.	6, 8-10, Supplementary file: 4

Section and Topic	Item #	Checklist item	Location where item is reported (page)
	20d	Present results of all sensitivity analyses conducted to assess the robustness of the synthesized results.	8-10, Supplementary file: 5
Reporting biases	21	Present assessments of risk of bias due to missing results (arising from reporting biases) for each synthesis assessed.	6, 8-9
Certainty of evidence	22	Present assessments of certainty (or confidence) in the body of evidence for each outcome assessed.	Figure 2, 8-9
**DISCUSSION**			
Discussion	23a	Provide a general interpretation of the results in the context of other evidence.	10-12
	23b	Discuss any limitations of the evidence included in the review.	11-12
	23c	Discuss any limitations of the review processes used.	11-12
	23d	Discuss implications of the results for practice, policy, and future research.	11-12
**OTHER INFORMATION**			
Registration and protocol	24a	Provide registration information for the review, including register name and registration number, or state that the review was not registered.	3
	24b	Indicate where the review protocol can be accessed, or state that a protocol was not prepared.	3
	24c	Describe and explain any amendments to information provided at registration or in the protocol.	3
Support	25	Describe sources of financial or non-financial support for the review, and the role of the funders or sponsors in the review.	12
Competing interests	26	Declare any competing interests of review authors.	13
Availability of data, code and other materials	27	Report which of the following are publicly available and where they can be found: template data collection forms; data extracted from included studies; data used for all analyses; analytic code; any other materials used in the review.	13

From: Page MJ, McKenzie JE, Bossuyt PM, Boutron I, Hoffmann TC, Mulrow CD, et al. The PRISMA 2020 statement: an updated guideline for reporting systematic reviews. BMJ 2021;372:n71. doi: 10.1136/bmj.n71

For more information, visit: http://www.prisma-statement.org/

References

1. World Health Organization. Laboratory tests for the detection of reproductive tract infections. *World Health Organisation* 1999
2. Cook R, Hutchison S, Østergaard L, et al. Systematic Review: Noninvasive Testing for *Chlamydia trachomatis* and *Neisseria gonorrhoeae*. *Annals of internal medicine* 2005;142:914-25.
3. Moncada J, Schachter J, Liska S, et al. Evaluation of self-collected glans and rectal swabs from men who have sex with men for detection of Chlamydia trachomatis and Neisseria gonorrhoeae by use of nucleic acid amplification tests. *Journal of Clinical Microbiology* 2009;47(6):1657-62.
4. Van Der Pol B, Taylor SN, Lebar W, et al. Clinical evaluation of the BD ProbeTec™ *Neisseria gonorrhoeae* Qx amplified DNA assay on the BD Viper™ system with XTR™ technology. *Sexually transmitted diseases* 2012;39(2):147-53. doi: 10.1097/OLQ.0b013e3182372fd8
5. Schachter J, Moncada J, Liska S, et al. Nucleic acid amplification tests in the diagnosis of chlamydial and gonococcal infections of the oropharynx and rectum in men who have sex with men. *Sex Transm Dis* 2008;35(7):637-42. doi: 10.1097/OLQ.0b013e31817bdd7e [published Online First: 2008/06/04]
6. Jin F, Imrie J, Prestage GP, et al. Prevalence, incidence and risk factors for pharyngeal chlamydia in the community based Health in Men (HIM) cohort of homosexual men in Sydney, Australia. *Sexually Transmitted Infections* 2008;84(5):361-63.
7. Meehan M, Wawer M, Serwadda D, et al. Laboratory methods for the diagnosis of reproductive tract infections and selected conditions in population-based studies. In: Elias C, Koenig M, Jejeebhoy S, eds. Investigating Reproductive Tract Infections and Other Gynaecological Disorders: A Multidisciplinary Research Approach. Cambridge: Cambridge University Press 2003:261-82.
8. Schwebke JR, Lawing LF. Improved detection by DNA amplification of *Trichomonas vaginalis* in males. *J Clin Microbiol* 2002;40(10):3681-3. doi: 10.1128/jcm.40.10.3681-3683.2002 [published Online First: 2002/10/02]
9. Sanders EJ, Thiong'o AN, Okuku HS, et al. High prevalence of *Chlamydia trachomatis* and *Neisseria gonorrhoeae* infections among HIV-1 negative men who have sex with men in coastal Kenya. *Sexually Transmitted Infections* 2010;86(6):440-41.
10. Wade AS, Larmarange J, Diop AK, et al. Reduction in risk-taking behaviors among MSM in Senegal between 2004 and 2007 and prevalence of HIV and other STIs. ELIHoS Project, ANRS 12139. *AIDS Care - Psychological and Socio-Medical Aspects of AIDS/HIV* 2010;22(4):409-14.
11. Vuylsteke B, Semde G, Sika L, et al. High prevalence of HIV and sexually transmitted infections among male sex workers in Abidjan, Cote d'Ivoire: Need for services tailored to their needs. *Sexually Transmitted Infections* 2012;88(4):288-93.
12. Kim EJ, Hladik W, Barker J, et al. Sexually transmitted infections associated with alcohol use and HIV infection among men who have sex with men in Kampala, Uganda. *Sexually Transmitted Infections* 2016;92(3):240-45.
13. Muraguri N, Tun W, Okal J, et al. HIV and STI prevalence and risk factors among male sex workers and other men who have sex with men in nairobi, kenya. *Journal of Acquired Immune Deficiency Syndromes* 2015;68(1):91-96.
14. Sanders EJ, Wahome E, Okuku HS, et al. Evaluation of WHO screening algorithm for the presumptive treatment of asymptomatic rectal gonorrhoea and chlamydia infections in at-risk MSM in Kenya. *Sexually Transmitted Infections* 2014;90(2):94-99.
15. Rebe K, Lewis D, Myer L, et al. A cross sectional analysis of gonococcal and chlamydial infections among men-who-have-sex-with-men in Cape Town, South Africa. *PLoS ONE* 2015;10 (9)(e0138315)
16. Ross MW, Nyoni J, Ahaneku HO, et al. High HIV seroprevalence, rectal STIs and riskxy sexual behaviour in men who have sex with men in Dar es Salaam and Tanga, Tanzania. *BMJ Open* 2014;4 (8) (e006175)
17. Tafuma T, Merrigan MB, Okui LA, et al. HIV/sexually transmitted infection prevalence and sexual behavior of men who have sex with men in 3 districts of botswana: Results from the 2012 biobehavioral survey. *Sexually Transmitted Diseases* 2014;41(8):480-85.
18. Venter JME, Mahlangu PM, Muller EE, et al. Comparison of an in-house real-time duplex PCR assay with commercial HOLOGIC APTIMA assays for the detection of *Neisseria gonorrhoeae* and *Chlamydia trachomatis* in urine and extra-genital specimens. *BMC Infectious Diseases* 2019;19(1) doi: 10.1186/s12879-018-3629-0
19. Kayode BO, Mitchell A, Ndembi N, et al. Retention of a cohort of men who have sex with men and transgender women at risk for and living with HIV in Abuja and Lagos, Nigeria: a longitudinal analysis. *Journal of the International AIDS Society* 2020;23(S6) doi: 10.1002/jia2.25592
20. Keshinro B, Crowell TA, Nowak RG, et al. High prevalence of HIV, chlamydia and gonorrhoea among men who have sex with men and transgender women attending trusted community centres in Abuja and Lagos, Nigeria. *Journal of the International AIDS Society* 2016;19 (1) (21270)
21. De Baetselier I, Vuylsteke B, Yaya I, et al. To pool or not to pool samples for sexually transmitted infections detection in men who have sex with men? An evaluation of a new pooling method using the genexpert instrument in West Africa. *Sexually Transmitted Diseases* 2020;47(8):556-61. doi: 10.1097/OLQ.0000000000001191
22. Jones J, Sanchez TH, Dominguez K, et al. Sexually transmitted infection screening, prevalence and incidence among South African men and transgender women who have sex with men enrolled in a combination HIV prevention cohort study: the Sibanye Methods for Prevention Packages Programme (MP3) project. *Journal of the International AIDS Society* 2020;23(S6) doi: 10.1002/jia2.25594
23. Ngetsa CJ, Heymann MW, Thiong'o A, et al. Rectal gonorrhoea and chlamydia among men who have sex with men in coastal Kenya. *Wellcome Open Research*;4:79.
24. Otieno F, Ng'ety G, Okall D, et al. Incident gonorrhoea and chlamydia among a prospective cohort of men who have sex with men in Kisumu, Kenya. *Sexually Transmitted Infections* 2020;96(7):521-27. doi: 10.1136/sextrans-2019-054166
25. Ferre VM, Gbeasor-Komlanvi FA, Collin G, et al. Prevalence of human papillomavirus, human immunodeficiency virus, and other sexually transmitted infections among men who have sex with men in Togo: A national cross-sectional survey. *Clinical Infectious Diseases* 2019;69(6):1019-26. doi: 10.1093/cid/ciy1012
26. Twahirwa Rwema JO, Herbst S, Hamill MM, et al. Cross-sectional assessment of determinants of STIs among men who have sex with men and transgender women in Kigali, Rwanda. *Sexually Transmitted Infections* 2021 doi: 10.1136/sextrans-2020-054753
27. Mor Z, Shohat T, Goor Y, et al. Risk Behaviors and sexually transmitted diseases in gay and heterosexual men attending an STD clinic in Tel Aviv, Israel: a cross-sectional study. *The Israel Medical Association Journal (IMAJ)* 2012;14:147-51.
28. Hancali A, Bellaji B, Jennane S, et al. Prevalence of chlamydia trachomatis and neisseria gonorrhoeae among MSM in Morocco. *Sexually Transmitted Infections* 2019;95(Supplement 1):A244. doi: 10.1136/sextrans-2019-sti.614

29. Zucker R, Gaisa M, Sigel K, et al. Triple site sexually transmitted infection testing as a crucial component of surveillance for men who have sex with men: A prospective cohort study. *International Journal of STD and AIDS* 2022;33(2):114-22. doi: 10.1177/09564624211047477
30. Rehan N, Bokhari A, Nizamani NM, et al. National study of reproductive tract infections among high risk groups of Lahore and Karachi. *Journal of the College of Physicians and Surgeons Pakistan* 2009;19(4):228-31.
31. Brahmam GNV, Kodavalla V, Rajkumar H, et al. Sexual practices, HIV and sexually transmitted infections among self-identified men who have sex with men in four high HIV prevalence states of India. *AIDS* 2008;22(SUPPL. 5):S45-S57.
32. Hawkes S, Collumbien M, Platt L, et al. HIV and other sexually transmitted infections among men, transgenders and women selling sex in two cities in Pakistan: A cross-sectional prevalence survey. *Sexually Transmitted Infections* 2009;85(SUPPL. 2):ii8-ii16.
33. Vandana WV, Saxena NK. A study of the prevalence of gonorrhea among high risk population in Hyderabad, A.P. *Indian Journal of Public Health Research and Development* 2013;4(1):216-20.
34. Chunchanur SK, Shwetha JV, Ambica R, et al. Gonorrhea in Men having sex with men (MSM): A study from tertiary care centre, Bangalore, India. *BMC Infectious Diseases Conference: International Science Symposium on HIV and Infectious Diseases, ISSHID* 2019;20(Supplement 1) doi: 10.1186/s12879-020-05038-y
35. Safren SA, Devaleenal B, Biello KB, et al. Geographic and behavioral differences associated with sexually transmitted infection prevalence among Indian men who have sex with men in Chennai and Mumbai. *International Journal of STD and AIDS* 2021;32(2):144-51. doi: 10.1177/0956462420943016
36. Chau CT, Ho KM, Ho CK. Screening of asymptomatic *Chlamydia trachomatis* and *Neisseria gonorrhoeae* infections among men who have sex with men in Hong Kong. *Hong Kong Journal of Dermatology and Venereology* 2016;24(3):113-19.
37. Wei C, Guadamuz TE, Stall R, et al. STD prevalence, risky sexual behaviors, and sex with women in a national sample of Chinese men who have sex with men. *American Journal of Public Health* 2009;99(11):1978-81.
38. Girault P, Saidel T, Song N, et al. HIV, STIs, and sexual behaviors among men who have sex with men in Phnom Penh, Cambodia. *AIDS Education and Prevention* 2004;16(1):31-44.
39. Jiang J, Cao N, Zhang J, et al. High prevalence of sexually transmitted diseases among men who have sex with men in Jiangsu Province, China. *Sexually Transmitted Diseases* 2006;33(2):118-23.
40. Hoang TV, Tuan NA, Mills SJ, et al. Results from the HIV/STI Integrated Biological and Behavioral Surveillance (IBBS) in Vietnam: 2005-2006. *IBBS Viet Nam* 2006
41. Pattanasin S, Dunne EF, Wasinrapee P, et al. Screening for *Chlamydia trachomatis* and *Neisseria gonorrhoeae* infection among asymptomatic men who have sex with men in Bangkok, Thailand. *International Journal of STD and AIDS* 2018;29(6):577-87.
42. Tongtoyai J, Todd CS, Chonwattana W, et al. Prevalence and Correlates of *Chlamydia trachomatis* and *Neisseria gonorrhoeae* by Anatomic Site Among Urban Thai Men Who Have Sex With Men. *Sexually Transmitted Diseases* 2015;42(8):440-9.
43. Guo W, Wu ZY, Song AJ, et al. Impact of HIV/sexually transmitted infection testing on risky sexual behaviors among men who have sex with men in Langfang, China. *Chinese Medical Journal* 2013;126(7):1257-63.
44. Morineau G, Nugrahini N, Riono P, et al. Sexual risk taking, STI and HIV prevalence among men who have sex with men in six Indonesian cities. *AIDS and behavior* 2011;15(5):1033-44.
45. Prabawanti C, Bollen L, Palupy R, et al. HIV, sexually transmitted infections, and sexual risk behavior among transgenders in Indonesia. *AIDS and behavior* 2011;15(3):663-73.
46. Jung M, Lee J, Kwon DS, et al. Comparison of sexual risky factors of men who have sex with men and sex-buying men as groups vulnerable to sexually transmitted diseases. *Journal of Preventive Medicine and Public Health* 2012;45(3):156-63.
47. Fu GF, Jiang N, Hu HY, et al. The epidemic of HIV, syphilis, chlamydia and gonorrhea and the correlates of sexual transmitted infections among men who have sex with men in Jiangsu, China, 2009. *PLoS ONE* 2015;10 (3) (e0118863)
48. Pham QD, Nguyen TV, Hoang CQ, et al. Prevalence of HIV/STIs and associated factors among men who have sex with men in An Giang, Vietnam. *Sexually Transmitted Diseases* 2012;39(10):799-806.
49. Wickersham JA, Gibson BA, Bazazi AR, et al. Prevalence of Human Immunodeficiency Virus and Sexually Transmitted Infections among Cisgender and Transgender Women Sex Workers in Greater Kuala Lumpur, Malaysia: Results from a Respondent-Driven Sampling Study. *Sexually Transmitted Diseases* 2017;44(11):663-70.
50. Budkaew J, Chumworathayi B, Pientong C, et al. Prevalence and factors associated with gonorrhea infection with respect to anatomic distributions among men who have sex with men. *PLoS ONE* 2019;14 (4) (e0211682)
51. Chen JS, Levintow SN, Tran HV, et al. HIV and STI prevalence and testing history among men who have sex with men in Hanoi, Vietnam. *International Journal of STD and AIDS* 2022;33(2):193-201. doi: 10.1177/09564624211060185
52. Hiransuthikul A, Sungsing T, Jantarapakde J, et al. Correlations of chlamydia and gonorrhoea among pharyngeal, rectal and urethral sites among Thai men who have sex with men: Multicentre community-led test and treat cohort in Thailand. *BMJ Open* 2019;9(6) doi: 10.1136/bmjopen-2018-028162
53. Yang LG, Zhang XH, Zhao PZ, et al. Gonorrhea and chlamydia prevalence in different anatomical sites among men who have sex with men: A cross-sectional study in Guangzhou, China. *BMC Infectious Diseases* 2018;18 (1)(675)
54. Adamson PC, Bhatia R, Tran KDC, et al. Prevalence, anatomic distribution, and correlates of *Chlamydia trachomatis* and *Neisseria gonorrhoeae* infections among a cohort of men who have sex with men in Hanoi, Vietnam. *Sexually transmitted diseases* 2022;11 doi: 10.1097/OLQ.0000000000001626
55. Songtaweesin WN, Pornpaisalsakul K, Kawichai S, et al. Sexually transmitted infections incidence in young Thai men who have sex with men and transgender women using HIV pre-exposure prophylaxis. *International Journal of STD and AIDS* 2022 doi: 10.1177/09564624211056749
56. Zhou Y, Cai YM, Li SL, et al. Anatomical site prevalence and genotypes of *Chlamydia trachomatis* infections among men who have sex with men: A multi-site study in China. *BMC Infectious Diseases* 2019;19(1) doi: 10.1186/s12879-019-4664-1
57. Clark JL, Caceres CF, Lescano AG, et al. Prevalence of same-sex sexual behavior and associated characteristics among low-income urban males in Peru. *PloS ONE* 2007;2(1):e778.
58. Wong HT, Wong KH, Lee SS, et al. Community-Based Surveys for Determining the Prevalence of HIV, Chlamydia, and Gonorrhoea in Men Having Sex with Men in Hong Kong. *Journal of Sexually Transmitted Diseases Print* 2013 2013:958967.
59. Putu Yuda Hananta I, Van Dam AP, Bruisten SM, et al. Gonorrhea in Indonesia: High prevalence of asymptomatic urogenital gonorrhea but no circulating extended spectrum cephalosporins-resistant neisseria gonorrhoeae strains in Jakarta, Yogyakarta, and Denpasar, Indonesia. *Sexually Transmitted Diseases* 2016;43(10):608-16.

60. Soto RJ, Ghee AE, Nunez CA, et al. Sentinel surveillance of sexually transmitted infections/HIV and risk behaviors in vulnerable populations in 5 Central American countries. *Journal of Acquired Immune Deficiency Syndromes* 2007;46(1):101-11.
61. Clark JL, Lescano AG, Konda KA, et al. Syndromic management and STI control in urban Peru. *PLoS ONE* 2009;4 (9) (e7201)
62. Konda KA, Lescano AG, Celentano DD, et al. In peru, reporting male sex partners imparts significant risk of incident HIV/sexually transmitted infection: All men engaging in same-sex behavior need prevention services. *Sexually Transmitted Diseases* 2013;40(7):569-74.
63. Garcia JI, Sabido M, Nikiforov M, et al. The UALE project: a cross-sectional approach for trends in HIV/STI prevalence among key populations attending STI clinics in Guatemala. *BMJ Open* 2018;8(9):e022632.
64. Perez-Brumer AG, Konda KA, Salvatierra HJ, et al. Prevalence of HIV, STIs, and Risk Behaviors in a Cross-Sectional Community- and Clinic-Based Sample of Men Who Have Sex with Men (MSM) in Lima, Peru. *PLoS ONE* 2013;8 (4) (e59072)
65. Figueroa JP, Weir SS, Jones-Cooper C, et al. High HIV prevalence among men who have sex with men in Jamaica is associated with social vulnerability and other sexually transmitted infections. *West Indian Medical Journal* 2013;62(4):286-91.
66. Creswell J, Guardado ME, Lee J, et al. HIV and STI control in El Salvador: Results from an integrated behavioural survey among men who have sex with men. *Sexually Transmitted Infections* 2012;88(8):633-38.
67. Leon SR, Segura ER, Klausner JD, et al. High rates of chlamydia and gonorrhea infection in anal and pharyngeal sites in men who have sex with men (MSM) and transgender women (TW) in Lima, Peru. *Sexually Transmitted Infections Conference: STI and AIDS World Congress* 2013;89
68. Castillo R, Konda KA, Leon SR, et al. HIV and Sexually Transmitted Infection Incidence and Associated Risk Factors among High-Risk MSM and Male-to-Female Transgender Women in Lima, Peru. *Journal of Acquired Immune Deficiency Syndromes* 2015;69(5):567-75.
69. Cunha CB, Friedman RK, de Boni RB, et al. *Chlamydia trachomatis, Neisseria gonorrhoeae* and syphilis among men who have sex with men in Brazil. *BMC public health* 2015;15:686.
70. Allan-Blitz LT, Leon SR, Bristow CC, et al. High prevalence of extra-genital chlamydial or gonococcal infections among men who have sex with men and transgender women in Lima, Peru. *International Journal of STD and AIDS* 2017;28(2):138-44.
71. Galarraga O, Sosa-Rubi SG, Gonzalez A, et al. The disproportionate burden of HIV and STIs among male sex workers in Mexico City and the rationale for economic incentives to reduce risks. *Journal of the International AIDS Society* 2014;17 (19218)
72. Morales-Miranda S, Alvarez B, Manzanero M. Prevalence of HIV/STIs and risk behavior in men who have a sex with men, Belize, 2012. *Sexually Transmitted Infections Conference: STI and AIDS World Congress* 2013;89
73. Kojima N, Park H, Konda KA, et al. The PICASSO Cohort: Baseline characteristics of a cohort of men who have sex with men and male-to-female transgender women at high risk for syphilis infection in Lima, Peru. *BMC Infectious Diseases* 2017;17 (1) (255)
74. Cabeza J, Segura ER, Montano SM, et al. Extra-genital and urethral *chlamydia trachomatis* and *Neisseria gonorrheae* prevalence and associated risk factors in men who have sex with men and transgender women in Lima, Peru. *Sexually Transmitted Infections* 2015;2):A177.
75. Passaro RC, Segura ER, Perez-Brumer A, et al. Body Parts Matter: Social, Behavioral, and Biological Considerations for Urethral, Pharyngeal, and Rectal Gonorrhea and Chlamydia Screening among MSM in Lima, Peru. *Sexually Transmitted Diseases* 2018;45(9):607-14.
76. Chow JY, Konda KA, Calvo GM, et al. Demographics, Behaviors, and Sexual Health Characteristics of High Risk Men Who Have Sex with Men and Transgender Women Who Use Social Media to Meet Sex Partners in Lima, Peru. *Sexually Transmitted Diseases* 2017;44(3):143-48.
77. Mendizabal-Burastero R, Vargas C, Galindo-Arandi C, et al. Anal and oropharyngeal STI surveillance among men who have sex with men in Guatemala. *Sexually Transmitted Infections* 2015;2):A176.
78. Grinsztejn B, Jalil EM, Monteiro L, et al. Unveiling of HIV dynamics among transgender women: a respondent-driven sampling study in Rio de Janeiro, Brazil. *The Lancet HIV* 2017;4(4):e169-e76.
79. Bristow CC, Espinosa da Silva C, Vera AH, et al. Prevalence of bacterial sexually transmitted infections and coinfection with HIV among men who have sex with men and transgender women in Tijuana, Mexico. *International Journal of STD and AIDS* 2021;32(8):751-57. doi: 10.1177/0956462420987757
80. Moriarty KE, Segura ER, Gonzales W, et al. Assessing Sexually Transmitted Infections and HIV Risk Among Transgender Women in Lima, Peru: Beyond Behavior. *LGBT health* 2019;6(7):370-76. doi: 10.1089/lgbt.2018.0087
81. Jean Louis F, Galbaud G, Leonard M, et al. Prevalence of *Neisseria gonorrhoeae* and *Chlamydia trachomatis* in men having sex with men in Port-au-Prince, Haiti: A cross-sectional study. *BMJ Open* 2020;10(3) doi: 10.1136/bmjopen-2019-033976
82. Lister NA, Smith A, Read T, et al. Testing men who have sex with men for *Neisseria gonorrhoeae* and *Chlamydia trachomatis* prior to the introduction of guidelines at an STD clinic in Melbourne. *Sexual health* 2004;1(1):47-50.
83. Lister NA, Smith A, Tabrizi S, et al. Screening for *Neisseria gonorrhoeae* and *Chlamydia trachomatis* in Men Who Have Sex with Men at Male-only Saunas. *Sexually Transmitted Diseases* 2003;30(12):886-89.
84. Hamlyn E. Is screening for sexually transmitted infections in men who have sex with men who receive non-occupational HIV post-exposure prophylaxis worthwhile? *Sexually Transmitted Infections* 2006;82(1):21-23. doi: 10.1136/sti.2005.014662
85. Jin F, Prestage GP, Zablotska I, et al. High rates of sexually transmitted infections in HIV positive homosexual men: Data from two community based cohorts. *Sexually Transmitted Infections* 2007;83(5):397-99.
86. Lister NA, Smith A, Fairley CK. Introduction of screening guidelines for men who have sex with men at an STD clinic, the Melbourne Sexual Health Centre, Australia. *Sexual health* 2005;2(4):241-44.
87. Ryder N, Lockart IG, Bourne C. Is screening asymptomatic men who have sex with men for urethral gonorrhoea worthwhile? *Sexual Health* 2010;7(1):90-91.
88. Goddard SL, Poynten IM, Petoumenous K, et al. Prevalence, incidence and predictors of anal *Chlamydia trachomatis*, anal *Neisseria gonorrhoeae* and syphilis among older gay and bisexual men in the longitudinal Study for the Prevention of Anal Cancer (SPANC). *Sexually Transmitted Infections* 2019;24:24.
89. Cornelisse VJ, Zhang L, Law M, et al. Concordance of gonorrhoea of the rectum, pharynx and urethra in same-sex male partnerships attending a sexual health service in Melbourne, Australia. *BMC Infectious Diseases* 2018;18 (1) (95)
90. Martin-Sanchez M, Case R, Fairley C, et al. Trends and differences in sexual practices and sexually transmitted infections in men who have sex with men only (MSMO) and men who have sex with men and women (MSMW): A repeated cross-sectional study in Melbourne, Australia. *BMJ Open* 2020;10(11) doi: 10.1136/bmjopen-2020-037608

91. Chow EPF, Walker S, Read TRH, et al. Self-Reported Use of Mouthwash and Pharyngeal Gonorrhoea Detection by Nucleic Acid Amplification Test. *Sexually Transmitted Diseases* 2017;44(10):593-95.
92. Cornelisse VJ, Fairley CK, Phillips T, et al. Fuckbuddy partnerships among men who have sex with men - A marker of sexually transmitted infection risk. *International Journal of STD and AIDS* 2018;29(1):44-50.
93. Ong JJ, Fethers K, Howden BP, et al. Asymptomatic and symptomatic urethral gonorrhoea in men who have sex with men attending a sexual health service. *Clinical Microbiology & Infection* 2017;23(8):555-59.
94. Cornelisse VJ, Fairley CK, Stoove M, et al. Evaluation of preexposure (PrEP) eligibility criteria, using sexually transmissible infections as markers of human immunodeficiency virus (HIV) risk at enrollment in PrEPX, a large Australian HIV PrEP trial. *Clinical Infectious Diseases* 2018;67(12):1847-52.
95. Saxton PJW, Azariah S, Cavadino A, et al. Adherence, Sexual Behavior and Sexually Transmitted Infections in a New Zealand Prospective PrEP Cohort: 12 Months Follow-up and Ethnic Disparities. *AIDS and behavior* 2022;15 doi: 10.1007/s10461-022-03617-5
96. Tabesh M, Fairley CK, Hocking JS, et al. Comparison of the patterns of chlamydia and gonorrhoea at the oropharynx, anorectum and urethra among men who have sex with men. *Sexually transmitted infections* 2022;98(1):11-16. doi: 10.1136/sextrans-2020-054632
97. Badman SG, Willie B, Narokobi R, et al. A diagnostic evaluation of a molecular assay used for testing and treating anorectal chlamydia and gonorrhoea infections at the point-of-care in Papua New Guinea. *Clinical Microbiology & Infection* 2018;11:11.
98. Hakim AJ, Iwamoto C, Badman SG, et al. High Prevalence of Chlamydia and Gonorrhea and the Need for Sexually Transmitted Infection Testing Among Men Who Have Sex With Men and Transgender Women in Papua New Guinea. *Sexually transmitted diseases* 2021;48(2):109-17. doi: 10.1097/OLQ.0000000000001300
99. Moncada J, Shayevich C, Philip SS, et al. Detection of *Chlamydia trachomatis* and *Neisseria gonorrhoeae* in Rectal and Oropharyngeal Swabs and Urine Specimens from Men Who Have Sex With Men with Abbott's M2000 RealTime. *Sexually Transmitted Diseases* 2015;42(11):650-1.
100. Sexton M, Baker J, Perkins R, et al. Self-administered *neisseria gonorrheae* and *chlamydia trachomatis* testing in the pharynx and rectum among men who have sex with men in washington, DC. *Sexually Transmitted Infections* 2011;1):A74-A75.
101. Benn PD, Rooney G, Carder C, et al. *Chlamydia trachomatis* and *Neisseria gonorrhoeae* infection and the sexual behaviour of men who have sex with men. *Sexually Transmitted Infections* 2007;83(2):106-12.
102. Leuridan E, Wouters K, Stalpaert M, et al. Male sex workers in Antwerp, Belgium: a descriptive study. *International Journal of STD & AIDS* 2005;16(11):744-8.
103. Bloomfield PJ, Kent C, Campbell D, et al. Community-based chlamydia and gonorrhea screening through the United States mail, San Francisco. *Sexually Transmitted Diseases* 2002;29(5):294-97.
104. Kim AA, Kent CK, Klausner JD. Risk factors for rectal gonococcal infection amidst resurgence in HIV transmission. *Sexually Transmitted Diseases* 2003;30(11):813-7.
105. Sethi G, Holden BM, Gaffney J, et al. HIV, sexually transmitted infections, and risk behaviours in male sex workers in London over a 10 year period. *Sexually Transmitted Infections* 2006;82(5):359-63.
106. Javanbakht M, Murphy R, Harawa NT, et al. Sexually transmitted infections and HIV prevalence among incarcerated men who have sex with men, 2000-2005. *Sexually Transmitted Diseases* 2009;36(SUPPL. 2):S17-S21.
107. Dilley JW, Loeb L, Casey S, et al. Treating Asymptomatic Sexually Transmitted Diseases at Anonymous HIV Counseling and Testing Sites. *Sexually Transmitted Diseases* 2003;30(12):874-75.
108. Russell CJ, Golub SA, Cohen DE, et al. Urine-based asymptomatic urethral gonorrhea and chlamydia screening and sexual risk-taking behavior in men who have sex with men in greater Boston. *AIDS Patient Care and STDs* 2007;21(3):205-11.
109. Vall-Mayans M, Villa M, Saravanya M, et al. Sexually transmitted *Chlamydia trachomatis, Neisseria gonorrhoeae,* and HIV-1 infections in two at-risk populations in Barcelona: female street prostitutes and STI clinic attendees. *International Journal of Infectious Diseases* 2007;11(2):115-22.
110. Kent CK, Chaw JK, Wong W, et al. Prevalence of rectal, urethral, and pharyngeal chlamydia and gonorrhea detected in 2 clinical settings among men who have sex with men: San Francisco, California, 2003. *Clinical Infectious Diseases* 2005;41(1):67-74.
111. Mayer KH, Ducharme R, Zaller N, et al. Unprotected sex, underestimated risk, undiagnosed HIV and sexually transmitted diseases among men who have sex with men accessing testing services in a new england bathhouse. . *JAIDS Journal of Acquired Immune Deficiency Syndromes* 2011;24
112. Annan NT, Sullivan AK, Nori A, et al. Rectal chlamydia--a reservoir of undiagnosed infection in men who have sex with men. *Sexually Transmitted Infections* 2009;85(3):176-9.
113. Bozicevic I, Rode OD, Lepej SZ, et al. Prevalence of sexually transmitted infections among men who have sex with men in Zagreb, Croatia. *AIDS and Behavior* 2009;13(2):303-09.
114. Baker J, Plankey M, Josayma Y, et al. The prevalence of rectal, urethral, and pharyngeal *neisseria gonorrheae* and *chlamydia trachomatis* among asymptomatic men who have sex with men in a prospective Cohort in Washington, D.C. *AIDS Patient Care and STDs* 2009;23(8):585-88.
115. Ota KV, Tamari IE, Smieja M, et al. Detection of *Neisseria gonorrhoeae* and *Chlamydia trachomatis* in pharyngeal and rectal specimens using the BD Probetec et system, the Gen-Probe Aptima Combo 2 assay and culture. *Sexually Transmitted Infections* 2009;85(3):182-86.
116. Bernstein KT, Stephens SC, Barry PM, et al. *Chlamydia trachomatis* and *Neisseria gonorrhoeae* transmission from the oropharynx to the Urethra among men who have sex with men. *Clinical Infectious Diseases* 2009;49(12):1793-97.
117. Klausner JD. Clinic-based testing for rectal and pharyngeal *Neisseria gonorrhoeae* and *Chlamydia trachomatis* infections by community-based organizations--five cities, United States, 2007. *MMWR* 2009;Morbidity and mortality weekly report. 58(26):716-19.
118. Mimiaga MJ, Mayer KH, Reisner SL, et al. Asymptomatic gonorrhea and chlamydial infections detected by nucleic acid amplification tests among boston area men who have sex with men. *Sexually Transmitted Diseases* 2008;35(5):495-98.
119. Fisher M, Wayal S, Smith H, et al. Home sampling for sexually transmitted infections and HIV in men who have sex with men: a prospective observational study. *PLoS ONE* 2015;10(4):e0120810. doi: 10.1371/journal.pone.0120810 [published Online First: 2015/04/08]
120. Soni S, Alexander S, Verlander N, et al. The prevalence of urethral and rectal *Mycoplasma genitalium* and its associations in men who have sex with men attending a genitourinary medicine clinic. *Sexually Transmitted Infections* 2009;86(1):21-4.

121. Freeman AH, Bernstein KT, Kohn RP, et al. Evaluation of self-collected versus clinician-collected swabs for the detection of *Chlamydia trachomatis* and *Neisseria gonorrhoeae* pharyngeal infection among men who have sex with men. *Sexually Transmitted Diseases* 2011;38(11):1036-9.
122. Cuypers WJ, Niekamp AM, Keesmekers R, et al. High prevalence of HIV, other sexually transmitted infections and risk profile in male commercial sex workers who have sex with men in the Netherlands. *Sexually Transmitted Infections* 2011;1):A127.
123. Dudareva-Vizule S, Haar K, Sailer A, et al. Prevalence of pharyngeal and rectal *Chlamydia trachomatis* and *Neisseria gonorrhoeae* infections among men who have sex with men in Germany. *Sexually Transmitted Infections* 2014;90(1):46-51.
124. Mayer KH, Wang L, Koblin B, et al. Concomitant socioeconomic, behavioral, and biological factors associated with the disproportionate HIV infection burden among Black men who have sex with men in 6 U.S. cities. *PLoS ONE* 2014;9 (1) (e87298)
125. Reinton N, Moi H, Olsen AO, et al. Anatomic distribution of *Neisseria gonorrhoeae*, *Chlamydia trachomatis* and *Mycoplasma genitalium* infections in men who have sex with men. *Sexual Health* 2013;10(3):199-203.
126. Park J, Marcus JL, Pandori M, et al. Sentinel surveillance for pharyngeal chlamydia and gonorrhea among men who have sex with men-San Francisco, 2010. *Sexually Transmitted Diseases* 2012;39(6):482-84.
127. Perkins R, Furness B, Hager M, et al. Targeted STD screening among high-risk men who have sex with men. *Sexually Transmitted Infections* 2011;1):A98.
128. Bozicevic I, Lepej SZ, Rode OD, et al. Prevalence of HIV and sexually transmitted infections and patterns of recent HIV testing among men who have sex with men in Zagreb, Croatia. *Sexually Transmitted Infections* 2012;88(7):539-44.
129. van Liere GA, Hoebe CJ, Niekamp AM, et al. Standard symptom- and sexual history-based testing misses anorectal *Chlamydia trachomatis* and *Neisseria gonorrhoeae* infections in swingers and men who have sex with men. *Sexually Transmitted Diseases* 2013;40(4):285-9.
130. Remis RS, Liu J, Loutfy MR, et al. Prevalence of Sexually Transmitted Viral and Bacterial Infections in HIV-Positive and HIV-Negative Men Who Have Sex with Men in Toronto. *PLoS ONE* 2016;11(7):e0158090.
131. Van Liere GAFS, Van Rooijen MS, Hoebe CJPA, et al. Prevalence of and factors associated with rectal-only chlamydia and gonorrhoea in women and in men who have sex with men. *PLoS ONE* 2015;10 (10) (e0140297)
132. Van Der Veer C, Van Rooijen MS, Himschoot M, et al. *Trichomonas vaginalis* and *Mycoplasma genitalium*: Age-specific prevalence and disease burden in men attending a sexually transmitted infections clinic in Amsterdam, the Netherlands. *Sexually Transmitted Infections* 2016;92(1):83-85.
133. Gratrix J, Singh AE, Bergman J, et al. Prevalence and characteristics of rectal chlamydia and gonorrhea cases among men who have sex with men after the introduction of nucleic acid amplification test screening at 2 Canadian sexually transmitted infection clinics. *Sexually Transmitted Diseases* 2014;41(10):589-91.
134. Jeverica S, Unemo M, Mlakar B, et al. Prevalence of *Neisseria Gonorrhoeae* infection in two distinct men-who-have-sex-with-men (MSM) populations in Slovenia in 2012. *Sexually Transmitted Infections Conference: STI and AIDS World Congress* 2013;89
135. Bamberger DM, Graham G, Dennis L, et al. Extragenital Gonorrhea and Chlamydia among Men and Women According to Type of Sexual Exposure. *Sexually Transmitted Diseases* 2019;46(5):329-34. doi: 10.1097/OLQ.0000000000000967
136. Nelson LE, Tharao W, Husbands W, et al. The epidemiology of HIV and other sexually transmitted infections in African, Caribbean and Black men in Toronto, Canada. *BMC Infectious Diseases*;19(1):294.
137. Dolling DI, Desai M, McOwan A, et al. An analysis of baseline data from the PROUD study: An open-label randomised trial of pre-exposure prophylaxis. *Trials* 2016;17 (1) (163)
138. Closson K, Smith RV, Olarewaju G, et al. Associations between economic dependence, sexual behaviours, and sexually transmitted infections among young, Black, gay, bisexual and other men who have sex with men living with and without HIV in Jackson, Mississippi, USA. *Sexual Health* 2018;15(5):473-76.
139. Liu AY, Cohen SE, Vittinghoff E, et al. Preexposure prophylaxis for HIV infection integrated with municipal-and community-based sexual health services. *JAMA Internal Medicine* 2016;176(1):75-84.
140. Mena L, Crosby RA, Chamberlain N. Extragenital Chlamydia and Gonorrhea in Young Black Men Who Have Sex with Men: Missed Treatment Opportunities for Human Immunodeficiency Virus-Infected Men Who Have Sex with Men? *Sexually Transmitted Diseases* 2018;45(5):307-11.
141. Ruutel K, Lohmus L, Janes J. Internet-based recruitment system for HIV and STI screening for men who have sex with men in Estonia, 2013: analysis of preliminary outcomes. *Euro Surveillance: Bulletin Europeen sur les Maladies Transmissibles = European Communicable Disease Bulletin* 2015;20(15):16.
142. Tang EC, Vittinghoff E, Philip SS, et al. Quarterly screening optimizes detection of sexually transmitted infections when prescribing HIV preexposure prophylaxis. *AIDS (London, England)* 2020;34(8):1181-86. doi: 10.1097/QAD.0000000000002522
143. Calas A, Zemali N, Camuset G, et al. Prevalence of urogenital, anal, and pharyngeal infections with *Chlamydia trachomatis, Neisseria gonorrhoeae*, and *Mycoplasma genitalium:* a cross-sectional study in Reunion island. *BMC Infectious Diseases* 2021;21(1) doi: 10.1186/s12879-021-05801-9
144. Mustanski B, Feinstein BA, Madkins K, et al. Prevalence and Risk Factors for Rectal and Urethral Sexually Transmitted Infections from Self-Collected Samples among Young Men Who Have Sex with Men Participating in the Keep It Up! 2.0 Randomized Controlled Trial. *Sexually Transmitted Diseases* 2017;44(8):483-88.
145. Achterbergh RCA, Druckler S, van Rooijen MS, et al. Sex, drugs, and sexually transmitted infections: A latent class analysis among men who have sex with men in Amsterdam and surrounding urban regions, the Netherlands. *Drug and Alcohol Dependence* 2020;206 doi: 10.1016/j.drugalcdep.2019.06.028
146. Achterbergh RCA, de Vries HJC, Boyd A, et al. Identification and characterization of latent classes based on drug use among men who have sex with men at risk of sexually transmitted infections in Amsterdam, the Netherlands. *Addiction* 2020;115(1):121-33. doi: 10.1111/add.14774
147. Ceccarani C, Marangoni A, Severgnini M, et al. Rectal Microbiota Associated With *Chlamydia trachomatis* and *Neisseria gonorrhoeae* Infections in Men Having Sex With Other Men. *Frontiers in Cellular and Infection Microbiology* 2019;9 doi: 10.3389/fcimb.2019.00358
148. Grov C, Cain D, Rendina HJ, et al. Characteristics associated with urethral and rectal gonorrhea and chlamydia diagnoses in a us national sample of gay and bisexual men: Results from the one thousand strong panel. *Sexually Transmitted Diseases* 2016;43(3):165-71.
149. Chan PA, Crowley C, Rose JS, et al. A Network Analysis of Sexually Transmitted Diseases and Online Hookup Sites among Men Who Have Sex with Men. *Sexually Transmitted Diseases* 2018;45(7):462-68.

150. Low N, Bertisch B, Hauser C, et al. Factors associated with antimicrobial resistant gonorrhoea infections in men who have sex with men: case-control study. *Sexually Transmitted Infections* 2017;93:A28.
151. Wilson JD, Wallace HE, Loftus-Keeling M, et al. Swab-yourself Trial with Economic Monitoring and Testing for Infections Collectively (SYSTEMATIC): Part 1. A Diagnostic Accuracy and Cost-effectiveness Study Comparing Clinician-taken vs Self-taken Rectal and Pharyngeal Samples for the Diagnosis of Gonorrhea and Chlamydia. *Clinical Infectious Diseases* 2021;73(9):E3172-E80. doi: 10.1093/cid/ciaa1266
152. Druckler S, Van Rooijen MS, De Vries HJC. Chemsex among Men Who Have Sex with Men: A Sexualized Drug Use Survey among Clients of the Sexually Transmitted Infection Outpatient Clinic and Users of a Gay Dating App in Amsterdam, the Netherlands. *Sexually Transmitted Diseases* 2018;45(5):325-31.
153. Salow KR, Cohen AC, Bristow CC, et al. Comparing mail-in self-collected specimens sent via United States Postal Service versus clinic-collected specimens for the detection of *Chlamydia trachomatis* and *Neisseria gonorrhoeae* in extra-genital sites. *PLoS ONE* 2017;12 (12) (e0189515)
154. Abara WE, Llata EL, Schumacher C, et al. Extragenital Gonorrhea and Chlamydia Positivity and the Potential for Missed Extragenital Gonorrhea with Concurrent Urethral Chlamydia among Men Who Have Sex with Men Attending Sexually Transmitted Disease Clinics-Sexually Transmitted Disease Surveillance Network, 2015-2019. *Sexually Transmitted Diseases* 2020;47(6):361-68. doi: 10.1097/OLQ.0000000000001170
155. Barbee LA, Soge OO, Khosropour CM, et al. The Duration of Pharyngeal Gonorrhea: A Natural History Study. *Clinical Infectious Diseases* 2021;73(4):575-82. doi: 10.1093/cid/ciab071
156. Chapin-Bardales J, Johnson Jones ML, Kirkcaldy RD, et al. Pre-exposure Prophylaxis Use and Detected Sexually Transmitted Infections Among Men Who Have Sex With Men in the United States-National HIV Behavioral Surveillance, 5 US Cities, 2017. *J Acquir Immune Defic Syndr* 2020;85(4):430-35. doi: 10.1097/QAI.0000000000002482
157. Foschi C, Gaspari V, Sgubbi P, et al. Sexually transmitted rectal infections in a cohort of 'men having sex with men'. *Journal of Medical Microbiology* 2018;67(8):1050-57.
158. Johnson Jones ML, Chapin-Bardales J, Bizune D, et al. Extragenital Chlamydia and Gonorrhea Among Community Venue-Attending Men Who Have Sex with Men - Five Cities, United States, 2017. *MMWR* 2019;Morbidity and mortality weekly report. 68(14):321-25.
159. Harvey-Lavoie S, Apelian H, Labbe AC, et al. Community-Based Prevalence Estimates of *Chlamydia trachomatis* and *Neisseria gonorrhoeae* Infections Among Gay, Bisexual, and Other Men Who Have Sex With Men in Montreal, Canada. *Sexually transmitted diseases* 2021;48(12):939-44. doi: 10.1097/OLQ.0000000000001486
160. Fernandez-Huerta M, Barbera MJ, Esperalba J, et al. Prevalence of *Mycoplasma genitalium* and macrolide resistance among asymptomatic people visiting a point of care service for rapid STI screening: A cross-sectional study. *Sexually Transmitted Infections* 2020;96(4):300-05. doi: 10.1136/sextrans-2019-054124
161. Jansen K, Steffen G, Schuppe AK, et al. STI in times of PrEP: High prevalence of chlamydia, gonorrhea, and mycoplasma at different anatomic sites in men who have sex with men in Germany. *BMC Infectious Diseases* 2020;20(1) doi: 10.1186/s12879-020-4831-4
162. Rahib D, Bercot B, Delagreverie H, et al. Online self-sampling kits for human immunodeficiency virus and other sexually transmitted infections: Feasibility, positivity rates, and factors associated with infections in France. *International Journal of STD and AIDS* 2022;33(4):355-62. doi: 10.1177/09564624211066447
163. Assaf RD, Cunningham NJ, Adamson PC, et al. High proportions of rectal and pharyngeal chlamydia and gonorrhoea cases among cisgender men are missed using current CDC screening recommendations. *Sexually transmitted infections* 2022;25 doi: 10.1136/sextrans-2021-055361
164. Tyulenev YA, Guschin AE. Sexually transmitted infections in asymptomatic men who have sex with men. [Russian]. *Klinicheskaya Dermatologiya i Venerologiya* 2020;19(6):802-08. doi: 10.17116/klinderma202019061802
165. Zhang X, Wang C, Hengwei W, et al. Risk factors of HIV infection and prevalence of co-infections among men who have sex with men in Beijing, China. *AIDS* 2007;21(SUPPL. 8):S53-S57.
166. Hinkan S, Chuerduangphui J, Ekalaksananan T, et al. Anatomical site distribution and genotypes of *Chlamydia trachomatis* infecting asymptomatic men who have sex with men in northeast Thailand. *International Journal of STD and AIDS* 2018;29(9):842-50.
167. Dos Ramos Farias MS, Garcia MN, Reynaga E, et al. First report on sexually transmitted infections among trans (male to female transvestites, transsexuals, or transgender) and male sex workers in Argentina: High HIV, HPV, HBV, and syphilis prevalence. *International Journal of Infectious Diseases* 2011;15(9):e635-e40.
168. Pando MA, Balan IC, Marone R, et al. HIV and other sexually transmitted infections among men who have sex with men recruited by RDS in Buenos Aires, Argentina: High HIV and HPV infection. *PLoS ONE* 2012;7 (6) (e39834)
169. J Templeton D, Jin F, Imrie J, et al. Prevalence, incidence and risk factors for pharyngeal chlamydia in the community based Health in Men (HIM) cohort of homosexual men in Sydney, Australia. *Sexually transmitted infections* 2008;84:361-3. doi: 10.1136/sti.2008.032037
170. Guy RJ, Wand H, Franklin N, et al. Chlamydia trends in men who have sex with men attending sexual health services in Australia, 2004-2008. *Sexually Transmitted Diseases* 2011;38(4):339-46.
171. Lim MSC, Goller JL, Guy R, et al. Correlates of *Chlamydia trachomatis* infection in a primary care sentinel surveillance network. *Sexual Health* 2012;9(3):247-53.
172. Cornelisse VJ, Sherman CJ, Hocking JS, et al. Concordance of chlamydia infections of the rectum and urethra in same-sex male partnerships: A cross-sectional analysis. *BMC Infectious Diseases* 2017;17 (1) (22)
173. Manavi K, McMillan A, Young H. The prevalence of rectal chlamydial infection amongst men who have sex with men attending the genitourinary medicine clinic in Edinburgh. *International Journal of STD & AIDS* 2004;15(3):162-4.
174. Baud D, Jaton K, Bertelli C, et al. Low prevalence of *Chlamydia trachomatis* infection in asymptomatic young Swiss men. *BMC Infectious Diseases* 2008;8 (45)
175. Smelov V, Thomas P, Ouburg S, et al. Prevalence of genital *Chlamydia trachomatis* infections in Russia: Systematic literature review and multicenter study. *Pathogens and Disease* 2017;75 (7) (ftx081)
176. Dodge B, Van Der Pol B, Reece M, et al. Rectal self-sampling in non-clinical venues for detection of sexually transmissible infections among behaviourally bisexual men. *Sexual Health* 2012;9(2):190-91.

177. Van Rooijen MS, Van Der Loeff MFS, Morre SA, et al. Spontaneous pharyngeal *Chlamydia trachomatis* RNA clearance. A cross-sectional study followed by a cohort study of untreated STI clinic patients in Amsterdam, the Netherlands. *Sexually Transmitted Infections* 2015;91(3):157-64.
178. Lallemand A, Bremer V, Jansen K, et al. Prevalence of *Chlamydia trachomatis* infection in women, heterosexual men and MSM visiting HIV counselling institutions in North Rhine-Westphalia, Germany - should Chlamydia testing be scaled up? *BMC Infectious Diseases* 2016;16 (1) (610)
179. Lampkin D, Crawley A, Lopez TP, et al. Reaching suburban men who have sex with men for STD and HIV services through online social networking outreach: A public health approach. *Journal of Acquired Immune Deficiency Syndromes* 2016;72(1):73-78.
180. Rondeau P, Valin N, Decre D, et al. Chlamydia trachomatis screening in urine among asymptomatic men attending an STI clinic in Paris: A cross-sectional study. *BMC Infectious Diseases* 2019;19 (1) (31)
181. Samarasekara K, Fitzpatrick C, Finnerty F, et al. Pharyngeal *Chlamydia trachomatis* in HIV positive and HIV negative men who have sex with men (MSM). *HIV Medicine* 2021;22(SUPPL 2):106. doi: 10.1111/hiv.13131
